# SIK2 antagonizes postinfarction cardiac repair by driving macrophage proinflammatory and impairing efferocytosis

**DOI:** 10.1126/sciadv.adw6624

**Published:** 2026-10-16

**Authors:** Wu-Wei Rong, Zi-Qi Xu, Shuai Shao, Qian-Wan Deng, Meng-Yao Li, Jin-Qiu Wei, Yi-Hang Yang, Jia-Jia Zhao, Wen-Hui Zhai, Zhi-Wen Shang, Zhi Zhang, Wen-Yi Yang, Shao-Wen Liu, Ping-Jin Gao, Xiao-Dong Li, Ji-Guang Wang

**Affiliations:** ^1^ Department of Cardiovascular Medicine, National Research Center for Translational Medicine·Shanghai, State Key Laboratory of Medical Genomics, Shanghai Key Laboratory of Hypertension, Shanghai Institute of Hypertension, Ruijin Hospital, Shanghai Jiao Tong University School of Medicine, Shanghai, China.; ^2^ Department of Vascular & Cardiology, Shanghai First People's Hospital, Shanghai Jiao Tong University School of Medicine, Shanghai, China.

## Abstract

Macrophages play a key role in cardiac injury and repair after myocardial infarction (MI). Salt-inducible kinase 2 (SIK2), a member of the adenosine monophosphate–activated protein kinase family, is notably up-regulated in monocytes/macrophages of patients with MI and animal models. In this study, we investigated the role of SIK2 in macrophage-mediated inflammation and efferocytosis following MI. Macrophage-specific SIK2 deletion reduces infarct size and improves healing post-MI. Furthermore, SIK2-knockout macrophages decrease proinflammatory factor expression and enhance efferocytosis, thereby increasing repair factors expression. SIK2 associates with tumor necrosis factor receptor–associated factor 2 (TRAF2), promoting its phosphorylation and K63 polyubiquitylation, which exacerbates inflammation and hinders efferocytosis via the P38 and nuclear factor κB pathways. In contrast, the SIK2^T175A^ mutation exhibits no effect. Macrophage-specific SIK2 overexpression worsens cardiac changes after MI, whereas P38 inhibition reduces this effect. In addition, pharmacological inactivation of SIK2 substantially enhances cardiac repair after MI in mice. These findings highlight the crucial role of macrophage SIK2 in the regulation of inflammation and efferocytosis via TRAF2 polyubiquitination in MI, thereby presenting potential therapeutic targets.

## INTRODUCTION

Myocardial infarction (MI) is the leading cause of death worldwide (*1*). Despite advancements in reperfusion therapies, a considerable number of patients with MI still progress to heart failure owing to the loss of cardiomyocytes, insufficient healing, and adverse cardiac remodeling. These issues are primarily driven by excessive cardiac inflammation followed by active suppression and resolution (*2*). Immunosuppressive therapy using therapeutic monoclonal antibodies to neutralize interleukin-1β (IL-1β) has been effective in reducing inflammation and recurrent cardiovascular events; however, this systemic anti-inflammatory approach is associated with an increased risk of serious infection (*3*). Therefore, innovative approaches targeting inflammation are needed to reduce myocardial ischemic damage and enhance healing following MI.

Macrophages are pivotal in orchestrating both the initial inflammatory response and subsequent repair phase in MI. During the process of resolving acute inflammation, macrophages shift from a proinflammatory to a reparative phenotype, promoting cardiac healing (*4*). Uncontrolled inflammation damages surviving marginal cardiomyocytes and exacerbates matrix degradation, leading to the formation of scars with reduced tensile strength and heart rupture (*5*). In contrast, the suppression of proinflammatory macrophage activation limits cardiomyocyte necrosis and facilitates healing following MI (*6*).

The unsatisfactory outcomes of anti-inflammatory therapy trials, combined with the complex nature of MI, prompted us to consider additional factors. Necrotic and apoptotic cardiomyocytes are key determinants of the severity of adverse remodeling after MI (*7*). Dying cardiomyocytes activate inflammation and impede ideal repair by transmitting innate immune signals (*8*). Efferocytosis is the process of degradation of apoptotic cells through phagocytosis and endocytosis. Effective efferocytosis reduces the release of proinflammatory mediators from apoptotic cells and reprograms macrophages to express inflammation-resolving and reparative mediators (*9*). However, the mechanisms linking macrophage programming to efferocytosis remain unclear.

Targeting salt-inducible kinase 2 (SIK2) has been shown to have protective effects against inflammation, various cancers, and cardiac hypertrophy (*10*–*13*). Phosphorylation of threonine (Thr) residues in the Thr^175^ activation loop of SIK2 is essential for its catalytic activity (*14*). However, the mechanism underlying the association between SIK2 and MI remains unclear. This study aimed to investigate the role of SIK2 in macrophage-mediated inflammation and efferocytosis following MI and to elucidate the underlying molecular mechanisms. In addition, we aimed to evaluate the therapeutic potential of targeting SIK2 as an early intervention strategy to improve cardiac repair and function after MI.

## RESULTS

### SIK2 expression is elevated in patients and mice with MI

We first identified potential candidate genes associated with MI using bioinformatic transcriptomic analysis (GSE76701 and GSE206282) (*15*, *16*). Among the differentially expressed genes (DEGs), *SIK2* was notably up-regulated in patients with ischemic heart failure (Fig. 1A) and MI mouse model (Fig. 1B). In addition, in patients with dilated cardiomyopathy and heart failure (GSE120895) (*17*), *SIK2* expression also up-regulated in the heart tissue compared to that in nonfailing controls (Fig. 1C). We analyzed single-cell RNA sequencing (RNA-seq) data (GSE136088) from the left ventricular tissue of mice with MI and found that *Sik2* was mainly expressed in cardiac macrophages (fig. S1, A and B). Moreover, by examining the expression profiles of *Sik1*, *Sik2*, and *Sik3* in the ImmGen database, we identified *Sik2* as a promising candidate gene because of its high expression in macrophages (fig. S2, A to C). On the basis of mRNA transcription analysis (Fig. 1D) and protein detection (Fig. 1E) of peripheral blood mononuclear cells (PBMCs) from patients with MI, our data suggest that SIK2 expression was up-regulated earlier than the onset of myocardial tissue injury. No significant differences in age, sex, or comorbidities were observed between the MI and healthy control groups (table S1). Furthermore, the protein and mRNA levels of SIK2 were increased in PBMCs and ischemic myocardium of mice with MI (Fig. 1, F to J).

**Fig. 1.**
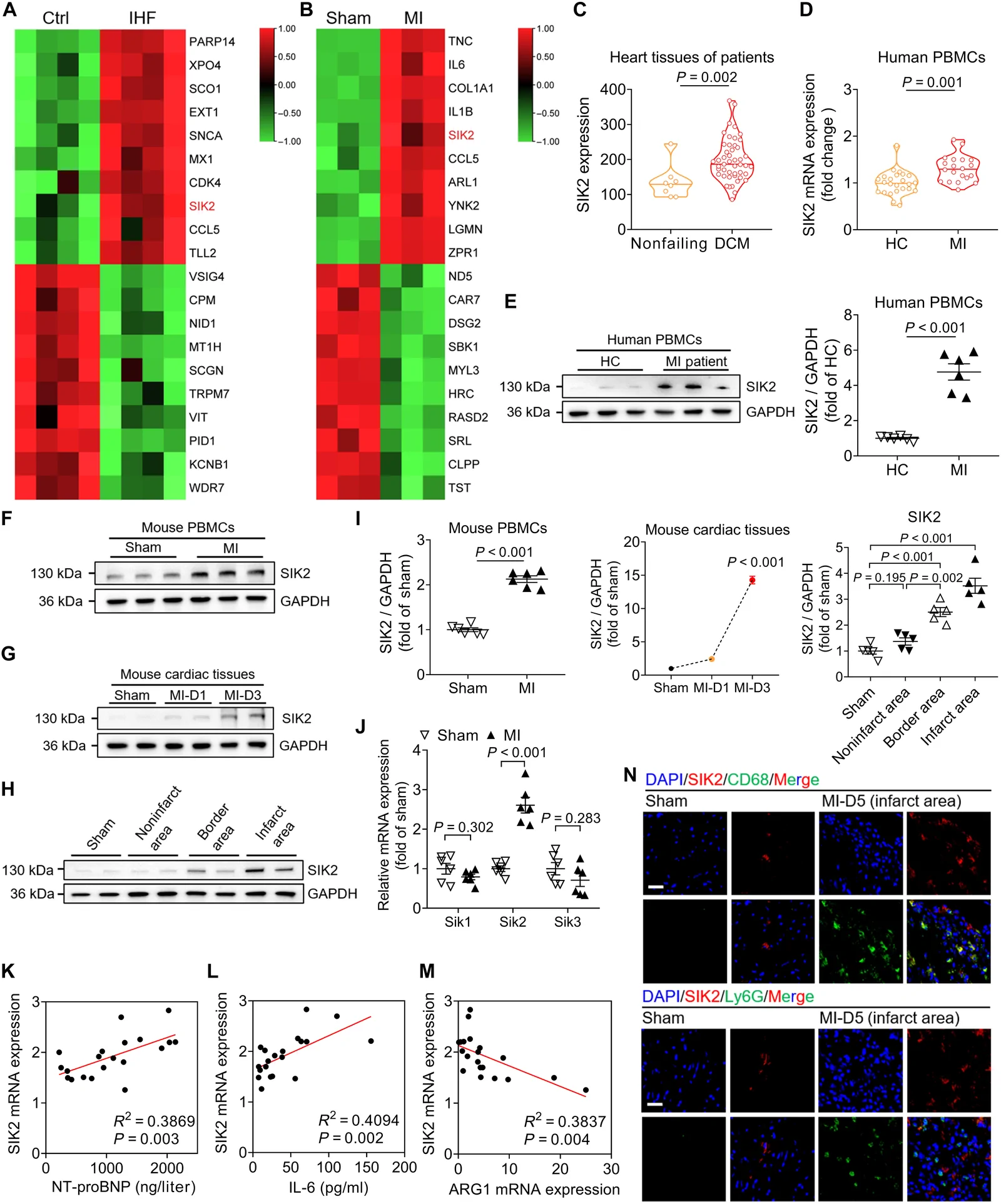
Elevated SIK2 levels correlate with adverse prognostic outcomes following MI. (**A**) Reevaluation transcription expression in the hearts of patients with nonfailing and ischemic end-stage heart failing (IHF) conditions using data from the GEO dataset GSE76701. (**B**) Reevaluation of transcription expression in heart tissue from mice, comparing control hearts to those 72 hours post-MI (GSE206282). (**C**) Reevaluation of *SIK2* gene expression in heart tissues from GSE120895 [8 nonfailing donors and 47 patients with dilated cardiomyopathy (DCM)]. (**D**) qPCR analysis of *SIK2* mRNA expression in PBMCs of patients with MI or healthy control (HC) individuals (*n* = 24 in HC; *n* = 20 in MI). (**E**) Immunoblotting analysis and quantification of SIK2 expression in PBMCs from patients with MI or HC (*n* = 6). Immunoblotting analysis of SIK2 expression in PBMCs (**F**) or ischemic cardiac tissues (**G** and **H**) of mice after sham operation or indicated days after MI. (**I**) Quantification of the immunoblot band intensity in (F) (left; *n* = 6), (G) (middle; *n* = 4), and (H) (right; *n* = 5). (**J**) qPCR analysis of *Sik1-3* mRNA expression in ischemic cardiac tissues of mice after MI or sham operation (*n* = 6). (**K** to **M**) Correlation of *SIK2* mRNA expression in PBMCs with plasma NT-proBNP (K; *n* = 20), IL-6 (L; *n* = 20), and PBMCs *ARG1* mRNA levels (M; *n* = 20) in patients with MI. (**N**) Immunofluorescence staining of SIK2 (red), CD68 (green), or Ly6G (green) and DAPI (4′,6-diamidino-2-phenylindole; blue) in heart tissues from WT mice at day 5 after MI or sham operation (scale bars, 40 μm).

Next, we examined the relationship between SIK2 expression levels in human PBMCs and various prognostic markers of MI in the plasma, including N-terminal pro-B-type natriuretic peptide (NT-proBNP) and IL-6, both of which are closely related to the prognosis and severity of MI (*18*). The potential effects of kidney and liver diseases were ruled out on the basis of the blood urea nitrogen, creatinine, alanine aminotransferase, and aspartate aminotransferase levels (table S2). In patients with MI, *SIK2* mRNA levels were positively correlated with plasma NT-proBNP and IL-6 concentrations (Fig. 1, K and L). However, Arginase 1 (ARG1), a marker associated with macrophage repair, was negatively correlated with *SIK2* mRNA expression (Fig. 1M). Furthermore, we evaluated the expression of SIK2 in the infarct area using immunofluorescence. Our findings revealed that SIK2 expression was primarily localized in CD68^+^ macrophages but not in Ly6G^+^ neutrophils, and its expression was higher in the infarct area than in the sham group (Fig. 1N). Meanwhile, we found that *Sik2* mRNA expression was markedly up-regulated in cardiac CD45^+^CD11b^+^F4/80^+^ macrophages after MI (fig. S3A). In addition, cultured macrophages exposed to hydrogen peroxide to mimic the oxidative stress observed in MI showed increased SIK2 expression (fig. S3, B to D). Furthermore, we found that the inflammatory stimulus lipopolysaccharide (LPS) not only elevated the total protein level of SIK2 but also markedly enhanced its phosphorylation. Crucially, the increase in SIK2 phosphorylation was closely correlated with the rise in total SIK2 protein levels (fig. S3E). Collectively, these findings suggest that macrophage SIK2 might participate in pathological processes following MI.

### Macrophage-specific SIK2 deficiency improves myocardial function and enhances cardiac repair after MI

Given that SIK2 is mainly up-regulated in cardiac macrophages, macrophage-specific SIK2-deficient mice (Sik2^F/F^; LysM^cre^, referred to as SIK2-cKO) were generated by crossing Sik2^F/F^ and LysM^cre^ mice. A marked reduction in SIK2 protein and mRNA expression was confirmed in cardiac macrophages of SIK2-cKO mice (fig. S4, A to C) compared to their Sik2^F/F^ littermates [referred to as wild-type (WT) mice]. To investigate the effects of SIK2 deficiency on MI, we compared the severity of MI in SIK2-cKO and WT mice. Echocardiography confirmed that SIK2-cKO mice had no preexisting heart abnormalities before MI surgery (table S3). Both SIK2-cKO and WT mice underwent MI, and cardiac function was assessed 7 and 14 days post-MI (Fig. 2A). Echocardiographic parameters, including ejection fraction (EF), fractional shortening (FS), left ventricular end-systolic volume (LVESV), and left ventricular end-systolic diameter (LVESD), showed significant improvements in SIK2-cKO mice compared to WT mice on days 7 and 14 post-MI (Fig. 2B). Furthermore, left ventricular chamber dilation was alleviated in SIK2-cKO mice, as demonstrated by the significant reduction in left ventricular end-diastolic volume (LVEDV) and left ventricular end-diastolic diameter (LVEDD) (Fig. 2B). Masson’s trichrome staining revealed a decrease in infarct size 14 days after MI in SIK2-cKO mice (Fig. 2C). Cardiac rupture was the leading cause of death in mice (fig. S5A). Notably, 52% of WT mice survived for 4 weeks post-MI, whereas the survival rate increased to 74% in SIK2-cKO mice (Fig. 2D).

**Fig. 2.**
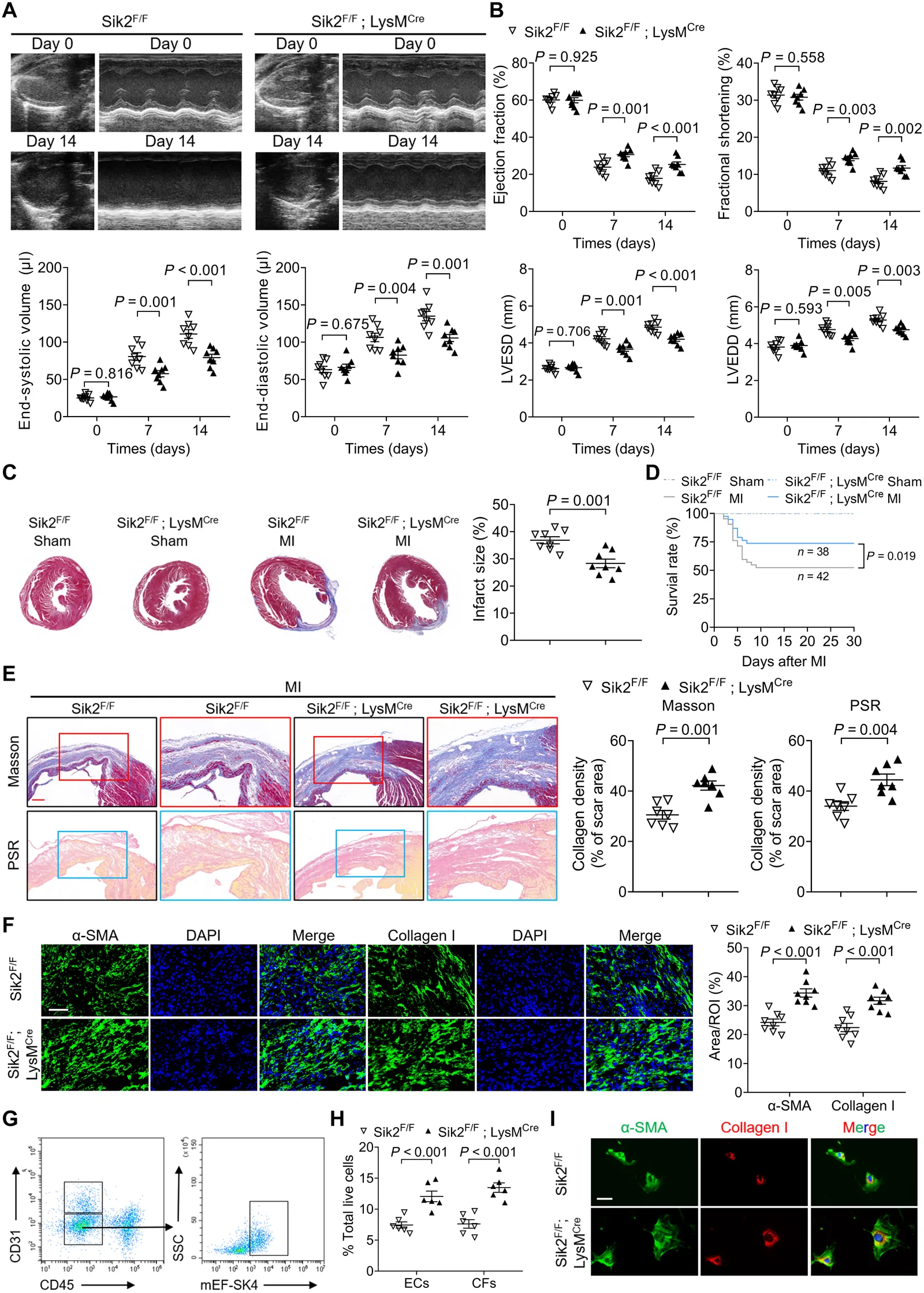
The absence of macrophage SIK2 alleviates myocardial dysfunction and adverse remodeling, improving long-term survival after MI. (**A**) Representative parasternal long-axis views and M-mode images. (**B**) Echocardiographic analysis of EF, FS, LVESV, LVEDV, LVESD, and LVEDD on days 0, 7, and 14 after MI or sham operation in Sik2^F/F^ and Sik2 ^F/F^; LysM^Cre^ mice (*n* = 8). (**C**) Masson’s trichrome staining for infarct size measurements in Sik2^F/F^ and Sik2^F/F^; LysM^Cre^ mice at day 14 after MI or sham operation (*n* = 8). (**D**) Post-MI survival analysis. *n* = 42 in Sik2^F/F^ MI; *n* = 38 in Sik2 ^F/F^; LysM^Cre^ MI. (**E**) Masson’s trichrome and Picrosirius red (PSR) staining of heart transverse sections to detect collagen within the scar of Sik2^F/F^ and Sik2 ^F/F^; LysM^Cre^ hearts at day 14 post-MI (scale bar, 100 μm). Collagen density measured as the total collagen area divided by scar area examined (*n* = 7). (**F**) Immunostaining analyses of α-SMA (green), Collagen I (green) and DAPI (blue) in the heart tissue at day 7 post-MI (*n* = 8) (scale bar, 40 μm). ROI indicates the region of interest. (**G**) Gating strategy for endothelial cells (ECs) and CFs in the infarcted heart. (**H**) Statistics of ECs and CFs in the infarcted heart at day 7 post-MI from Sik2^F/F^ and Sik2 ^F/F^; LysM^Cre^ mice (*n* = 6). (**I**) Representative fibroblasts immunostaining of α-SMA (green), Collagen I (red), and DAPI (blue). Scale bar, 20 μm.

On day 14 post-MI, SIK2-cKO mice showed reduced heart and lung weight-to-body weight ratios compared to WT mice, suggesting improved cardiac function (fig. S5, B and C). Optimal cardiac repair can reduce mortality and improve cardiac function after MI (*19*). Therefore, we analyzed the parameters of myocardial wound healing and myofibroblast differentiation after MI. Masson’s trichrome and Picrosirius red staining of cardiac papillary horizontal transverse sections showed that collagen density in infarcts was significantly higher in SIK2-cKO hearts than in WT hearts at 14 days post-MI (Fig. 2E). However, no significant differences in cardiac structural indicators were observed between SIK2-cKO and WT mice in the remote aera (fig. S6A). Optimal extracellular matrix formation in the infarct area is key to promoting cardiac wound healing (*20*). Immunofluorescence staining demonstrated that the expression levels of α–smooth muscle actin (α-SMA) and Collagen I were significantly higher in SIK2-cKO mice than in WT mice 7 days post-MI (Fig. 2F). Moreover, immunoblotting and quantitative polymerase chain reaction (qPCR) revealed significantly higher expression of α-SMA, Collagen I, and Collagen III in the infarct area of SIK2-cKO mice than in that of WT mice after MI (fig. S7, A and B). We used mouse embryonic fibroblast (MEF)–SK4, a specific marker for mouse cardiac fibroblasts (CFs), to isolate CFs from the infarct area while maintaining their functional phenotypes (Fig. 2G) (*5*). Flow cytometry revealed a higher abundance of CD45^−^CD31^+^ endothelial cells and CD45^−^CD31^−^MEFSK4^+^ CFs in the infarct areas of SIK2-cKO mice than in those of WT mice (Fig. 2H). In addition, CFs exhibited increased expression of α-SMA and Collagen I in SIK2-cKO mice compared to that in WT mice (Fig. 2I). Myocardial angiogenesis plays an important role in wound healing and cardiac remodeling (*21*). Immunoblotting and immunostaining demonstrated that the endothelial marker CD31 was significantly increased in the infarct region of SIK2-cKO mice 7 days post-MI (fig. S7, C and D). These findings suggest a more efficient cardiac repair in the absence of macrophage SIK2 post-MI.

### SIK2 loss-of-function attenuates inflammatory phenotype in infarct macrophages

Considering the critical role of macrophage-mediated inflammation in cardiac injury following MI, we investigated the characteristics of inflammation in vivo. Genome-wide gene expression profiles of myocardial macrophages from SIK2-cKO and WT mice on day 3 post-MI were analyzed in detail using RNA-seq. A total of 521 significantly up-regulated genes and 341 significantly down-regulated genes were detected in infarcted cardiac macrophages from SIK2-cKO mice compared to WT controls (Fig. 3A). Among the DEGs, we observed a notable down-regulation of key inflammatory genes, such as *Il-1α*, *Il-1β*, *Nos2*, *Il-6*, *Tlr2*, *Cxcl1*, *Cxcl2*, *Fas*, and *Il-12a*, along with a marked up-regulation of crucial anti-inflammatory genes, including *Arg1*, *Tgfbi*, *Il-10*, *Cd36*, *Cxcr4*, *Retnla*, *Cx3cr1*, *Pdgfc*, and *Ym1* (Fig. 3, B and C). The expression of key inflammation-associated genes was assessed in the infarcted regions. We confirmed that SIK2-cKO infarcts displayed down-regulation of proinflammatory genes, including *Il-1α*, *Il-1β*, *Il-6*, *Nos2*, *Cxcl-1*, *Tnf-α*, and *Mmp-9* (Fig. 3D). However, the expression of the reparative genes *Vegfa*, *Arg1*, *Tgfb1*, *Cd36*, and *Il-10* was higher in the infarcted regions of SIK2-cKO hearts than in the controls on day 5 post-MI (Fig. 3E). The proinflammatory protein NOS2 and tissue repair-associated protein ARG1 are recognized as functional markers of proinflammatory and anti-inflammatory macrophages, respectively (*22*, *23*). Coimmunofluorescence staining for NOS2/CD68 and ARG1/CD68 revealed that the proportion of NOS2-positive proinflammatory macrophages was lower, whereas the proportion of ARG1-positive anti-inflammatory macrophages was higher, in the infarct area of SIK2-cKO hearts than in that of WT mice (Fig. 3F). Simultaneously, we found that *Nos2* mRNA expression was markedly attenuated in SIK2-cKO cardiac CD45^+^CD11b^+^F4/80^+^ macrophages, concomitant with a pronounced up-regulation of *Arg1* mRNA expression (fig. S8A).

**Fig. 3.**
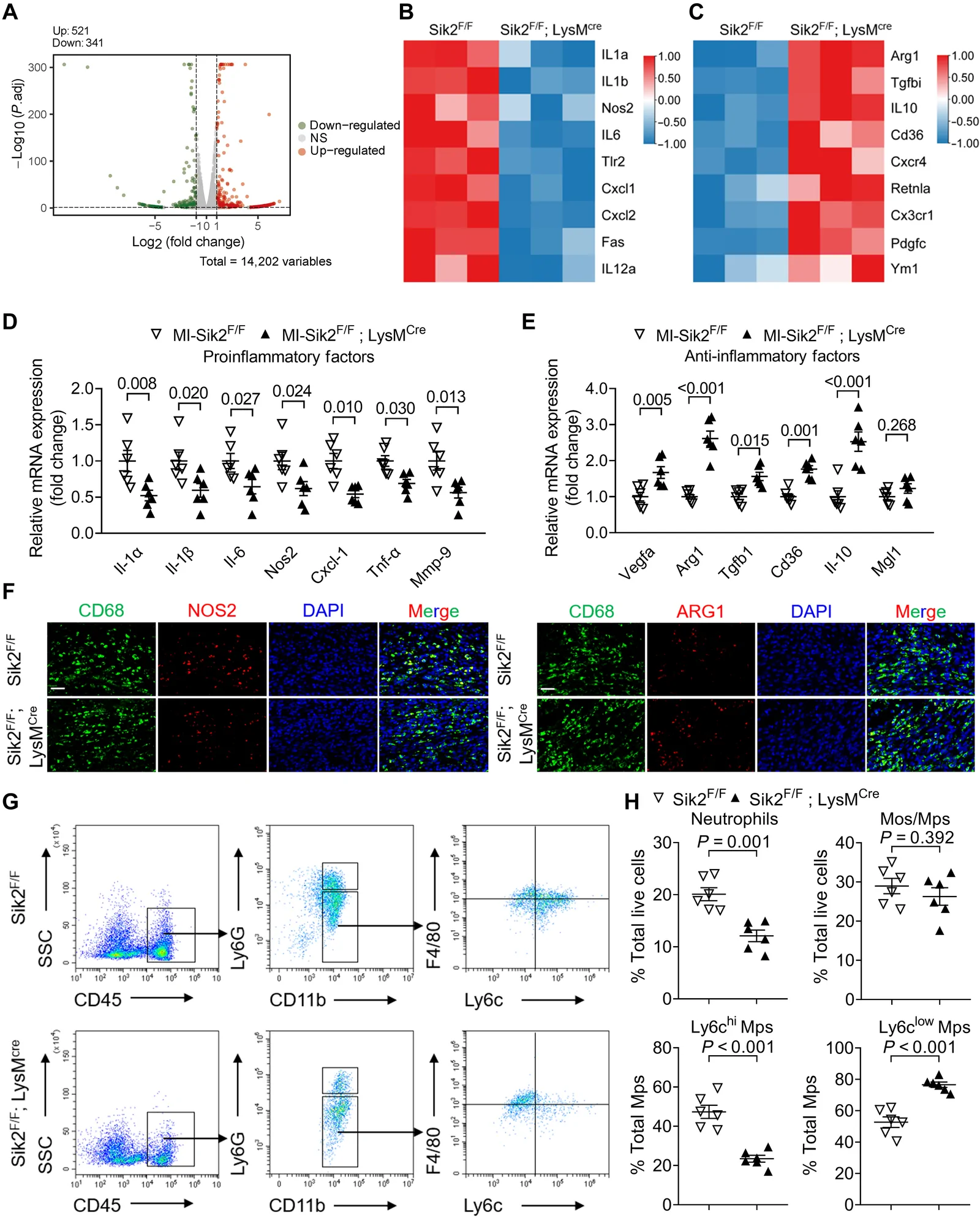
Reduced inflammatory phenotype in infarct macrophages with SIK2 deficiency. (**A**) Volcano plot displaying the DEGs (fold change ≥ 2 and *P*.adjust ≤ 0.05) between Sik2^F/F^; LysM^Cre^ cardiac macrophages and Sik2^F/F^ controls on day 3 post-MI. NS, not significant. (**B**) Heatmap of down-regulated DEGs relevant to inflammation in Sik2^F/F^; LysM^Cre^ cardiac macrophages on day 3 post-MI. (**C**) Heatmap of up-regulated DEGs relevant to anti-inflammation in Sik2^F/F^; LysM^Cre^ cardiac macrophages on day 3 post-MI. (**D** and **E**) mRNA expression levels of major proinflammatory and reparative factors in Sik2^F/F^ and Sik2^F/F^; LysM^Cre^ infarct hearts at day 5 post-MI (*n* = 6). (**F**) Coimmunostaining of CD68 (green), NOS2 (red), or ARG1 (red) and DAPI (blue) in MI hearts from Sik2^F/F^ and Sik2^F/F^; LysM^Cre^ mice. Transverse heart sections were prepared at day 5 post-MI (scale bars, 50 μm). (**G**) Gating strategy for the flow cytometry analysis of leukocyte population isolated from Sik2^F/F^ and Sik2 ^F/F^; LysM^Cre^ infarcts at day 5 post-MI. (**H**) Flow cytometry–based statistical analysis of neutrophils, monocytes/macrophages (Mos/Mps), and the percentage of Ly6C^high^ or Ly6C^low^ macrophages relative to the total macrophage population in Sik2^F/F^ and Sik2^F/F^; LysM^Cre^ hearts at day 5 post-MI (*n* = 6).

Next, we investigated the effect of macrophage-specific SIK2 deletion on cardiac immune cell profiles using flow cytometry on day 5 post-MI. Cardiac neutrophils and monocytes/macrophages were identified using previously reported gating strategies (*24*). We defined CD45^+^CD11b^+^Ly6G^−^F4/80^+^ cells as the total cardiac macrophage population and further separated and quantified subsets of macrophages based on the expression levels of Ly6C (Fig. 3G). As reported, CD45^+^CD11b^+^Ly6G^−^F4/80^+^Ly6C^high^ cells exhibit proinflammatory properties. Conversely, CD45^+^CD11b^+^Ly6G^−^F4/80^+^Ly6C^low^ cells primarily represent the reparative subsets (*25*). SIK2-cKO infarcts displayed a notable decrease in neutrophils and Ly6C^high^ macrophages, along with an increase in reparative Ly6C^low^ macrophages on day 5 after MI (Fig. 3H). We analyzed the immune cell profiles of the peripheral blood, bone marrow, and spleen 5 days post-MI. Although variations in specific immune cell populations were observed, no significant differences of monocyte proportion were found between the SIK2-cKO and WT mice (fig. S8, B to D). Collectively, these findings demonstrate that SIK2 deletion in macrophages decreases the proinflammatory phenotype and enhances the expression of reparative mediators post-MI.

### SIK2 deficiency improves cardiac recovery by promoting efferocytosis

The degree of myocardial apoptosis is related to myocardial necrosis or infarct size, which is the main factor influencing the prognosis (*26*). Terminal deoxynucleotidyl transferase–mediated deoxyuridine triphosphate nick end labeling (TUNEL) staining was performed 3 days post-MI to detect cardiomyocyte apoptosis in the border zone. The percentage of TUNEL-positive cardiomyocytes was significantly lower in SIK2-cKO mice than in WT mice post-MI (Fig. 4A). Previous studies have linked cardiomyocyte apoptosis to cardiac repair, highlighting the role of macrophage efferocytosis in the degradation of internalized apoptotic cardiomyocytes. This process limits the production of inflammatory cytokines and increases the release of repair mediators (*27*). The colocalization of macrophages and cardiomyocytes in myocardial tissue was investigated using the cardiomyocyte marker cardiac troponin I and the macrophage marker CD68 as surrogate indicators of in vivo efferocytosis (*7*). We found that the percentage of cardiomyocyte-containing macrophages in myocardial tissues was significantly increased in SIK2-cKO mice on day 3 after MI (Fig. 4B). Next, Prussian blue staining was used on infarcted heart sections as an independent readout of in vivo phagocytic activity (*28*). We observed an increase in iron deposition in the SIK2-cKO group, which indicates an enhanced phagocytic activity (fig. S9A). Meanwhile, we used flow cytometry to detect the cardiomyocyte-specific protein α-Actinin within macrophages isolated from infarcted hearts and identified those harboring α-Actinin^+^ material as phagocytes of cardiomyocytes. Analysis at day 3 post-MI revealed a significantly higher proportion of these α-Actinin^+^ macrophages in SIK2-cKO mice compared to controls, indicating that loss of SIK2 function enhances the capacity for cardiomyocyte phagocytosis in vivo (fig. S9B).

**Fig. 4.**
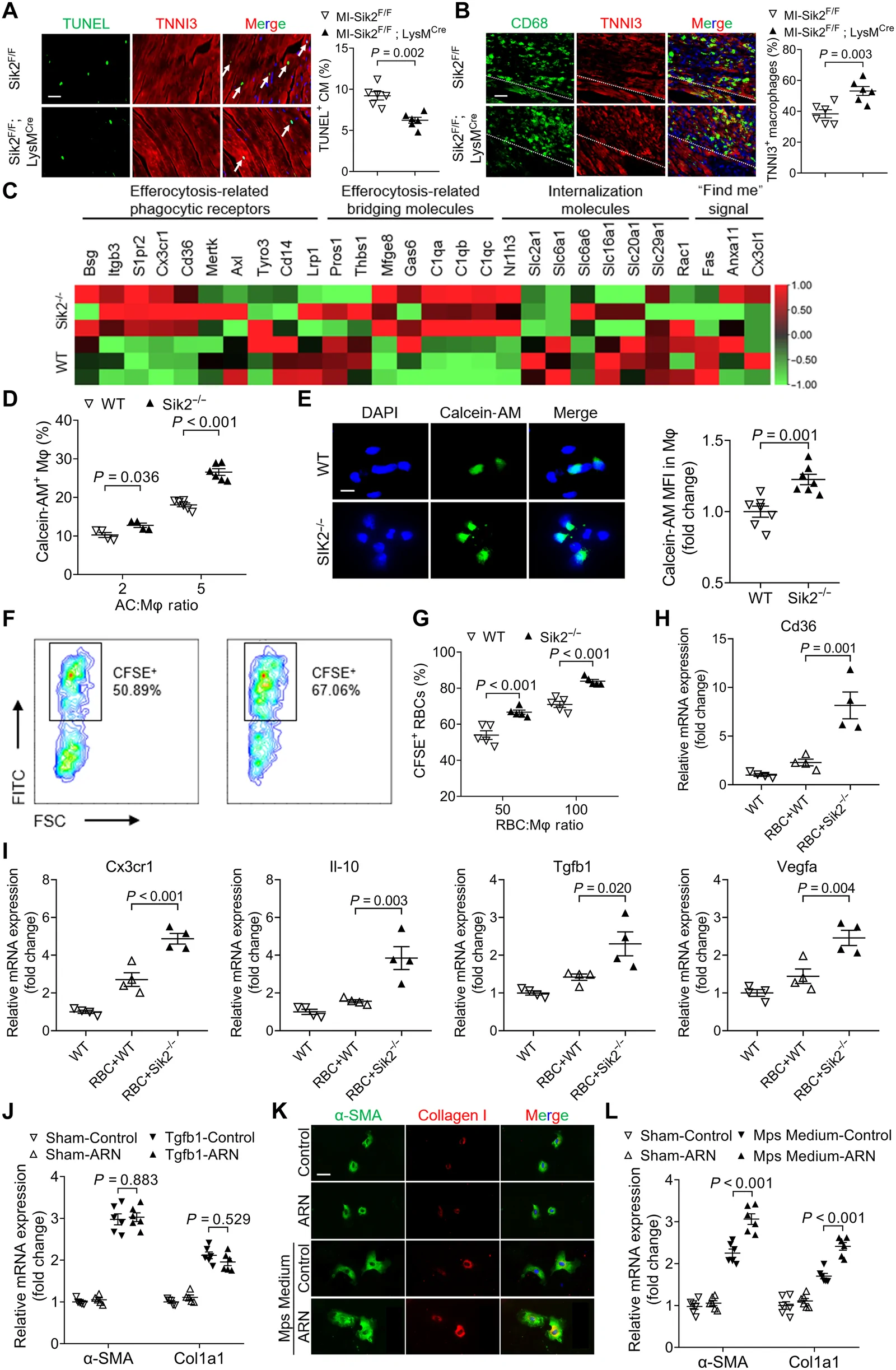
SIK2 deficiency improves cardiac recovery by promoting efferocytosis. (**A**) Representative TUNEL/TNNI3/DAPI staining of cardiomyocytes from Sik2^F/F^ and Sik2^F/F^; LysM^Cre^ mice on day 3 post-MI. White arrows indicate TUNEL^+^ (green) cardiomyocytes (red) and DAPI (blue) (scale bar, 40 μm). Percentage of TUNEL^+^ cardiomyocytes (*n* = 6). (**B**) Immunostaining shows colocalization of CD68^+^ macrophages with TNNI3^+^ cardiomyocytes (yellow). Scale bar, 40 μm. Macrophages positive for TNNI3 are scored as internalizing cardiomyocyte-derived proteins (*n* = 6). (**C**) Heatmap of efferocytosis-related DEGs in cardiac macrophages on day 3 post-MI. (**D**) WT or SIK2-knockout cardiac macrophages were incubated with Calcein-AM^+^ apoptotic cardiomyocytes (AC:Mφ = 2:1 or 5:1) for 1 hour. Efferocytosis was quantified as the total percentage of macrophages positive for Calcein-AM^+^ ACs (*n* = 4 for 2:1; *n* = 6 for 5:1). (**E**) Calcein-AM^+^ ACs were incubated with WT or SIK2-knockout macrophages (5:1) for 1 hour. Efferocytosis was analyzed by fluorescence microscopy for Calcein-AM mean fluorescence intensity (MFI) in macrophage (*n* = 7). (**F**) Flow cytometry analysis of efferocytosis in PMs for 2 hours. (**G**) PMs from WT and SIK2-cKO mice were treated with CFSE^+^ RBCs (RBC:Mφ = 50:1 or 100:1) for 2 hours. Phagocytic macrophages were quantified as the total percentage of macrophages positive for CFSE^+^ RBCs (*n* = 5). (**H** and **I**) qPCR analysis of *Cd36*, *Cx3cr1*, *Il-10*, *Tgfb1*, and *Vegfa* mRNA levels in macrophages cocultured with RBCs for 8 hours (*n* = 4). (**J**) qPCR analysis of *α-SMA* and *Col1a1* mRNA levels in control and ARN3236 (3 μmol)–treated CFs with or without *Tgfb1* (20 ng/ml) (*n* = 6). (**K**) Immunostaining staining of α-SMA (green), Collagen I (red), and DAPI (blue) in WT mouse CFs treated with macrophage-conditioned media (ARN3236-treated or control) after efferocytosis. Scale bar, 20 μm. (**L**) qPCR analysis of *α-SMA* and *Col1a1* mRNA levels in WT mouse CFs cultured with macrophage-conditioned media (ARN3236-treated or control) after efferocytosis (*n* = 6).

Efferocytosis involves recognition of apoptotic cells by phagocytic receptors, followed by phagocytosis and digestion within phagolysosomes (*29*). RNA-seq of cardiac macrophages revealed that SIK2 deficiency had a pronounced effect on the expression of phagocytic receptors and binding molecules involved in recognition of apoptotic cells, including *Itgb3*, *Cx3cr1*, *Cd36*, *Mfge8*, *C1qa*, *C1qb*, and *C1qc* (Fig. 4C). To investigate the role of SIK2 in efferocytosis directly, we cocultured apoptotic primary cardiomyocytes with mouse cardiac macrophages. SIK2-cKO macrophages exhibited enhanced efferocytosis of high-burden apoptotic cardiomyocytes. Flow cytometry– and microscopy-based measurements were used to validate these findings with calcein-AM–labeled cardiomyocytes (Fig. 4, D and E). In addition, we subjected peritoneal macrophages (PMs) to carboxyfluorescein diacetate succinimidyl ester (CFSE)–prelabeled red blood cells (RBCs) in varying proportions for 2 hours and measured the percentage of CFSE^+^ cells (phagocytic efficiency). Phagocytic efficiency was increased in SIK2-cKO macrophages (Fig. 4, F and G). Efferocytosis triggers phenotypic changes in macrophages, thereby promoting a reparative phenotype (*19*). We examined whether SIK2-cKO macrophages are more likely to adopt a reparative phenotype following efferocytosis. Our findings showed that efferocytosis induced a significantly higher expression of phagocytic receptors, *Cd36* and *Cx3cr1*, as well as reparative factors, such as *Il-10*, *Tgfb1*, and *Vegfa*, in SIK2-cKO macrophages than in WT macrophages (Fig. 4, H and I). The repair factors released by efferocytosis are beneficial for cardiac wound repair (*8*). The enhanced expression of *Cd36*, *Cx3cr1*, *Il-10*, *Tgfb1*, and *Vegfa* was also found in SIK2-cKO cardiac macrophages (fig. S9C).

To assess the cross-talk between macrophages and fibroblasts, we treated CFs with macrophages secreted after efferocytosis. ARN3236, an SIK2 kinase-specific inhibitor, was used (*13*). ARN treatment did not affect CF differentiation (Fig. 4J). However, the expression of α-SMA and Col1a1 in CFs was significantly increased in the ARN-treated macrophage medium compared to that in the control group (Fig. 4, K and L). These results collectively suggest that SIK2 inhibition promotes CF activation by enhancing reparative mediators through efferocytosis, thereby improving cardiac repair.

### NF-κB and P38 pathways are essential for SIK2-mediated proinflammatory macrophages and defective efferocytosis

To further elucidate the mechanism underlying the functional transformation of SIK2-deficient macrophages in cardiac injury after MI, we performed Kyoto Encyclopedia of Genes and Genomes (KEGG) pathway enrichment analysis of the DEGs between myocardial macrophages from SIK2-cKO and WT mice. The top 20 pathways with negatively altered gene expression were identified, with most of them linked to the nuclear factor κB (NF-κB) and tumor necrosis factor (TNF) signaling pathways (Fig. 5A). We analyzed the significantly altered genes that overlapped among the top 20 enriched pathways. Genes such as *Cxcl1*, *Cxcl2*, *Cxcl3*, *Ifng*, *Il-1b*, *Il-6*, *Mapk13*, *Nlrp3*, and *Ptgs2* are involved in mediating proinflammatory responses and recruiting leukocyte populations. Notably, these genes were decreased in SIK2-cKO cardiac macrophages after MI (Fig. 5B). To investigate the anti-inflammatory mechanism of SIK2-cKO macrophages, the top 500 DEGs were selected for transcription factor prediction analysis using transcriptional regulatory relationships revealed by the sentence-based text-mining (TRRUST) databases. These methods identified NFκB1 as the top transcription factor (Fig. 5C), suggesting that SIK2 deficiency inhibits NF-κB activation to down-regulate the inflammatory response in macrophages. Reduced nuclear translocation of P65-NF-κB was consistently observed in SIK2-cKO PMs by immunoblotting and immunofluorescence (Fig. 5D and fig. S10A). Western blot analysis revealed a significant reduction in the nuclear translocation of P65-NF-κB in SIK2-cKO cardiac macrophages compared to controls (fig. S10B). Furthermore, SIK2-cKO PMs and cardiac macrophages suppressed the production of inflammatory factors such as *Il-1β*, *Il-6*, *Nos2*, *Cxcl-1*, and *Mmp-9* (Fig. 5E and fig. S10C). ARN3236 was used to determine the role of SIK2 kinase activity in macrophages. Similar to the SIK2-cKO macrophages, ARN3236 treatment suppressed the production of inflammatory factors (Fig. 5F). In contrast, overexpression of SIK2 in macrophages using a lentivirus (LeSik2) increased the production of inflammatory cytokines (fig. S10D).

**Fig. 5.**
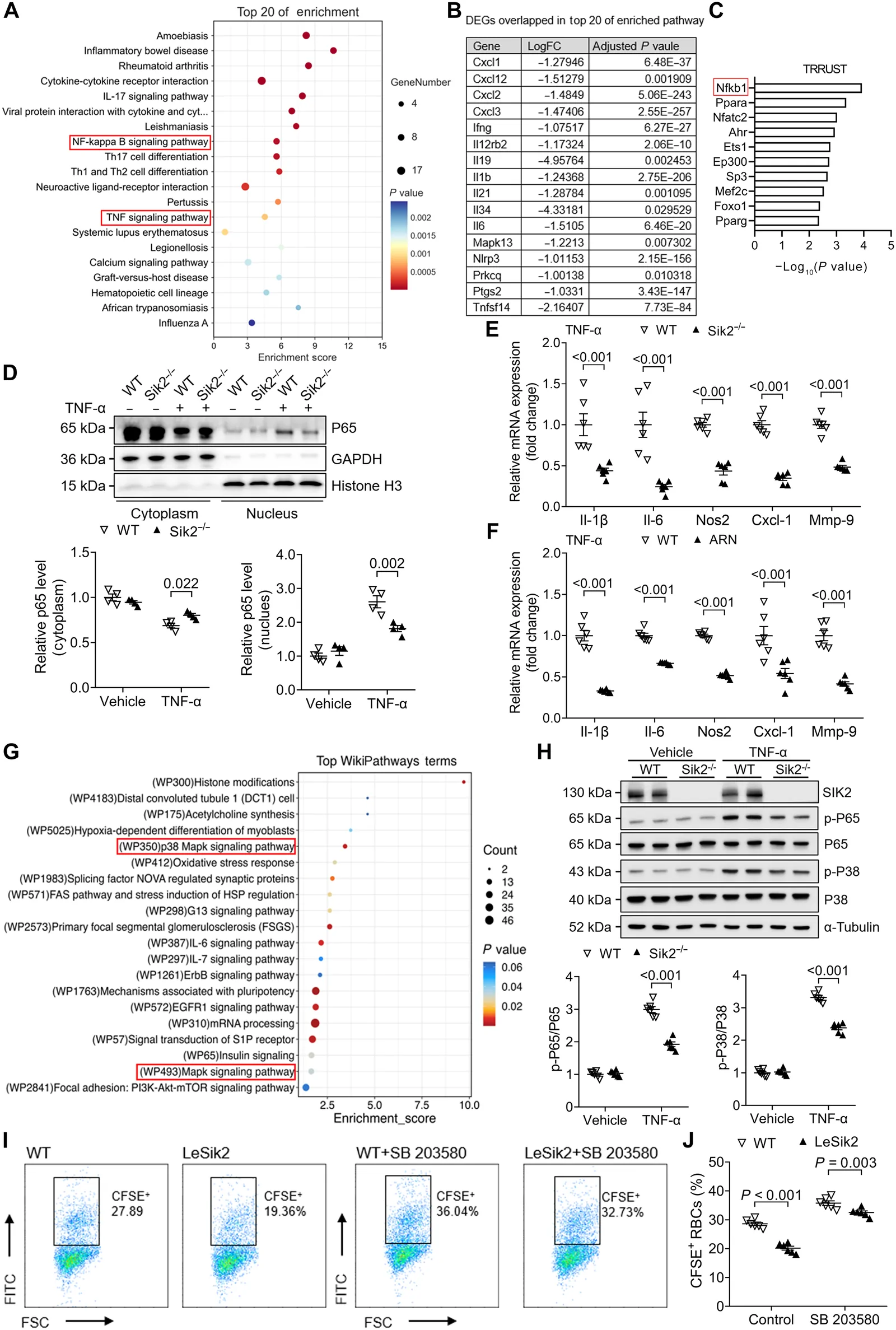
SIK2 depletion reduces inflammatory response and improves efferocytosis in macrophages via inhibiting P65 and P38 signaling pathways. (**A**) KEGG enrichment analysis of down-regulated DEGs in Sik2^F/F^; LysM^Cre^ cardiac macrophages on day 3 post-MI. The top 20 enriched pathways are listed. (**B**) DEGs (fold change ≥ 2 and *P*.adjust ≤ 0.05) overlapped in top 20 of enriched pathways. (**C**) Transcription factor prediction was performed using TRRUST databases. The top 10 transcription factors are listed. (**D**) Immunoblotting analysis and quantification of P65 in the cytoplasm and nucleus of WT and SIK2-knockout PMs and treated with TNF-α (20 ng/ml) or vehicle (*n* = 4) for 1 hour. (**E**) qPCR analysis of *Il-1β*, *Il-6*, *Nos2*, *Cxcl-1*, and *Mmp-9* mRNA expression in WT and SIK2-knockout PMs stimulated with TNF-α for 24 hours (*n* = 6). (**F**) qPCR analysis of *Il-1β*, *Il-6*, *Nos2*, *Cxcl-1*, and *Mmp-9* mRNA expression (*n* = 6). PMs were pretreated with ARN3236 (3 μmol) and stimulated with TNF-α for 24 hours. (**G**) WikiPathways enrichment analysis of differentially phosphorylated proteins in PMs treated with ARN3236 (3 μmol) or control. The top 20 enriched pathways are listed. (**H**) Immunoblotting analysis and quantification of phosphorylated P65 (p-P65), P65, phosphorylated P38 (p-P38), and P38 expression in WT and SIK2-knockout PMs stimulated with TNF-α or vehicle (*n* = 6). (**I** and **J**) Flow cytometry analysis of efferocytosis in WT or LeSik2 Raw264.7 cells [stimulated with SB 203580 (10 μmol) or not] treated with CFSE-prelabeled RBCs (*n* = 6).

The SIK2-P65-NF-κB pathway partially explained the enhanced inflammatory response of macrophages but failed to explain the impaired efferocytosis. Therefore, we further explored changes in SIK2 as a kinase in the downstream phosphorylation pathway. Quantitative phosphorylation proteomic profiling was performed on PMs treated with ARN3236 and control (fig. S11A). Phosphoproteomic analysis of macrophages identified 10,539 phosphorylation sites, of which 9330 phosphorylation sites were quantified, corresponding to 3109 phosphorylated proteins; 1197 sites showed significant differences between ARN-treated and control macrophages. Among these, 399 and 798 phosphorylation sites were up-regulated and down-regulated, respectively (fig. S11B). WikiPathways analysis showed the top 20 altered enriched pathways, including P38 and mitogen-activated protein kinase (MAPK) pathways (Fig. 5G). The key protein of the MAPK pathway, Mapk14 (referred to as P38), was enriched in the protein-protein interaction network (fig. S11C). Western blot analysis confirmed decreased phosphorylation of P65 and P38 in SIK2-cKO PMs (Fig. 5H). Next, we investigated whether the SIK2-P38-MAPK pathway could explain the impaired efferocytosis. Flow cytometry confirmed impaired efferocytosis in the LeSik2 macrophages. However, treatment with a selective P38 inhibitor (SB 203580) increased the phagocytic activity of CFSE^+^ RBCs (Fig. 5, I and J). A previous study has shown that P38-MAPK is a key player in the subsequent engulfment step, partly by controlling the transcription of essential receptors (*30*). We found that the SIK2-driven suppression of phagocytic receptors, including *Cd36*, *Mertk*, *Timd4*, *Cx3cr1*, and *Mfge8*, could be rescued by P38 inhibition (fig. S12A). Notably, LeSik2 macrophages showed increased phosphorylation of P65 and NOS2, which was partially suppressed by SB 203580 (fig. S13A). A previous study has shown that mutations at Thr^175^ of SIK2 result in its inactivation (*31*). To further investigate whether the inactivated form of SIK2 regulates the activation of P38-MAPK and P65-NF-κB, we performed Western blot analysis. The results demonstrated that the SIK2 Thr^175^ mutation [Thr^175^→Ala (T175A); SIK2^T175A^] failed to induce phosphorylation of P38 and P65 (fig. S13B). These findings show that SIK2 induces a macrophage inflammatory response and defective efferocytosis via P38-MAPK and P65-NF-κB signaling, depending on the Thr^175^ site.

### SIK2 interacts with TRAF2 and regulates its K63-linked polyubiquitination

To further investigate the mechanism by which SIK2 regulates the P38-MAPK and P65-NF-κB signaling pathways, we identified potential proteins that interact with SIK2 using coimmunoprecipitation followed by mass spectrometry. Mass spectrometry was used to identify SIK2-interacting candidate proteins, which were then subjected to Venn diagram analysis. The total proteins associated with the two enrichment pathways were highlighted by transcriptomic and phosphoproteomic analyses (Fig. 6A). Notably, tumor necrosis factor (TNF) receptor–associated factor 2 (TRAF2) emerged as the top candidate within the intersecting set (Fig. 6B and fig. S14A). Moreover, transcriptomic analysis revealed that the mRNA levels of the three interacting protein candidates within the intersecting set were not significantly altered in SIK2-cKO cardiac macrophages (table S4). Therefore, we sought to validate TRAF2 as a bona fide substrate for SIK2. Reciprocal coimmunoprecipitation experiments confirmed an interaction between SIK2 and TRAF2 (Fig. 6, C to E). Concurrently, we confirmed an endogenous interaction between SIK2 and TRAF2 in macrophages (fig. S14B). Crucially, under the same stringent conditions, we did not detect any specific interaction between SIK2 and other major TRAFs, including TRAF1, TRAF4, and TRAF6 (fig. S15A). To further delineate the specific domain of SIK2 that interacts with TRAF2, truncated SIK2 was coimmunoprecipitated with TRAF2. The ubiquitin-associated (UBA) domain (amino acids 295 to 335 of SIK2) was identified as essential for its interaction with TRAF2 (Fig. 6F).

**Fig. 6.**
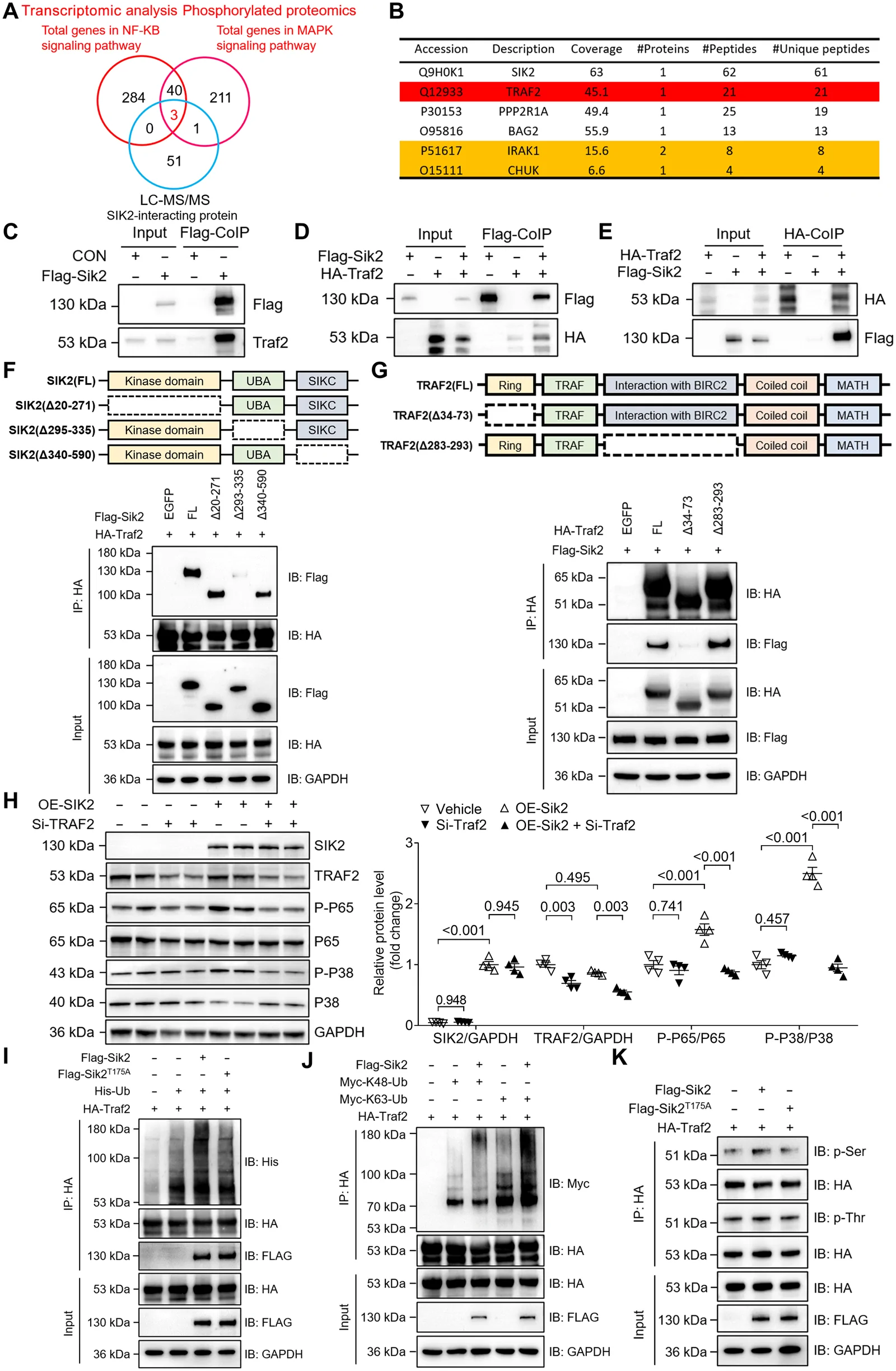
SIK2 mediates TRAF2 K63-linked ubiquitylation through serine phosphorylation to regulate P38 and P65 signaling pathways. (**A**) Venn diagram delineating the intersection of SIK2-binding protein candidates in the top enriched pathways identified in Fig. 5 (A and G), that is, NF-κB signaling pathway and MAPK signaling pathway. LC-MS/MS, liquid chromatography–tandem mass spectrometry. (**B**) Top proteins interacting with SIK2 identified through mass spectrometry analysis of peptides pulled down using anti-Flag antibody in HEK293T cells transfected with Flag-Sik2. (**C**) Immunoprecipitation in HEK293T cells transfected with Flag-Sik2 using anti-Flag antibody, followed by immunoblotting with corresponding antibodies. HA, hemagglutinin. (**D** and **E**) Immunoprecipitation in HEK293T cells transfected with Flag-Sik2 and HA-Traf2 using anti-Flag (D) or anti-HA (E) antibody, followed by immunoblotting with corresponding antibodies. FL, full length. (**F**) Immunoprecipitation (IP) in HEK293T cells cotransfected with HA-Traf2 and truncated Flag-Sik2 using an anti-HA antibody, followed by immunoblotting (IB) with corresponding antibodies. (**G**) Immunoprecipitation in HEK293T cells cotransfected with Flag-Sik2 and truncated HA-Traf2 using an anti-HA antibody, followed by immunoblotting with corresponding antibodies. (**H**) Western blotting analysis and quantification of SIK2, TRAF2, p-P38, P38, p-P65, and P65 expression in HEK293T cells transfected with SIK2 and si-TRAF2 or control (*n* = 4). (**I**) Coimmunoprecipitation (Co-IP) analysis of TRAF2 ubiquitination in HEK293T cells cotransfected with HA-Traf2 and His-Ub, along with Flag-Sik2 or Flag-Sik2^T175A^ mutant. (**J**) Co-IP analysis of TRAF2 ubiquitination in HEK293T cells cotransfected with HA-Traf2 and Flag-Sik2, along with Myc-K48-Ub or Myc-K63-Ub. (**K**) Co-IP analysis of TRAF2 phosphate serine (p-Ser) and phosphate threonine (p-Thr) in HEK293T cells transfected with HA-Traf2, along with Flag-Sik2 or Flag-Sik2^T175A^ mutant.

TRAF2 is an important connector molecule in the TNF signaling complex that facilitates downstream signaling cascades, including activation of the P38-MAPK and P65-NF-κB pathways (*32*). A previous study has reported that K63-linked polyubiquitin chains can attach to TRAF2, serving as a scaffold for triggering P38-MAPK and P65-NF-κB signaling cascades (*33*). The Ring-type zinc finger domain is essential for E3 ubiquitin–protein ligase activity (*34*). In addition, the c-IAP1 can bind to TRAF2 and mediate its ubiquitination (*35*). Further investigation revealed that only Ring domain-truncated TRAF2 (Δ34-73) failed to interact with SIK2, whereas full-length TRAF2 and other truncated TRAF2 could bind with SIK2 (Fig. 6G). Notably, SIK2 overexpression increased the phosphorylation of P38 and P65, whereas TRAF2 knockdown inhibited these effects (Fig. 6H). These results suggested that SIK2 activates the P38-MAPK and P65-NF-κB pathways in a TRAF2-dependent manner. Next, we transfected human embryonic kidney (HEK) 293T cells with SIK2, TRAF2, and ubiquitin. Overexpression of SIK2 increased the polyubiquitination level of TRAF2 but not that of SIK2^T175A^ (Fig. 6I). In addition, the kinase activation site at Thr^175^ is not required for the binding of SIK2 to TRAF2 (Fig. 6I). We then analyzed the ubiquitination status of TRAF2 in cardiac macrophages from post-MI mice that were either SIK2-cKO, treated with ARN3236, or WT controls. We observed a notable reduction in TRAF2 ubiquitination in macrophages from both SIK2-cKO and ARN3236-treated mice compared to WT controls (fig. S15B). Inactive SIK2 mutant (SIK2^T175A^) failed to promote TRAF2 ubiquitination, indicating that phosphorylation of TRAF2 by SIK2 is essential for its subsequent ubiquitination. Furthermore, a TRAF2 mutant harboring phosphoresistant substitutions at residues S11/S55/T115 (*36*, *37*) also abrogated SIK2-induced ubiquitination (fig. S15C). This suggests that phosphorylation at these specific sites is critical for TRAF2’s ability to be ubiquitinated in response to SIK2 signaling. K48-linked polyubiquitination primarily mediates proteasomal degradation of substrate proteins, whereas K63-linked ubiquitin chains mediate signal transduction via nondegradative mechanisms (*38*). Therefore, we transfected HEK293T cells with SIK2, TRAF2, K48-specific ubiquitin, or K63-specific ubiquitin. We observed that K63-linked ubiquitination of TRAF2 was substantially more abundant than K48-linked ubiquitination (Fig. 6J). Considering that the phosphorylation of TRAF2 can promote K63-linked ubiquitination of TRAF2 (*37*), whereas SIK2^T175A^ failed to promote TRAF2 polyubiquitination, we further immunoprecipitated TRAF2 in HEK293T cells and evaluated the TRAF2 phosphorylation levels of serine (Ser) and Thr upon transfection with SIK2 or SIK2^T175A^. Our results showed that SIK2, but not SIK2^T175A^, substantially increased the p-Ser level of TRAF2, with no appreciable change in the p-Thr of TRAF2 (Fig. 6K). To pinpoint the critical phosphorylation site, we performed site-directed mutagenesis of TRAF2, substituting Ser^11^/Ser^55^ with alanine (S11A/S55A) to abolish potential phosphorylation at this residue. Notably, when coexpressed with SIK2, the TRAF2-S11A mutant exhibited no increase in Ser phosphorylation compared to WT TRAF2. In contrast, mutation of Ser^55^ alone (S55A) did not fully block SIK2-induced phosphorylation (fig. S15D). Collectively, these findings indicate strong binding between the SIK2 UBA domain and the TRAF2 Ring domain, leading to Ser^11^ phosphorylation of TRAF2 and promotion of TRAF2 K63-linked polyubiquitination.

### Inhibition of P38 activation rescues the detrimental effects induced by SIK2 overexpression in MI

To evaluate whether macrophage SIK2 overexpression aggravates cardiac injury following MI, WT mice were injected with an adeno-associated virus (AAV) expressing SIK2 or SIK2^T175A^, driven by a macrophage-specific F4/80 promoter (AAV-F4/80-SIK2 or AAV-F4/80-SIK2^T175A^), as previously described (*39*). Furthermore, the effect of P38 blockade (SB 203580) was investigated (Fig. 7A). SIK2 overexpression in infarcted macrophages was confirmed using immunoblotting (Fig. 7B). Echocardiography indicated that SIK2 overexpression significantly reduced EF and FS, accompanied by an increase in the left ventricular volume and internal diameter (table S5). Pharmacological inhibition of P38 restored the impaired cardiac function in SIK2-overexpressing mice post-MI (Fig. 7C). Consistently, SIK2 overexpression in macrophages increased infarct size compared to that in the control group. However, infarct size was reduced in both the control and SIK2 overexpression groups treated with the P38 inhibitor (Fig. 7D). The expression of key inflammation-associated genes was assessed in cardiac macrophages. The results showed that SIK2 overexpression promoted a proinflammatory gene signature (*Il-1α*, *Il-1β*, *Il-6*, *Nos2*, *Cxcl-1*, *Tnf-α*, and *Mmp-9*) while suppressing reparative (*Arg1*, *Il-10*, *Tgfb1*, and *Vegfa*) and efferocytosis receptor (*Cd36* and *Cx3cr1*) genes. Notably, SB 203580 treatment reversed nearly all these transcriptional changes (fig. S16A). Furthermore, Prussian blue staining revealed reduced iron deposition in the AAV-SIK2 group, indicative of impaired phagocytosis. Critically, this deficit was fully rescued in mice treated with the P38 inhibitor (fig. S17A). Consistent with the results of SIK2^T175A^ overexpression in macrophages in vitro, echocardiography showed no significant differences in EF and FS between SIK2^T175A^ overexpression mice and control mice after MI (table S6), as well as in the left ventricular volume and internal diameter (Fig. 7, E to G). Similarly, no significant difference in infarct size was observed between the two groups after MI (Fig. 7H). These in vivo experiments provide further evidence that the deleterious effects of macrophage SIK2 on MI are dependent on its kinase activity and that this effect can be inhibited by blocking P38.

**Fig. 7.**
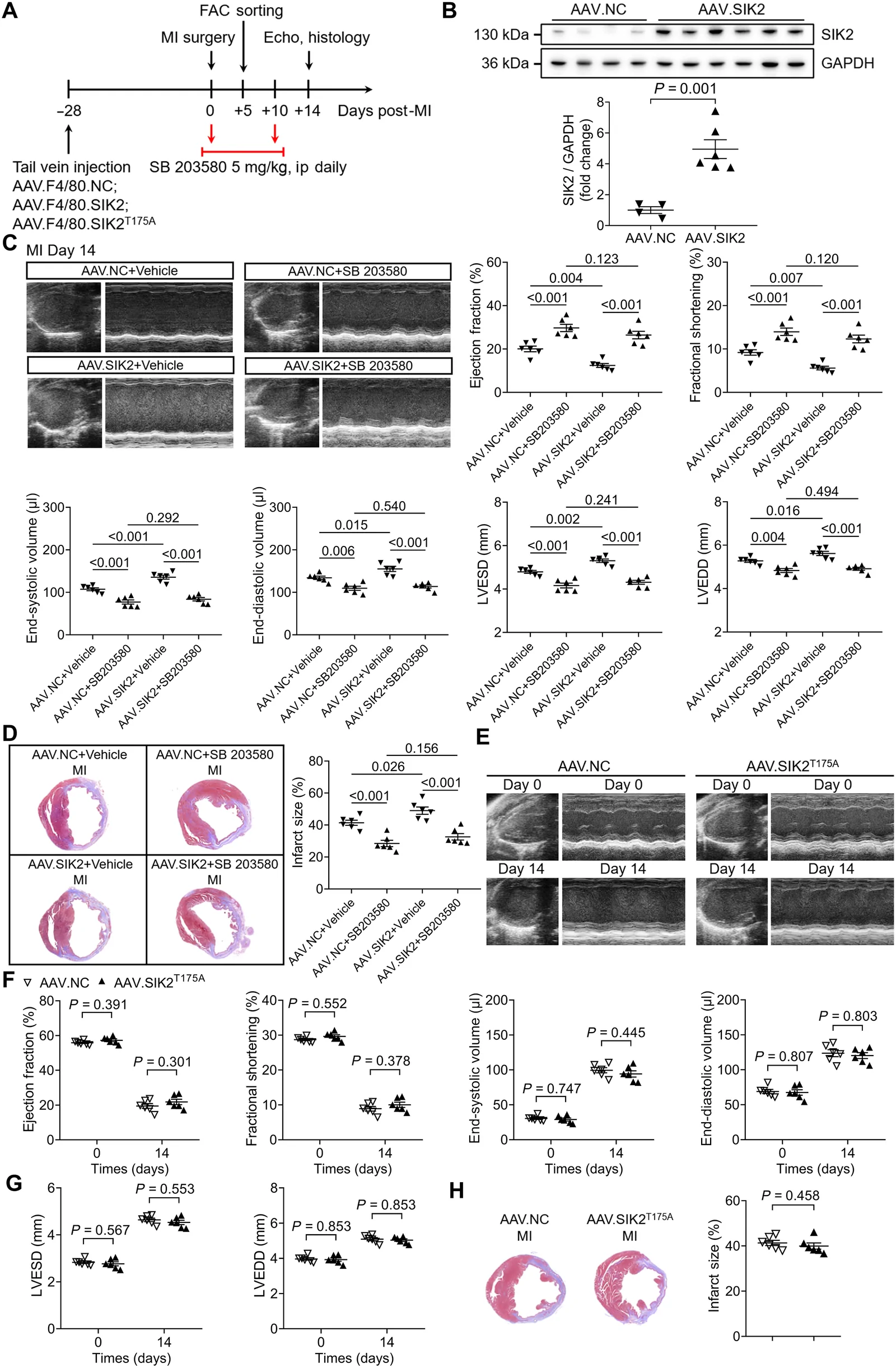
Overexpression of SIK2 but not SIK2^T175A^ in macrophages exacerbates post-MI cardiac injury and can be ameliorated by pharmacological inhibition of P38. (**A**) Experimental design depicting AAV and P38 inhibitor SB 203580 injection in mice before or after MI. ip, intraperitoneally. (**B**) Immunoblotting analysis and quantification of SIK2 in infarct macrophages from AAV.F4/80.SIK2 or AAV.F4/80.NC injected mice (*n* = 4 for AAV.F4/80.NC; *n* = 6 for AAV.F4/80.SIK2). (**C**) Representative parasternal long-axis views and M-mode images are shown. Summarized data of echocardiographic measurements in WT mice administrated with AAV.F4/80.SIK2 or AAV.F4/80.NC treated with SB 203580 or vehicle (*n* = 6). (**D**) Masson’s trichrome staining for infarct size measurements in designated groups at day 14 after MI (*n* = 6). (**E**) Representative parasternal long-axis views and M-mode images are shown. (**F** and **G**) Summarized data of echocardiographic measurements in WT mice administrated with AAV.F4/80.SIK2^T175A^ or AAV.F4/80.NC (*n* = 6). (**H**) Masson’s trichrome staining for infarct measurements in designated groups at day 14 after MI (*n* = 6).

### Pharmacological blockade of SIK2 improves tissue repair and cardiac function after MI

The therapeutic potential of targeting SIK2 for the treatment of MI was explored in vivo (Fig. 8A). We administered the SIK2 inhibitor ARN3236 to MI model mice and found that ARN3236 enhanced cardiac function in vivo, as evidenced by an increase in EF and FS (table S7). However, it did not affect cardiac function in sham-operated mice. In addition, ARN3236 improved adverse cardiac remodeling by reducing left ventricular volume and left ventricular internal diameter after MI (Fig. 8, B to D). Furthermore, the post-MI infarct size was reduced in mice treated with ARN3236 (Fig. 8E). Prussian blue staining revealed greater iron deposition in the SIK2 inhibitor-treated infarcted area, confirming increased debris clearance (Fig. 8F). Flow cytometry analysis of cardiac macrophages (CD45^+^CD11b^+^F4/80^+^) identified a higher percentage of cells containing cardiomyocytes in SIK2 inhibitor-treated hearts, quantifying enhanced efferocytosis (Fig. 8G). Consistent with the findings in SIK2-cKO mice, the expression levels of several genes associated with the proinflammatory macrophage phenotype were significantly reduced. In contrast, those of several genes associated with the reparative macrophage phenotype, cardiac healing, and angiogenesis were markedly elevated (fig. S18, A and B). Collectively, these findings suggest that the pharmacological inhibition of SIK2 can improve cardiac function and promote cardiac repair after MI, highlighting the potential therapeutic benefit of targeting SIK2 in MI treatment.

**Fig. 8.**
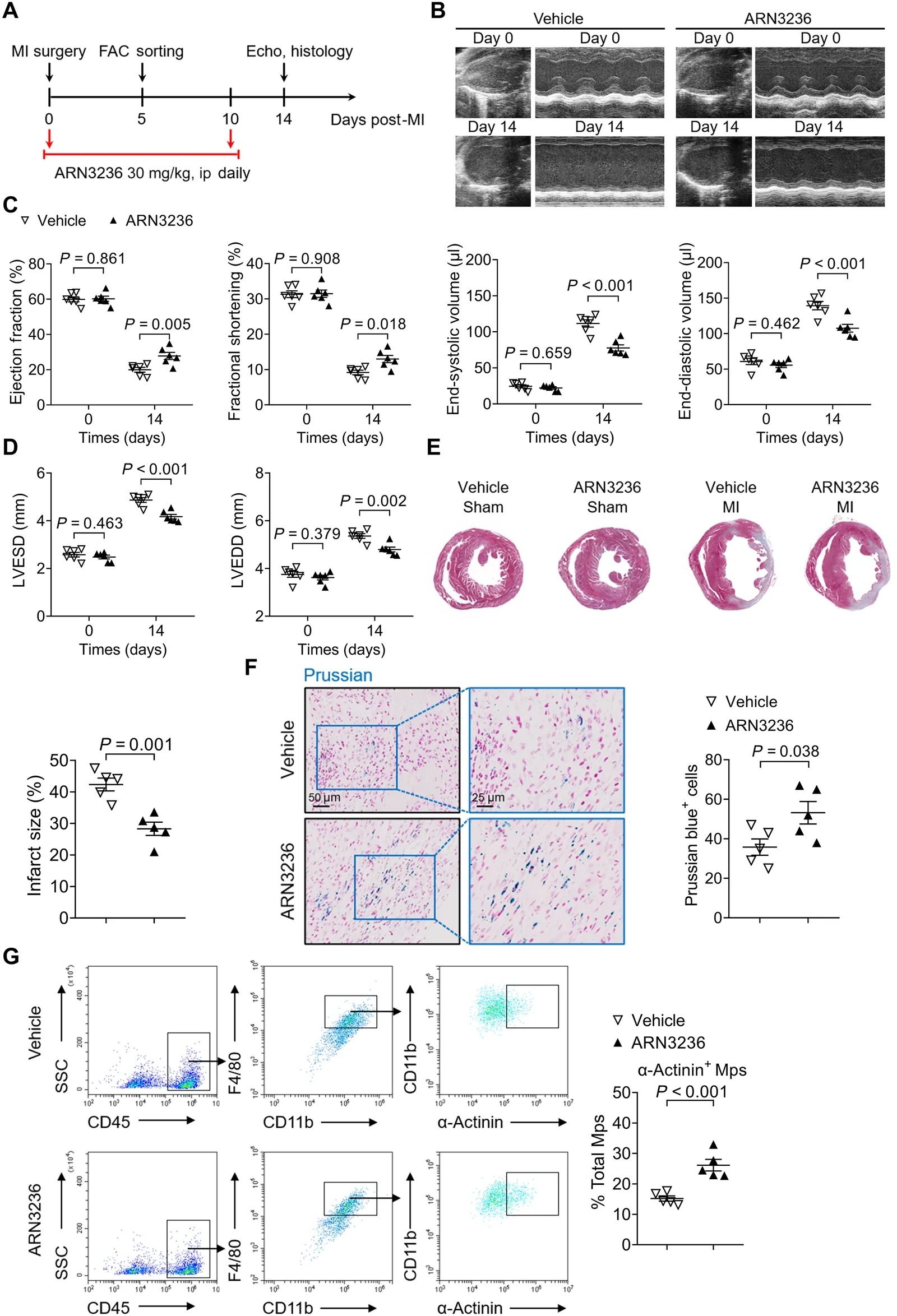
Pharmacological blockade of SIK2 improves tissue repair and cardiac function post-MI. (**A**) Design of the ARN3236 injection in mice post-MI. (**B**) Representative parasternal long-axis views and M-mode images. (**C** and **D**) Echocardiographic analysis of EF, FS, LVESV, LVEDV, LVESD, and LVEDD on days 0 and 14 after MI or sham operation in ARN3236-treated or vehicle mice (*n* = 6). (**E**) Masson’s trichrome staining for infarct size measurements in ARN3236-treated or vehicle mice at day 14 after MI or sham operation (*n* = 5). (**F**) Representative images and related quantification of Prussian blue staining assay for the phagocytosis of erythrocyte and cellular debris in macrophages in hearts of ARN3236-treated or vehicle mice (*n* = 5). (**G**) Gating strategy for flow cytometry analysis of CD45^+^CD11b^+^F4/80^+^α-Actinin^+^ macrophages isolated from ARN3236-treated or vehicle infarcts 3 days post-MI (*n* = 5).

### Partial TRAF2 deficiency improves myocardial function after MI

To elucidate the in vivo role of TRAF2 in MI, we generated TRAF2 heterozygous (Traf2^+/−^) mice (fig. S19A), as germline deletion of TRAF2 is embryonically lethal (*40*). We subjected Traf2^+/−^ and WT littermates to MI. Our results demonstrate that haploinsufficiency of TRAF2 confers cardioprotection (table S8). Compared to WT controls, Traf2^+/−^ mice exhibited markedly improved post-MI cardiac function, as evidenced by significantly increased EF and FS values. Crucially, this functional improvement was specific to the MI setting as no differences in EF or FS were observed between genotypes in the sham-operated group. Beyond functional benefits, Traf2^+/−^ mice displayed attenuated adverse cardiac remodeling. This remodeling attenuation was characterized by reduced left ventricular volumes and internal dimensions (fig. S19, B and C). Furthermore, infarct size was smaller in Traf2^+/−^ mice 2 weeks post-MI, as confirmed by Masson’s trichrome staining (fig. S19D). Prussian blue staining revealed a notable increase in phagocytic remnants within the infarct zone of Traf2^+/−^ hearts (fig. S19E). This was accompanied by a shift toward a reparative gene expression profile. qPCR analysis showed down-regulation of proinflammatory cytokines and up-regulation of pro-reparative factors in the infarcted myocardium of Traf2^+/−^ mice (fig. S20A).

## DISCUSSION

Macrophages play crucial roles as effectors and regulators of the inflammatory cascade that triggered by MI. Our results indicated that macrophage SIK2 promoted a proinflammatory state and impaired efferocytosis in the post-MI hearts. Specifically, SIK2 increased the number of proinflammatory Ly6C^high^ macrophages and hindered efferocytosis during MI. Furthermore, SIK2 kinase activation induced TRAF2 serine phosphorylation, which promoted TRAF2 K63 ubiquitylation. SIK2/TRAF2 axis facilitates P38-MAPK and P65-NF-κB signaling activation, increases the TNF-associated immune response, and inhibits efferocytotic capacity, thus contributing to cardiac dysfunction and remodeling. Collectively, these findings reveal a hitherto underexplored mechanism by which SIK2 activation maintains an inflammatory state while hindering efferocytosis in macrophages and impairing post-MI cardiac repair.

Excessive inflammation and inadequate repair are associated with poor outcomes following MI (*41*). Insufficient infarct healing causes gradual thinning and dilation of the infarct wall, leading to scar malformation, increased risk of cardiac rupture, and adverse cardiac remodeling. Notably, we found that SIK2-cKO mice had significantly improved cardiac function, reduced infarct size, and lower mortality from cardiac rupture following MI owing to enhanced infarct healing. An excess of leukocytes, particularly Ly6C^high^ macrophages and neutrophils, in infarcts worsens the inflammatory injury (*42*). In contrast, the timely transition of the macrophage population from a proinflammatory to a pro-resolving state supports infarct healing (*43*). Our findings demonstrated that macrophages exhibit elevated SIK2 expression during the acute phase following MI, suggesting that SIK2 may contribute to the activation of the proinflammatory macrophage phenotype. In addition, SIK2-cKO infarcts had fewer infiltrating inflammatory neutrophils and Ly6C^high^ macrophages but contained a higher abundance of reparative Ly6C^low^ macrophages, leading to the timely resolution of inflammation.

SIK2 has been shown to regulate energy balance and metabolic processes (*44*). Our study revealed that SIK2 functions as a key regulator of the macrophage inflammatory response. SIK2-deficient infarcted macrophages exhibit reduced expression of *Il-1α*, *Il-1β*, *Nos2*, *Il-6*, and *Mmp9*, all known factors of postinfarction inflammatory damage and protease-driven adverse remodeling (*45*). Conversely, these macrophages exhibited increased expression of *Vegfa*, *Arg1*, *Tgfb1*, and *Il-10*, which are known to promote CF differentiation, collagen synthesis, and angiogenesis during post-MI repair (*46*). Consistent with these findings, our transcriptomic profiling analysis revealed that several signaling pathways linked to the inflammatory response, as well as a broad range of genes involved in amplifying the inflammatory response and leukocyte recruitment, were significantly suppressed in SIK2-cKO cardiac macrophages. Excessive inflammation exacerbates myocardial apoptosis, impairs efferocytosis, and worsens ischemic injury (*47*, *48*). In addition, we found that SIK2 deficiency in macrophages protects against ischemic cardiac injury by reducing apoptosis in the border area after MI. Efferocytosis of apoptotic cells is essential for preventing unchecked inflammation (*49*). Phagocytes release pro-reparative cytokines such as VEGFA, TGFβ1, and IL-10 upon recognition of apoptotic cells, thereby improving cardiac repair after MI (*19*). Our results showed that SIK2-cKO macrophages help prevent the accumulation of apoptotic cardiomyocytes, protect against excessive leakage of apoptotic cell contents, and facilitate proper resolution of inflammation. In addition, reparative mediators produced by efferocytosis promote fibroblast differentiation, thereby improving cardiac repair. Future studies should explore how SIK2 influences the function of other macrophages during MI.

An important finding is that SIK2 regulates inherent proinflammatory activation and efferocytosis of macrophages by modulating TRAF2 polyubiquitination. The dual functional role of macrophages in MI, including the initiation and resolution of inflammation, requires a mechanism to program the macrophage phenotype. This process necessitates the coordination of several critical signaling pathways, especially the P65-NF-κB signaling pathway. The activation and nuclear translocation of P65-NF-κB lead to the up-regulation of various proinflammatory cytokines, chemokines, and adhesion molecules in infarcted macrophages, therefore exacerbating cardiac damage (*8*). Similarly, activation of P38-MAPK signaling impairs efferocytosis (*30*), resulting in the accumulation of apoptotic cardiomyocytes and further triggering the inflammatory response (*28*). TNF receptor signaling, activated by membrane-bound TNF, facilitates the recruitment of TRAF2, which, in turn, promotes downstream signaling cascades, including the activation of P38-MAPK and P65-NF-κB (*50*). Our data demonstrated that SIK2 amplified the inflammatory cytokine expression and hindered the efferocytotic capacity of macrophages through TRAF2 K63 ubiquitylation. TRAF2 phosphorylation has been reported to determine K63-linked polyubiquitination at lysine-31 (*36*). We found that SIK2 promoted the phosphorylation of TRAF2 through its kinase activity, triggering subsequent K63 ubiquitination to mediate the downstream signaling cascade. Notably, this kinase-mediated control of TRAF2 ubiquitination appears to represent a common regulatory mechanism, as recent studies have shown that two other kinases, PKC (protein kinase C) and IKKε (inhibitor of NF-κB subunit ε), govern TNF signaling in tumor cells (*36*, *37*). Therefore, SIK2 regulates a proinflammatory phenotype and impaired efferocytosis in infarcted macrophages, at least in part, by enhancing P38-MAPK and P65-NF-κB pathway activity through the promotion of TRAF2 K63 ubiquitylation. SIK2 has been reported to limit the formation of regulatory macrophages and the production of anti-inflammatory molecules, such as IL-10, by inhibiting the dephosphorylation of cAMP response element–binding protein (CREB)–regulated transcriptional coactivator (CRTC3), which enhances the gene transcription program controlled by CREB (*10*). However, CRTC3 was not identified among the candidate proteins that interact with SIK2 in our coimmunoprecipitation mass spectrometry analysis.

This study primarily investigated the role and mechanism of SIK2 in cardiac injury, along with an early intervention strategy targeting SIK2 in a murine MI model. Notably, high SIK2 expression was observed in PBMC samples from patients with MI. A recent study has highlighted the therapeutic potential of pro-resolving agents in addressing uncontrolled inflammation (*51*). Pharmacological blockade of SIK2 improved cardiac function in murine MI models. This finding would certainly have immediate therapeutic significance for treating patients with acute MI.

Our study has several limitations. Owing to the lack of human MI tissue and cell samples, current studies are limited to animal models. Further investigation of SIK2 expression in the hearts of patients with MI is required. Similarly, LysM-Cre–driven gene deletion affected both neutrophils and monocytes/macrophages. Our study showed that SIK2 is mainly localized in macrophages but not in neutrophils. However, the function of SIK2 in neutrophils requires further investigation.

In summary, our mechanistic and functional studies showed that SIK2 is a critical determinant of inflammatory signaling in macrophages through its phosphorylation and ubiquitination effects on TRAF2, thereby aggravating myocardial inflammation and impairing efferocytotic capacity in a manner dependent on both the P38-MAPK and P65-NF-κB signaling after MI. Consequently, targeting macrophage SIK2 may represent an innovative and promising therapeutic strategy for MI treatment. Our findings provide valuable insights into the mechanisms governing the post-MI inflammatory balance.

## MATERIALS AND METHODS

### Human samples

This study was approved by the Ethics Committee of Shanghai General Hospital, Shanghai Jiao Tong University School of Medicine and was performed in accordance with the principles of the Declaration of Helsinki. Informed consent was obtained from all participants. Peripheral venous blood samples were collected from patients clinically diagnosed with acute MI and from those without cardiovascular disease. Human PBMCs were isolated from the peripheral venous blood using a mononuclear cell separation solution (P9011, Solarbio, Beijing, China).

### Mice

All animal experiments were performed according to a protocol approved by the Institutional Animal Care and Use Committee of Cyagen Biomodel Limited Liability Company (license number: TACU22-FY723). Male WT C57BL/6 mice, aged 8 to 10 weeks, were obtained from Cyagen Biosciences Inc. (Suzhou, Jiangsu, China). Sik2 flox (Sik2^F/F^) and LysM-Cre mice with a C57BL/6 background were purchased from Cyagen Biosciences Inc. Sik2^F/F^ mice were bred with LysM-Cre mice to generate macrophage-specific SIK2 knockout mice. Littermate Sik2^F/F^ mice were used as controls. Traf2^+/−^ mice with a C57BL/6 background were purchased from Cyagen Biosciences Inc.

#### Transthoracic echocardiography

Cardiac function was evaluated at various time points following surgery using a Vevo 2100 ultrasound system (VisualSonics, Toronto, Canada). The mice were lightly anesthetized with 1% isoflurane and positioned on a heated echocardiography platform. The left ventricle and aortic outflow tract were visualized using two-dimensional B-mode imaging, with the sample line positioned at the largest cross section of the left ventricle to capture serial M-mode echocardiographic images. Measurements of LVESV, LVEDV, EF, FS, LVESD, and LVEDD were taken from a minimum of three separate frames for each mouse.

#### Histological analysis

Heart specimens were perfused with chilled phosphate-buffered saline (PBS) to remove blood, fixed in formalin, embedded in paraffin, and sectioned into 5-μm-thick slices. Tissue sections were stained using Picrosirius red and Masson’s trichrome to assess morphological changes and measure infarct size. The infarct size was quantified as a percentage of the necrotic area relative to the total left ventricular area as described previously (*24*). Collagen volume fraction was assessed by analyzing five randomly selected high-power fields (200× magnification) in each tissue section. Tissue sections were stained with Prussian blue to detect iron particles within macrophages. Phagocytic activity was assessed by quantifying the number of Prussian blue–positive macrophages within high-power fields in the infarcted region of the heart. The measurements and calculations were performed using the ImageJ software (National Institutes of Health, Bethesda, MD, USA).

#### Immunofluorescence staining

For immunofluorescence analysis, hearts were harvested and embedded in optimal cutting temperature (OCT) compound (Sakura, Torrance, CA, USA). Cryosections with a thickness of 7 μm were prepared, fixed, and blocked with diluted donkey serum for 1 hour at room temperature (25°C), followed by overnight incubation with the primary antibodies at 4°C. The signals were detected using Alexa Fluor–conjugated secondary antibodies (Invitrogen, Carlsbad, CA, USA). Primary antibodies against the following targets were used in these experiments: SIK2 (6919s, 1:200; CST, Danvers, MA, USA), Ly6G (551459, 1:200; BD Biosciences, Franklin Lakes, NJ, USA), CD68 (ab53444, 1:200; Abcam, Cambridge, UK), TNNI3 (SAB2502170, 1:200; Sigma-Aldrich, St. Louis, MO, USA), α-SMA (A5228, 1:700; Sigma-Aldrich), Collagen I (NBP1-30054, 1:200; Novus Biologicals, Littleton, CO, USA), CD31 (ab222783, 1:200; Abcam), NOS2 (13120s, 1:200; CST), and Arginase-1 (93668s, 1:200; CST). Images were acquired using a laser scanning microscope (Axio Imager M2, Carl Zeiss, Jena, Germany).

#### Isolation of mouse PMs

Macrophage elicitation in male mice aged 6 to 8 weeks was induced through intraperitoneal injection of 3% Brewer’s thioglycolic acid. Macrophages were cultured in RPMI 1640 medium (Gibco, Grand Island, NY, USA) with penicillin (100 U/ml), streptomycin (0.1 mg/ml; V900929, Sigma-Aldrich), and 10% fetal bovine serum (FBS). After incubating for 2 to 3 hours, the cells were washed twice with Hepes buffer to eliminate any contaminating cell types. Macrophages were incubated overnight at 37°C and 5% CO_2_. TNF-α (20 ng/ml) was added for PM differentiation into proinflammatory type. Subsequently, the cells were harvested for phenotypic characterization and functional assays at the specified time points. For the SIK2 inhibitor group, macrophages were incubated with the SIK2 inhibitor ARN3236 (3 μM) and TNF-α (20 ng/ml) for 24 hours.

#### TUNEL staining

For the analysis of cardiomyocyte apoptosis, 7-μm-thick frozen heart sections were fixed with 4% paraformaldehyde for 15 min, permeabilized with 0.1% Triton X-100 in PBS for 10 min, and then subjected to TUNEL staining using an In Situ Apoptosis Detection kit (Yeasen, Shanghai, China). Following this, the sections were costained with anti-TNNI3 antibody to specifically label cardiomyocytes. For each cross section, six to eight randomly selected fields from the infarct, border, and remote areas of MI hearts were examined using a microscope (Carl Zeiss, Germany). The apoptotic index was determined by calculating the percentage of apoptotic cardiomyocytes (TUNEL/TNNI3 double-positive) relative to the total cardiomyocyte count. Representative images for each group were selected on the basis of the average value.

#### Cell transfection

HEK293T cells were generously provided by the Cell Bank of the Chinese Academy of Sciences (CAS) and cultured in Dulbecco’s modified Eagle’s medium (DMEM) supplemented with 10% FBS at 37°C in a 5% CO_2_ humidified atmosphere. Plasmid and small interfering RNA (siRNA) transfections into HEK293T cells were performed using Lipofectamine 3000 (L3000015, Invitrogen) according to the manufacturer’s instructions. The plasmids used in this study included constructs encoding Flag-Sik2, Flag-Sik2^T175A^ (threonine mutants), Flag-Sik2 (Δ20-271; kinase domain truncated), Flag-Sik2 (Δ295-335; UBA domain truncated), Flag-Sik2 (Δ340-590; C-terminal domain truncated), HA-Traf2, HA-Traf2 (Δ34-73; Ring-type zinc finger truncated), HA-Traf2 (Δ283-293; BIRC2-interacting domain truncated), His-ubiquitin, Myc-K48-ubiquitin (all lysine residues were mutated to arginine, except for K48), Myc-K63-ubiquitin (all lysine residues were mutated to arginine, except for K63), as well as the corresponding empty vector. All plasmids were constructed by GeneChem Co. Ltd. (Shanghai, China). Si-TRAF2 was constructed by Bioscience Co. Ltd. (Shanghai, China).

#### Lentivirus construction and infection

RAW264.7 cells were provided by the Cell Bank of the CAS and cultured in DMEM with 10% FBS. To overexpress SIK2, the subcloned full-length cDNA for SIK2 was synthesized and inserted into a lentiviral vector (Ubi-MCS-CBh-gcGFP-IRES-puromycin). For lentiviral infection, RAW264.7 cells were seeded into 24-well plates at a density of 5 × 10^4^ per well and transduced with the LeSik2 or empty vector (WT) at a multiplicity of infection (MOI) of 100 for 48 hours. After 72 hours of culture, the gene expression was detected by fluorescence microscopy or immunoblotting. For the P38 inhibitor group, RAW264.7 cells were incubated with the P38 inhibitor SB 203580 (10 μM) for 24 hours.

#### Mouse CFs

CFs were prepared as previously described (*5*). To isolate fibroblasts from heart samples, single-cell suspensions were subjected to fluorescence-activated cell sorting (FACS) using antibodies against CD31-PE (553373, BD Biosciences), CD45-APC-Cy7 (557659, BD Biosciences), and MEFSK4-APC (130-120-802, Miltenyi Biotec, Bergisch Gladbach, Germany). Coverslips containing CFs were fixed with 4% paraformaldehyde for 10 min at room temperature. Subsequently, the cells were immunostained for the expression of α-SMA and Collagen I.

#### Flow cytometry and cell sorting

The mice were placed under complete anesthesia, after which their hearts were quickly removed and perfused with ice-cold PBS to clear out blood cells. Afterward, the left ventricles from sham-operated mice, along with the infarct and border regions of MI hearts, were dissected, finely chopped, and enzymatically digested in HBSS (Hanks’ balanced salt solution) containing collagenase type II (1.5 mg/ml; Sigma-Aldrich), DNase I (0.5 mg/ml; Sigma-Aldrich), and elastase (0.25 mg/ml; Sigma-Aldrich). The digestion was carried out for 1 hour at 37°C with gentle agitation. After digestion, the heart tissues were carefully triturated and filtered through a 70-μm cell strainer, followed by 1x RBC Lysis Buffer. The harvested cells were rinsed and resuspended in a 1x PBS containing sterile 2% FBS. Initially, single-cell suspensions were treated with anti-murine CD16/32 antibody (14-0161-82, eBioscience, San Diego, CA, USA) to block nonspecific antibody interactions with Fc gamma receptors. Next, cells were stained using a mix of fluorophore-conjugated antibodies that targeted specific surface markers. The primary antibodies obtained from BD Biosciences included CD45-APC-Cy7 (557659), CD11b-FITC (557396), Ly6G-APC (560599), Ly6C-PE-Cy7 (560593), and F4/80-PE (565410). In addition, α-Actinin-FITC (130-119-766) from Miltenyi Biotec. Peripheral blood cells were collected from the retro-orbital vein. RBCs were lysed with 1x RBC Lysis Buffer, and the cells were subsequently incubated with an Fc blocker. Single-cell suspensions from mouse spleens and bone marrow were prepared as follows. For the spleen, tissues were mechanically dissociated and passed through a 70-μm cell strainer, followed by 1x RBC Lysis Buffer. For the bone marrow, cells were flushed from the femurs and tibias with cold PBS containing 2% FBS. Data acquisition was performed using the Beckman Coulter CytoFlex LX, and the resulting data were processed and analyzed with the FlowJo software. Cell sorting was performed using the Beckman Coulter CytoFlex SRT.

#### RNA extraction and qPCR

Total RNA was extracted from sorted macrophages using the RNAprep Pure Microsample total RNA extraction kit (DP420, TIANGEN, Beijing, China). Total RNA from human PBMCs, cardiac tissue, RAW264.7 cells, and PMs was isolated using the TRIzol reagent (Thermo Fisher Scientific, Waltham, MA, USA). Following the manufacturer’s instructions, cDNA was synthesized from mRNA using the Reverse Transcription Kit (RT3/RT3-L, EZBioscience, Suzhou, China). Reverse transcription was performed at 42°C for 15 min, followed by a 3-min inactivation step at 95°C. The generated cDNA fragments underwent amplification in a qPCR machine (Applied Biosystems, Waltham, MA, USA) using the SYBR Green PCR kit (Vazyme, Nanjing, China). The mRNA levels of the target genes were normalized against the endogenous glyceraldehyde-3-phosphate dehydrogenase (GAPDH) expression, and relative mRNA expression or fold change was determined using the ΔΔCT method. Primer sequences for the genes of interest can be found in table S9.

#### Protein extraction and Western blot

Cultured cells were suspended (myocardial tissues were homogenized) and sonicated in radioimmunoprecipitation assay (RIPA) lysis buffer (Millipore, Billerica, MA, USA) containing protease and phosphatase inhibitors (Selleckchem, Houston, TX, USA). The lysates were subjected to centrifugation, and the supernatants were collected as whole-cell protein extracts. The protein concentrations were assessed using the bicinchoninic acid (BCA) assay. Total protein was separated by SDS–polyacrylamide gel electrophoresis (PAGE) and then transferred to a polyvinylidene difluoride (PVDF) membrane. Primary antibodies used to specifically detect the proteins of interest were as follows: SIK2 (6919s, CST), GAPDH (G9545, Sigma-Aldrich), α-SMA (A5228, Sigma-Aldrich), Collagen I (AB765P, Sigma-Aldrich), Collagen III (AB7778, Abcam), CD31 (3528s, CST), Phospho-NF-κB P65 (Ser^536^) (3033s, CST), NF-κB P65 (8242s, CST), Phospho-P38 MAPK (Thr^180^/Tyr^182^) (4511s, CST), P38 MAPK (8690s, CST), α-Tubulin (66031-1, Proteintech), Histone H3 (68345-1, Proteintech), NOS2 (13120s, CST), FLAG-Tag (14793s, CST), HA-Tag (3724s, CST), TRAF1 (4710s, CST), TRAF2 (4724s, CST), TRAF4 (18527s, CST), TRAF6 (67591s, CST), His-Tag (12698s, CST), and Myc-Tag (2276s, CST). Anti-rabbit immunoglobulin G (IgG), horseradish peroxidase (HRP)–linked antibody (7074, CST) and anti-mouse IgG, HRP-linked antibody (7076, CST) were used to visualize the binding of primary antibodies in combination with the enhanced chemiluminescence (ECL) substrate. The protein bands were quantitatively analyzed using the ImageJ software.

#### Subcellular fractionation

Nuclear and cytosolic extracts were prepared by nuclear and cytoplasmic extraction kit (Thermo Fisher Scientific) following the manufacturer’s instruction. In brief, cultured PMs were washed with 1x PBS, lysed by adding protease inhibitor cocktail tablet to cytoplasmic extraction reagent I, and then incubated with cytoplasmic extraction reagent II. The supernatant (cytoplasmic extract) was transferred to a clean test tube, and then the insoluble portion was resuspended with a nuclear extraction reagent. After centrifugation, the supernatant (nuclear extract) was transferred to a clean test tube. The extract was used for immunoblotting.

#### Coimmunoprecipitation assay

RAW264.7 cells, cardiac macrophages and HEK293T cells were lysed in NP-40 buffer (P0013F, Beyotime, Shanghai, China) supplemented with protease inhibitors (Selleckchem). Following centrifugation, the collected supernatants were incubated with the specified antibodies for 2 hours at 4°C. The immune complexes were then pulled down using Protein A/G Plus-Agarose (sc-2003, Santa Cruz Biotechnology, Dallas, TX, USA) for 6 hours at 4°C and washed five times with RIPA buffer. After washing, the immunoprecipitated components were eluted by boiling in immunoblot sample loading buffer. For immunoblot analysis, the immunoprecipitates and input lysates were separated using SDS-PAGE, transferred onto PVDF membranes (Millipore), and detected with specific antibodies.

#### In vitro efferocytosis assay

Cardiac macrophages were isolated from WT mice and SIK2-cKO mice by flow cytometry sorting and plated in 12-well dishes. Cardiomyocytes of 8-week-old mice were digested with collagenase II for 30 min. The heart cells were preplated on tissue culture–treated dishes for 2 hours, and the nonadherent cells were collected for phagocytosis experiment. Cardiomyocytes were treated with ultraviolet light for 10 min to induce apoptosis and incubated at 37°C for 4 hours. Cells were then labeled with Calcein-AM (C2012, Beyotime) for 45 min. Apoptotic cardiomyocytes and cardiac macrophages were cocultured in a specific proportion (2:1 or 5:1) for 1 hour. Removed unengulfed cells and wash macrophages with 1x PBS. Calcein-AM–positive macrophages were detected using flow cytometry. Besides, the cells were fixed with 4% formaldehyde and imaged with a microscope (Carl Zeiss). As for RBC efferocytosis assay, PMs and RAW264.7 were cultured in a 6-well plate and starved overnight. Sheep RBCs (SBJ-RBC-S001, Sbjbio, Nanjing, China) were incubated in 1x PBS at 37°C for 4 days. After 1x PBS washing, RBCs were further labeled with CFDA-SE (CFSE) (C1031, Beyotime) at 37°C for 20 min. Subsequently, labeled RBCs were added to macrophages and incubated at 37°C for a specific time (1 or 2 hours) and a specific ratio (1:50 and 1:100). The unbound RBCs were washed out, and the CSFE-positive macrophages were detected using flow cytometry.

### Statistical analysis

All values are presented as the means ± SEM. The number of samples is detailed in the individual figure legends and represents biological replicates, not technical replicates. Exclusion criteria were preestablished before the study began. No samples or data points were omitted from the analysis. Statistical analysis was performed using the GraphPad Prism software (version 8.01). *R*^2^ and *P* values were calculated using Pearson correlation analysis. Data normality was determined using the Shapiro-Wilk test. Statistical analysis was conducted using Student’s *t* test or the nonparametric Mann-Whitney *U* test for two-group studies. For multiple comparisons, one-way or two-way analysis of variance (ANOVA) followed by the Holm-Sidak’s multiple comparisons test or nonparametric Kruskal-Wallis test with the Dunn post hoc test was used. Categorical variables were compared using the Fisher’s exact test. The cumulative survival probability was evaluated using the Kaplan-Meier method, and statistical differences were assessed using the log-rank test. Statistical significance was set at *P* < 0.05.

## References

[1] J. G. Jollis, C. B. Granger, J. K. Zègre-Hemsey, T. D. Henry, A. Goyal, J. E. Tamis-Holland, M. L. Roettig, M. J. Ali, W. J. French, R. Poudel, J. Zhao, R. H. Stone, A. K. Jacobs, Treatment time and in-hospital mortality among patients with ST-segment elevation myocardial infarction, 2018-2021. JAMA 328, 2033–2040 (2022). 10.1001/jama.2022.20149 PMC9638953 36335474

[2] J. Yap, J. Irei, J. Lozano-Gerona, S. Vanapruks, T. Bishop, W. A. Boisvert, Macrophages in cardiac remodelling after myocardial infarction. Nat. Rev. Cardiol. 20, 373–385 (2023). 10.1038/s41569-022-00823-5 36627513

[3] P. M. Ridker, B. M. Everett, T. Thuren, J. G. MacFadyen, W. H. Chang, C. Ballantyne, F. Fonseca, J. Nicolau, W. Koenig, S. D. Anker, J. J. P. Kastelein, J. H. Cornel, P. Pais, D. Pella, J. Genest, R. Cifkova, A. Lorenzatti, T. Forster, Z. Kobalava, L. Vida-Simiti, M. Flather, H. Shimokawa, H. Ogawa, M. Dellborg, P. R. F. Rossi, R. P. T. Troquay, P. Libby, R. J. Glynn, CANTOS Trial Group, Antiinflammatory therapy with canakinumab for atherosclerotic disease. N. Engl. J. Med. 377, 1119–1131 (2017). 10.1056/NEJMoa1707914 28845751

[4] S. Zhang, Y. Zhang, X. Duan, B. Wang, Z. Zhan, Targeting NPM1 epigenetically promotes postinfarction cardiac repair by reprogramming reparative macrophage metabolism. Circulation 149, 1982–2001 (2024). 10.1161/CIRCULATIONAHA.123.065506 PMC11175795 38390737

[5] D. Jia, H. Jiang, X. Weng, J. Wu, P. Bai, W. Yang, Z. Wang, K. Hu, A. Sun, J. Ge, Interleukin-35 promotes macrophage survival and improves wound healing after myocardial infarction in mice. Circ. Res. 124, 1323–1336 (2019). 10.1161/CIRCRESAHA.118.314569 30832557

[6] N. Wang, W. Wang, X. Wang, G. Mang, J. Chen, X. Yan, Z. Tong, Q. Yang, M. Wang, L. Chen, P. Sun, Y. Yang, J. Cui, M. Yang, Y. Zhang, D. Wang, J. Wu, M. Zhang, B. Yu, Histone lactylation boosts reparative gene activation post-myocardial infarction. Circ. Res. 131, 893–908 (2022). 10.1161/CIRCRESAHA.122.320488 36268709

[7] D. Jia, S. Chen, P. Bai, C. Luo, J. Liu, A. Sun, J. Ge, Cardiac resident macrophage-derived legumain improves cardiac repair by promoting clearance and degradation of apoptotic cardiomyocytes after myocardial infarction. Circulation 145, 1542–1556 (2022). 10.1161/CIRCULATIONAHA.121.057549 35430895

[8] I. Hilgendorf, S. Frantz, N. G. Frangogiannis, Repair of the infarcted heart: Cellular effectors, molecular mechanisms and therapeutic opportunities. Circ. Res. 134, 1718–1751 (2024). 10.1161/CIRCRESAHA.124.323658 PMC11164543 38843294

[9] J. J. Lv, H. Wang, C. Zhang, T. J. Zhang, H. L. Wei, Z. K. Liu, Y. H. Ma, Z. Yang, Q. He, L. J. Wang, L. L. Duan, Z. N. Chen, H. Bian, CD147 sparks atherosclerosis by driving M1 phenotype and impairing efferocytosis. Circ. Res. 134, 165–185 (2024). 10.1161/CIRCRESAHA.123.323223 38166463

[10] K. Clark, K. F. MacKenzie, K. Petkevicius, Y. Kristariyanto, J. Zhang, H. G. Choi, M. Peggie, L. Plater, P. G. A. Pedrioli, E. McIver, N. S. Gray, J. S. C. Arthur, P. Cohen, Phosphorylation of CRTC3 by the salt-inducible kinases controls the interconversion of classically activated and regulatory macrophages. Proc. Natl. Acad. Sci. U.S.A. 109, 16986–16991 (2012). 10.1073/pnas.1215450109 PMC3479463 23033494

[11] S. Popov, H. Takemori, T. Tokudome, Y. Mao, K. Otani, N. Mochizuki, N. Pires, M. J. Pinho, A. Franco-Cereceda, L. Torielli, M. Ferrandi, A. Hamsten, P. Soares-da-Silva, P. Eriksson, A. M. Bertorello, L. Brion, Lack of salt-inducible kinase 2 (SIK2) prevents the development of cardiac hypertrophy in response to chronic high-salt intake. PLOS ONE 9, e95771 (2014). 10.1371/journal.pone.0095771 PMC3994160 24752134

[12] A. A. Ahmed, Z. Lu, N. B. Jennings, D. Etemadmoghadam, L. Capalbo, R. O. Jacamo, N. Barbosa-Morais, X.-F. Le, Australian Ovarian Cancer Study Group, P. Vivas-Mejia, G. Lopez-Berestein, G. Grandjean, G. Bartholomeusz, W. Liao, M. Andreeff, D. Bowtell, D. M. Glover, A. K. Sood, R. C. Bast Jr., SIK2 is a centrosome kinase required for bipolar mitotic spindle formation that provides a potential target for therapy in ovarian cancer. Cancer Cell 18, 109–121 (2010). 10.1016/j.ccr.2010.06.018 PMC3954541 20708153

[13] Z. Lu, W. Mao, H. Yang, J. M. Santiago-O'Farrill, P. J. Rask, J. Mondal, H. Chen, C. Ivan, X. Liu, C.-G. Liu, Y. Xi, K. Masuda, E. M. Carrami, M. Chen, Y. Tang, L. Pang, D. S. Lakomy, G. A. Calin, H. Liang, A. A. Ahmed, H. Vankayalapati, R. C. Bast Jr., SIK2 inhibition enhances PARP inhibitor activity synergistically in ovarian and triple-negative breast cancers. J. Clin. Invest. 132, e146471 (2022). 10.1172/JCI146471 PMC9151707 35642638

[14] N. J. Darling, P. Cohen, Nuts and bolts of the salt-inducible kinases (SIKs). Biochem. J. 478, 1377–1397 (2021). 10.1042/BCJ20200502 PMC8057676 33861845

[15] E. H. Kim, V. I. Galchev, J. Y. Kim, S. A. Misek, T. K. Stevenson, M. D. Campbell, F. D. Pagani, S. M. Day, T. C. Johnson, J. G. Washburn, K. L. Vikstrom, D. E. Michele, D. E. Misek, M. V. Westfall, Differential protein expression and basal lamina remodeling in human heart failure. Proteomics Clin. Appl. 10, 585–596 (2016). 10.1002/prca.201500099 PMC5524561 26756417

[16] Y. Han, B. Duan, J. Wu, Y. Zheng, Y. Gu, X. Cai, C. Lu, X. Wu, Y. Li, X. Gu, Analysis of time series gene expression and DNA methylation reveals the molecular features of myocardial infarction progression. Front. Cardiovasc. Med. 9, 912454 (2022). 10.3389/fcvm.2022.912454 PMC9263976 35811717

[17] E. Witt, E. Hammer, M. Dorr, K. Weitmann, D. Beug, K. Lehnert, M. Nauck, U. Volker, S. B. Felix, S. Ameling, Correlation of gene expression and clinical parameters identifies a set of genes reflecting LV systolic dysfunction and morphological alterations. Physiol. Genomics 51, 356–367 (2019). 10.1152/physiolgenomics.00111.2018 31274368

[18] E. Ammirati, C. V. Cannistraci, N. A. Cristell, V. Vecchio, A. G. Palini, P. Tornvall, A. M. Paganoni, E. A. Miendlarzewska, L. M. Sangalli, A. Monello, J. Pernow, M. Björnstedt Bennermo, G. Marenzi, D. Hu, N. G. Uren, D. Cianflone, T. Ravasi, A. A. Manfredi, A. Maseri, Identification and predictive value of interleukin-6^+^ interleukin-10^+^ and interleukin-6^−^ interleukin-10^+^ cytokine patterns in ST-elevation acute myocardial infarction. Circ. Res. 111, 1336–1348 (2012). 10.1161/CIRCRESAHA.111.262477 22931953

[19] S. Cai, M. Zhao, B. Zhou, A. Yoshii, D. Bugg, O. Villet, A. Sahu, G. S. Olson, J. Davis, R. Tian, Mitochondrial dysfunction in macrophages promotes inflammation and suppresses repair after myocardial infarction. J. Clin. Invest. 133, e159498 (2023). 10.1172/JCI159498 PMC9927948 36480284

[20] K. Shirakawa, J. Endo, M. Kataoka, Y. Katsumata, N. Yoshida, T. Yamamoto, S. Isobe, H. Moriyama, S. Goto, H. Kitakata, T. Hiraide, K. Fukuda, M. Sano, IL (interleukin)-10-STAT3-galectin-3 axis is essential for osteopontin-producing reparative macrophage polarization after myocardial infarction. Circulation 138, 2021–2035 (2018). 10.1161/CIRCULATIONAHA.118.035047 29967195

[21] X. Wu, M. R. Reboll, M. Korf-Klingebiel, K. C. Wollert, Angiogenesis after acute myocardial infarction. Cardiovasc. Res. 117, 1257–1273 (2021). 10.1093/cvr/cvaa287 33063086

[22] Y. Li, M. Dong, Q. Wang, S. Kumar, R. Zhang, W. Cheng, J. Xiang, G. Wang, K. Ouyang, R. Zhou, Y. Xie, Y. Lu, J. Yi, H. Duan, J. Liu, HIMF deletion ameliorates acute myocardial ischemic injury by promoting macrophage transformation to reparative subtype. Basic Res. Cardiol. 116, 30 (2021). 10.1007/s00395-021-00867-7 PMC8064941 33893593

[23] L. Ji, X. Zhao, B. Zhang, L. Kang, W. Song, B. Zhao, W. Xie, L. Chen, X. Hu, Slc6a8-mediated creatine uptake and accumulation reprogram macrophage polarization via regulating cytokine responses. Immunity 51, 272–284.e7 (2019). 10.1016/j.immuni.2019.06.007 31399282

[24] J. Tang, Y. Shen, G. Chen, Q. Wan, K. Wang, J. Zhang, J. Qin, G. Liu, S. Zuo, B. Tao, Y. Yu, J. Wang, M. Lazarus, Y. Yu, Activation of E-prostanoid 3 receptor in macrophages facilitates cardiac healing after myocardial infarction. Nat. Commun. 8, 14656 (2017). 10.1038/ncomms14656 PMC5338035 28256515

[25] P.-Y. Bai, S.-Q. Chen, D.-L. Jia, L.-H. Pan, C.-B. Liu, J. Liu, W. Luo, Y. Yang, M.-Y. Sun, N.-F. Wan, W.-W. Rong, A.-J. Sun, J.-B. Ge, Environmental eustress improves postinfarction cardiac repair via enhancing cardiac macrophage survival. Sci. Adv. 8, eabm3436 (2022). 10.1126/sciadv.abm3436 PMC9045726 35476440

[26] Q. Fan, R. Tao, H. Zhang, H. Xie, L. Lu, T. Wang, M. Su, J. Hu, Q. Zhang, Q. Chen, Y. Iwakura, W. Shen, R. Zhang, X. Yan, Dectin-1 contributes to myocardial ischemia/reperfusion injury by regulating macrophage polarization and neutrophil infiltration. Circulation 139, 663–678 (2019). 10.1161/CIRCULATIONAHA.118.036044 30586706

[27] E. Boada-Romero, J. Martinez, B. L. Heckmann, D. R. Green, The clearance of dead cells by efferocytosis. Nat. Rev. Mol. Cell Biol. 21, 398–414 (2020). 10.1038/s41580-020-0232-1 PMC7392086 32251387

[28] S. Gong, M. Zhai, J. Shi, G. Yu, Z. Lei, Y. Shi, Y. Zeng, P. Ju, N. Yang, Z. Zhang, D. Zhang, J. Zhuang, Q. Yu, X. Zhang, W. Jian, W. Wang, W. Peng, TREM2 macrophage promotes cardiac repair in myocardial infarction by reprogramming metabolism via SLC25A53. Cell Death Differ. 31, 239–253 (2024). 10.1038/s41418-023-01252-8 PMC10850484 38182899

[29] A. Hochreiter-Hufford, K. S. Ravichandran, Clearing the dead: Apoptotic cell sensing, recognition, engulfment, and digestion. Cold Spring Harb. Perspect. Biol. 5, a008748 (2013). 10.1101/cshperspect.a008748 PMC3579390 23284042

[30] R. P. H. De Maeyer, R. C. van de Merwe, R. Louie, O. V. Bracken, O. P. Devine, D. R. Goldstein, M. Uddin, A. N. Akbar, D. W. Gilroy, Blocking elevated p38 MAPK restores efferocytosis and inflammatory resolution in the elderly. Nat. Immunol. 21, 615–625 (2020). 10.1038/s41590-020-0646-0 PMC7983074 32251403

[31] H.-H. Wang, C.-Y. Lin, S.-H. Su, C.-T. Chuang, Y.-L. Chang, T.-Y. Lee, S.-C. Lee, C.-J. Chang, Activation of salt-inducible kinase 2 promotes the viability of peritoneal mesothelial cells exposed to stress of peritoneal dialysis. Cell Death Dis. 7, e2298 (2016). 10.1038/cddis.2016.79 PMC4973365 27441650

[32] Y. Qian, Z. Wang, H. Lin, T. Lei, Z. Zhou, W. Huang, X. Wu, L. Zuo, J. Wu, Y. Liu, L. F. Wang, X. H. Guan, K. Y. Deng, M. Fu, H. B. Xin, TRIM47 is a novel endothelial activation factor that aggravates lipopolysaccharide-induced acute lung injury in mice via K63-linked ubiquitination of TRAF2. Signal Transduct. Target. Ther. 7, 148 (2022). 10.1038/s41392-022-00953-9 PMC9072678 35513381

[33] Y. Liu, K. Liu, Y. Huang, M. Sun, Q. Tian, S. Zhang, Y. Qin, TRIM25 promotes TNF-α-induced NF-κB activation through potentiating the K63-linked ubiquitination of TRAF2. J. Immunol. 204, 1499–1507 (2020). 10.4049/jimmunol.1900482 32024699

[34] S. E. Alvarez, K. B. Harikumar, N. C. Hait, J. Allegood, G. M. Strub, E. Y. Kim, M. Maceyka, H. Jiang, C. Luo, T. Kordula, S. Milstien, S. Spiegel, Sphingosine-1-phosphate is a missing cofactor for the E3 ubiquitin ligase TRAF2. Nature 465, 1084–1088 (2010). 10.1038/nature09128 PMC2946785 20577214

[35] X. Li, Y. Yang, J. D. Ashwell, TNF-RII and c-IAP1 mediate ubiquitination and degradation of TRAF2. Nature 416, 345–347 (2002). 10.1038/416345a 11907583

[36] S. Li, L. Wang, M. E. Dorf, PKC phosphorylation of TRAF2 mediates IKKα/β recruitment and K63-linked polyubiquitination. Mol. Cell 33, 30–42 (2009). 10.1016/j.molcel.2008.11.023 PMC2643372 19150425

[37] R. R. Shen, A. Y. Zhou, E. Kim, E. Lim, H. Habelhah, W. C. Hahn, IκB kinase ε phosphorylates TRAF2 to promote mammary epithelial cell transformation. Mol. Cell. Biol. 32, 4756–4768 (2012). 10.1128/MCB.00468-12 PMC3497603 23007157

[38] H. Hu, S. C. Sun, Ubiquitin signaling in immune responses. Cell Res. 26, 457–483 (2016). 10.1038/cr.2016.40 PMC4822134 27012466

[39] X. Fan, J. Han, L. Zhong, W. Zheng, R. Shao, Y. Zhang, S. Shi, S. Lin, Z. Huang, W. Huang, X. Cai, B. Ye, Macrophage-derived GSDMD plays an essential role in atherosclerosis and cross talk between macrophages via the mitochondria-STING-IRF3/NF-κB axis. Arterioscler. Thromb. Vasc. Biol. 44, 1365–1378 (2024). 10.1161/ATVBAHA.123.320612 38695170

[40] S. L. Petersen, T. T. Chen, D. A. Lawrence, S. A. Marsters, F. Gonzalvez, A. Ashkenazi, TRAF2 is a biologically important necroptosis suppressor. Cell Death Differ. 22, 1846–1857 (2015). 10.1038/cdd.2015.35 PMC4648330 25882049

[41] S. D. Prabhu, N. G. Frangogiannis, The biological basis for cardiac repair after myocardial infarction: From inflammation to fibrosis. Circ. Res. 119, 91–112 (2016). 10.1161/CIRCRESAHA.116.303577 PMC4922528 27340270

[42] M. Yang, L. Song, L. Wang, A. Yukht, H. Ruther, F. Li, M. Qin, H. Ghiasi, B. G. Sharifi, P. K. Shah, Deficiency of GATA3-positive macrophages improves cardiac function following myocardial infarction or pressure overload hypertrophy. J. Am. Coll. Cardiol. 72, 885–904 (2018). 10.1016/j.jacc.2018.05.061 PMC6145461 30115228

[43] A. Anzai, J. L. Choi, S. He, A. M. Fenn, M. Nairz, S. Rattik, C. S. McAlpine, J. E. Mindur, C. T. Chan, Y. Iwamoto, B. Tricot, G. R. Wojtkiewicz, R. Weissleder, P. Libby, M. Nahrendorf, J. R. Stone, B. Becher, F. K. Swirski, The infarcted myocardium solicits GM-CSF for the detrimental oversupply of inflammatory leukocytes. J. Exp. Med. 214, 3293–3310 (2017). 10.1084/jem.20170689 PMC5679174 28978634

[44] F. Miranda, D. Mannion, S. Liu, Y. Zheng, L. S. Mangala, C. Redondo, S. Herrero-Gonzalez, R. Xu, C. Taylor, D. F. Chedom, E. M. Carrami, A. Albukhari, D. Jiang, S. Pradeep, C. Rodriguez-Aguayo, G. Lopez-Berestein, E. Salah, K. R. Abdul Azeez, J. M. Elkins, L. Campo, K. A. Myers, D. Klotz, S. Bivona, S. Dhar, R. C. Bast Jr., H. Saya, H. G. Choi, N. S. Gray, R. Fischer, B. M. Kessler, C. Yau, A. K. Sood, T. Motohara, S. Knapp, A. A. Ahmed, Salt-inducible kinase 2 couples ovarian cancer cell metabolism with survival at the adipocyte-rich metastatic niche. Cancer Cell 30, 273–289 (2016). 10.1016/j.ccell.2016.06.020 27478041

[45] W. Ariyoshi, T. Okinaga, W. Chaweewannakorn, S. Akifusa, T. Nisihara, Mechanisms involved in enhancement of matrix metalloproteinase-9 expression in macrophages by interleukin-33. J. Cell. Physiol. 232, 3481–3495 (2017). 10.1002/jcp.25809 28105703

[46] K. Y. Howangyin, I. Zlatanova, C. Pinto, A. Ngkelo, C. Cochain, M. Rouanet, J. Vilar, M. Lemitre, C. Stockmann, B. K. Fleischmann, Z. Mallat, J. S. Silvestre, Myeloid-epithelial-reproductive receptor tyrosine kinase and milk fat globule epidermal growth factor 8 coordinately improve remodeling after myocardial infarction via local delivery of vascular endothelial growth factor. Circulation 133, 826–839 (2016). 10.1161/CIRCULATIONAHA.115.020857 PMC4767109 26819373

[47] N. G. Frangogiannis, The inflammatory response in myocardial injury, repair, and remodelling. Nat. Rev. Cardiol. 11, 255–265 (2014). 10.1038/nrcardio.2014.28 PMC4407144 24663091

[48] M. Chiong, Z. V. Wang, Z. Pedrozo, D. J. Cao, R. Troncoso, M. Ibacache, A. Criollo, A. Nemchenko, J. A. Hill, S. Lavandero, Cardiomyocyte death: Mechanisms and translational implications. Cell Death Dis. 2, e244 (2011). 10.1038/cddis.2011.130 PMC3252742 22190003

[49] Y. Z. Lu, B. Nayer, S. K. Singh, Y. K. Alshoubaki, E. Yuan, A. J. Park, K. Maruyama, S. Akira, M. M. Martino, CGRP sensory neurons promote tissue healing via neutrophils and macrophages. Nature 628, 604–611 (2024). 10.1038/s41586-024-07237-y PMC11023938 38538784

[50] P. K. Veerasubramanian, T. A. Wynn, J. Quan, F. J. Karlsson, Targeting TNF/TNFR superfamilies in immune-mediated inflammatory diseases. J. Exp. Med. 221, e20240806 (2024). 10.1084/jem.20240806 PMC11413425 39297883

[51] C. N. Serhan, B. D. Levy, Resolvins in inflammation: Emergence of the pro-resolving superfamily of mediators. J. Clin. Invest. 128, 2657–2669 (2018). 10.1172/JCI97943 PMC6025982 29757195

