# Supplementary material for: SIK2 antagonizes postinfarction cardiac repair by driving macrophage proinflammatory and impairing efferocytosis
Source: Sci Adv. 2026 Oct 16;12(42):eadw6624. doi: 10.1126/sciadv.adw6624 (PMC13674512; doi:10.1126/sciadv.adw6624)
Supplement: Supplementary file 1 [file sciadv.adw6624_sm.pdf]

Science Advances
AAAS

Supplementary Materials for

SIK2 antagonizes postinfarction cardiac repair by driving macrophage proinflammatory and impairing efferocytosis

Wu-Wei Rong *et al.*

Corresponding author: Ping-Jin Gao, gaopingjin@sibs.ac.cn; Xiao-Dong Li, flylxd@163.com; Ji-Guang Wang, jiguangw@163.com

Sci. Adv. **12**, eadw6624 (2026)
DOI: 10.1126/sciadv.adw6624

This PDF file includes:

Supplementary Materials and Methods
Figs. S1 to S20
Tables S1 to S9

Extended Methods

Myocardial infarction (MI) model. The mice were randomly subjected to either permanent ligation of the left anterior descending (LAD) artery to induce MI, or a sham operation without ligation to serve as the control group. In detail, the mice were anesthetized with 2% isoflurane using an inhalation delivery system. A 1-2 cm incision was made over the left chest, and a 6-0 silk suture was used to permanently ligate the LAD, positioned 2-3 mm below the tip of the left auricle. Mice that did not survive the first 24 hours post-surgery were excluded from the analysis. Mice with an echocardiographic ejection fraction (EF) exceeding 50% at 24 h after surgery were deemed surgical failures and excluded from this study. For the SIK2 inhibitor group, ARN3236 (HY-120856, Medchem Express, Monmouth Junction, NJ, USA) was intraperitoneally injected (30 mg/kg body weight) daily from the start of the MI surgery to the day 10. For SIK2 overexpression experiments, a recombinant adeno-associated virus (AAV) vector carrying a macrophage-specific SIK2, $SIK2^{T175A}$ plasmid or a negative control (NC) plasmid was generated by Gene-Chem Co. Ltd. (Shanghai, China). The AAV9 viral vector harbors a macrophage-specific F4/80 promoter. AAV9-F4/80-SIK2, AAV9-F4/80-$SIK2^{T175A}$ and AAV9-F4/80-NC viral titer of 1.2×10^{13} vector genomes (vg)/mL was used. Further, 50 μL of AAV9-F4/80-SIK2, AAV9-F4/80-$SIK2^{T175A}$ or AAV9-F4/80-NC were injected into the tail vein of 4-week-old C57BL/6 wild type mice 4 weeks before MI. For the P38 inhibitor group, SB 203580 (HY-10256, Medchem Express) was intraperitoneally injected (5 mg/kg body weight) daily from the start of the MI surgery until day 10.

In vivo randomization and blinding procedures. Based on an a priori power analysis conducted using G*Power 3.1.9.7, the sample size for mice was determined (two-tailed test, statistical power=80%, α=0.05). The effect size was estimated based on preliminary experimental results and findings reported in the literature. The analysis indicated that a minimum of five mice per group was required for comparative studies. Randomization and allocation concealment procedures were implemented: mice were

randomly assigned to groups using a random number generator. All animal experiments were performed under blinded conditions, and data collection and analysis were carried out by personnel who were unaware of the animal group assignments or treatments administered.

Human samples. The diagnosis of acute myocardial infarction (MI) is based on an elevated cardiac troponin I level, with at least one value exceeding the 99th percentile upper reference limit, along with one or more of the following criteria: symptoms of ischemia, new ischemic changes or pathological Q waves on the ECG, imaging evidence of new myocardial tissue loss or abnormal regional wall motion, or angiographic detection of an intracoronary thrombus.

LC-MS/MS analysis. Liquid chromatography with tandem mass spectrometry analysis was performed by the Analysis Center of Gene-Chem Co. Ltd. (Shanghai, China). Briefly, protein samples were separated by SDS-PAGE gels, and gel pieces were excised. LC-MS/MS analysis was performed on a Q Exactive mass spectrometer (Thermo Fisher Scientific) that was coupled to Easy nLC (Thermo Fisher Scientific) for 60 min. SequestHT was used for tandem mass spectrometry analysis of the UniProt human proteome database.

Quantitative phosphoproteomic analysis. Quantitative phosphorylation proteomic profiling was performed by Oebiotech Co. Ltd. (Shanghai, China). In brief, total proteins were extracted from peritoneal macrophages treated with ARN3236 (3 μmol/L) or control. Part of the sample was selected for protein concentration determination and SDS-PAGE detection; the other part was subjected for trypsin digestion, peptide desalting, phosphorylated peptide enrichment and purification. Finally, the sample was analyzed using 4D Label-free and data analysis. The Maxquant search results were statistically analyzed and the phosphorylated confidently identified proteins/peptide segments/sites were filtered. The proteins corresponding to the phosphorylation sites

with significant differences were selected for functional analysis, and the functional annotation analysis of the identified proteins was performed using multiple common databases.

RNA-seq. RNA samples were sequenced and analyzed by BGI Tech Inc. Briefly, total RNA was extracted from infarcted macrophages and sequencing libraries were constructed. RNA sequencing was performed on the BGISEQ-500 platform. Raw sequencing reads were processed using SOAPnuke (v1.5.2) to remove low-quality reads, adaptor-containing reads, and reads with unknown bases. The filtered reads were then aligned to the mouse reference genome (mm10) using Bowtie 2 (v2.2.5), and gene expression levels were calculated using RSEM (v1.2.12). Differentially expressed genes (DEGs) between groups were identified using the DEGseq algorithm in R. The P-values for each gene were adjusted to Q-values (i.e., adjusted P-values) using Benjamini & Hochberg's False Discovery Rate (FDR) correction. For the methods described above, genes with a fold change ≥ 2 and a Q-value ≤ 0.05 were classified as DEGs. Using these DEGs and KEGG annotation results, pathway functional enrichment analysis was performed with the R function. The hypergeometric test with a False Discovery Rate (FDR) correction was applied to determine the significance of the enriched pathways, with a significance threshold of an adjusted P-value < 0.05.

Supplemental Figures

Fig. S1

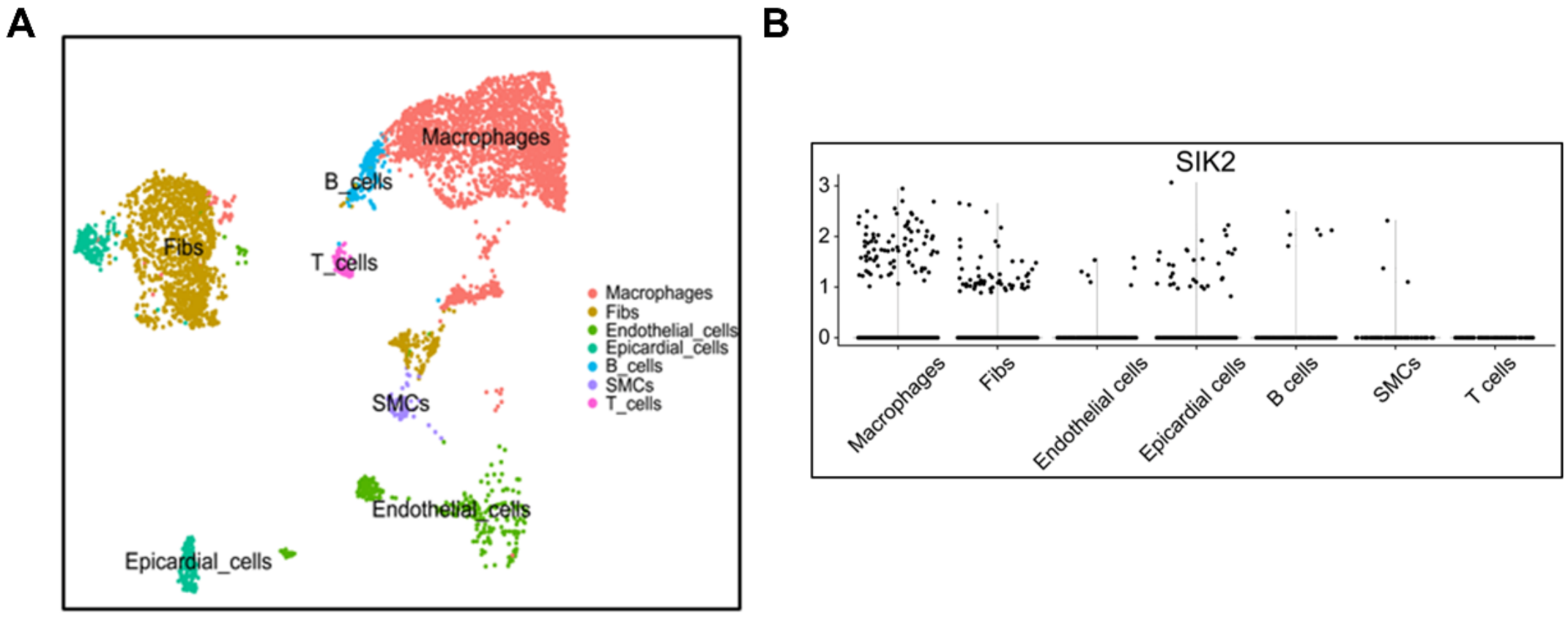

Fig. S1. Expression of salt-inducible kinase 2 (*Sik2*) in cardiac macrophages after myocardial infarction (MI). A, Annotated Uniform Manifold Approximation and Projection (UMAP) plot of mouse heart cells single-cell RNA sequencing after MI (GEO data set GSE136088). **B,** Scatterplot showing the distribution of *Sik2* in different cell clusters in mouse heart after MI.

Fig. S2

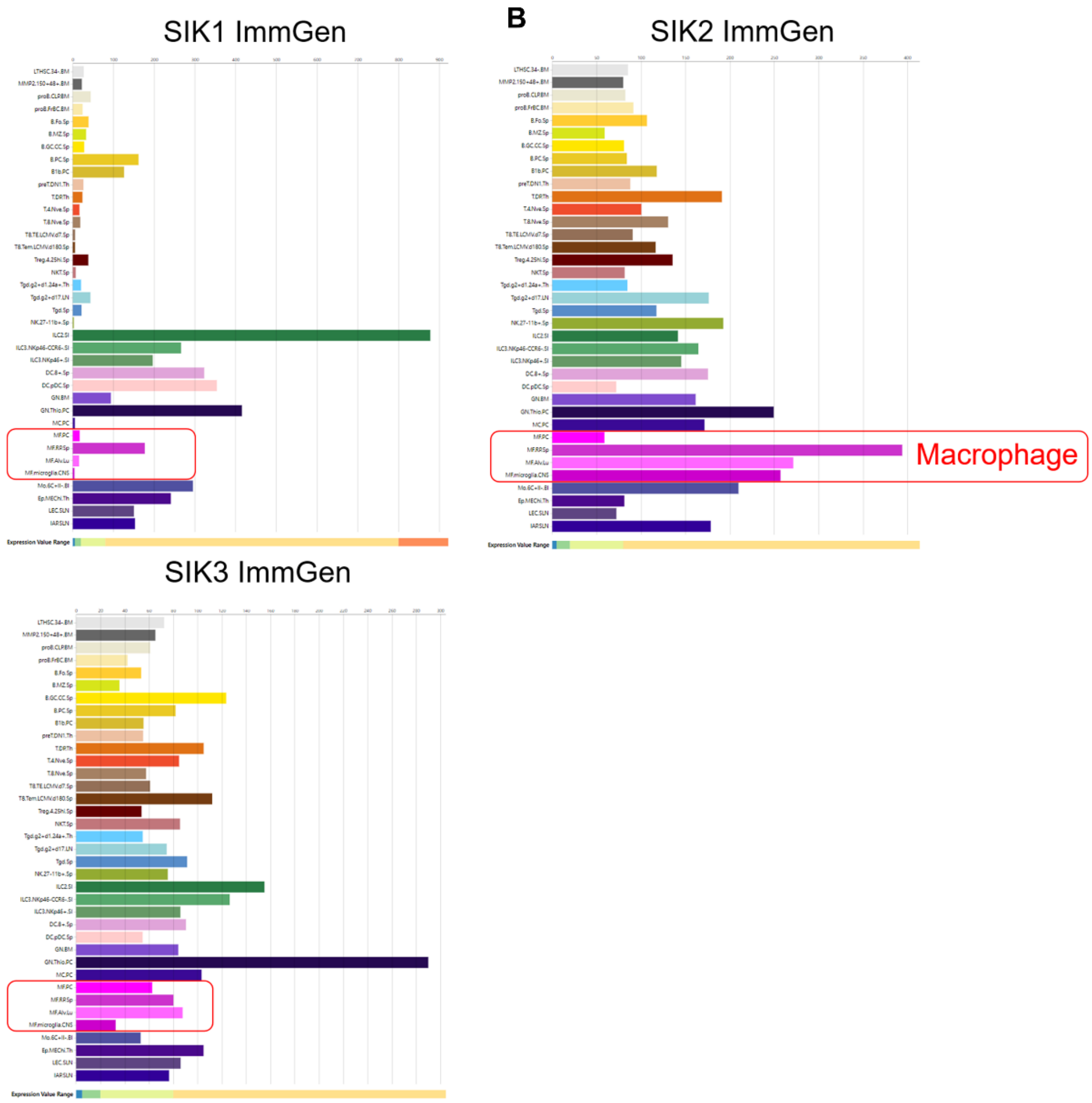

Fig. S2. *Siks* expression profile in immune cells using the ImmGen dataset. A, *Sik1* expression profile in immune cells using the ImmGen dataset. **B,** *Sik2* expression profile in immune cells using the ImmGen dataset. **C,** *Sik3* expression profile in immune cells using the ImmGen dataset.

Fig. S3

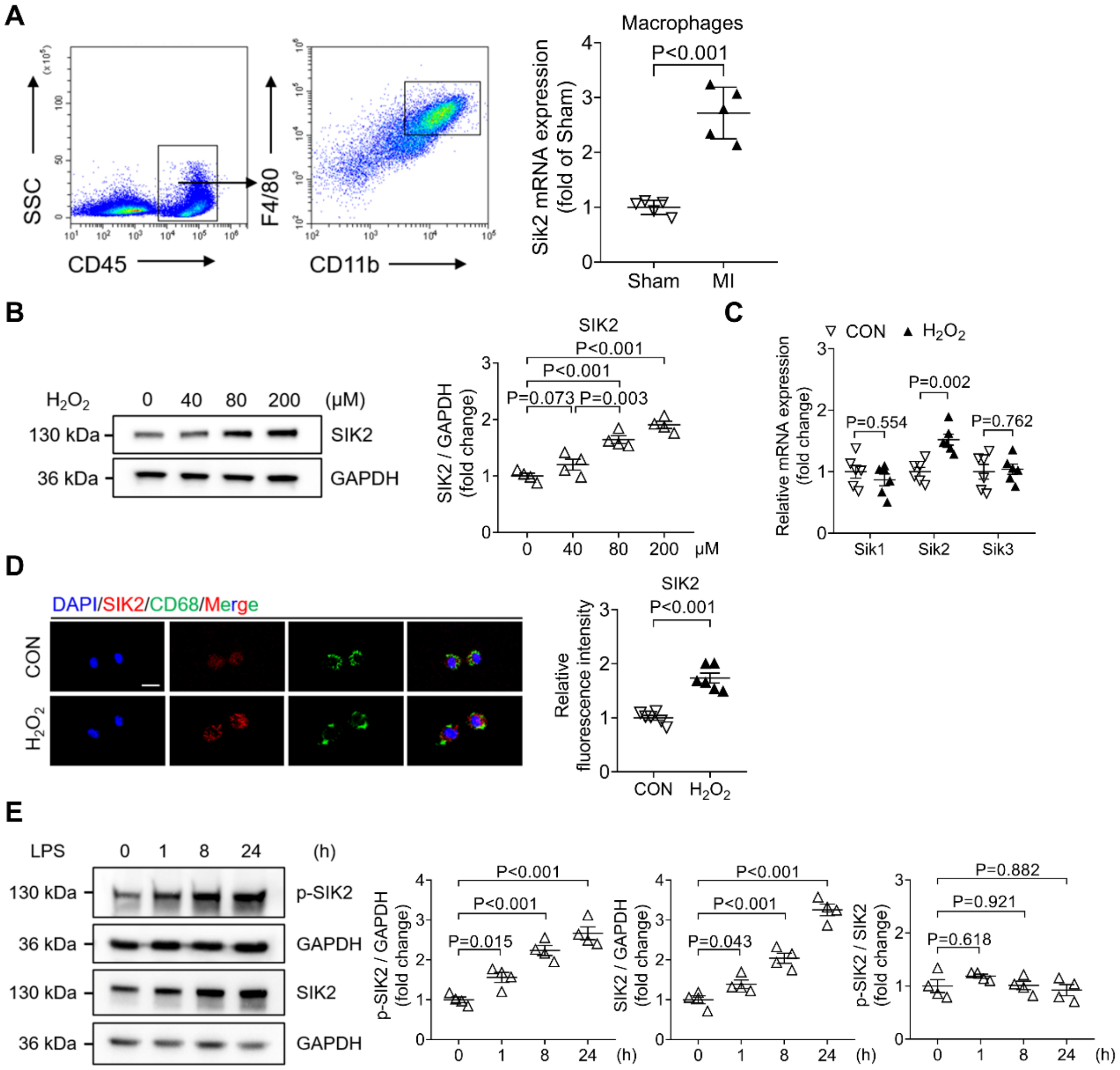

Fig. S3. SIK2 expression in macrophages under different stimuli. A, Flow cytometry gating scheme used to identify and characterize cardiac macrophage populations. Quantification of *Sik2* mRNA levels by quantitative polymerase chain reaction (qPCR) in cardiac macrophages (n=5). **B,** Dose dependence of the hydrogen peroxide (H_2O_2) effect on SIK2 protein expression in Raw264.7 macrophages. Immunoblotting analysis and quantification of SIK2 expression in Raw264.7 macrophages with the indicated concentration of H_2O_2 for 8 h (n=4). **C,** Quantification of *Sik1*, *Sik2*, *Sik3* mRNA levels by qPCR in Raw264.7 macrophages after 200 µM H_2O_2 stimulation for 8 h (n=6). **D,** Immunostaining analyses of SIK2 in Raw264.7 macrophages after 200 µM H_2O_2 stimulation for 8 h (n=6). CD68 (green), SIK2 (red) and 4′,6-diamidino-2-phenylindole (DAPI; blue). Scale bar, 20-µm. **E,** Effect of time-

course lipopolysaccharide (LPS) stimulation on p-SIK2 and SIK2 protein expression in Raw264.7 macrophages. Immunoblotting analysis and quantification of p-SIK2 and SIK2 expression in Raw264.7 macrophages with the indicated time point of LPS (n=4).

Fig. S4

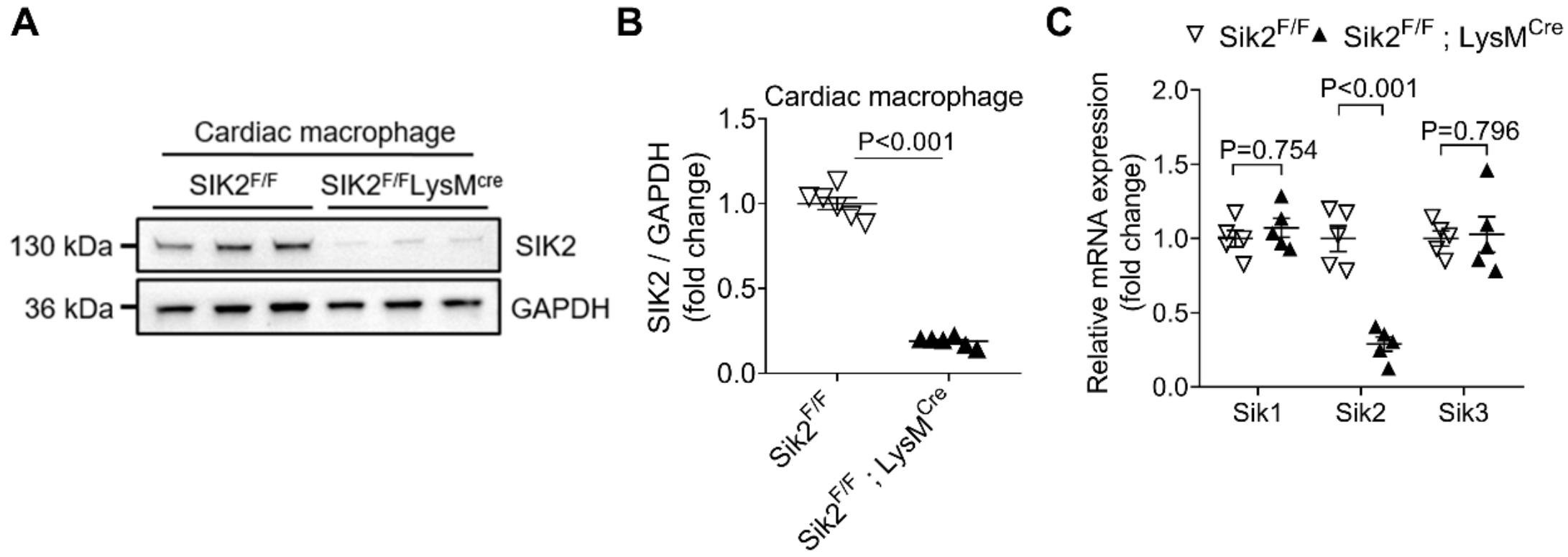

Fig. S4. Ablation of SIK2 in mouse macrophages. A and B, SIK2 protein expression and quantification of immunoblotting band intensity in cardiac macrophages isolated from heart in Sik2$^{F/F}$ and Sik2 $^{F/F}$; LysM^{Cre} mice (n=6).**C,** qPCR analysis of *Sik1, Sik2,* and *Sik3* mRNA expression in cardiac macrophages isolated from heart of Sik2$^{F/F}$ and Sik2 $^{F/F}$; LysM^{Cre} mice (n=5).

Fig. S5

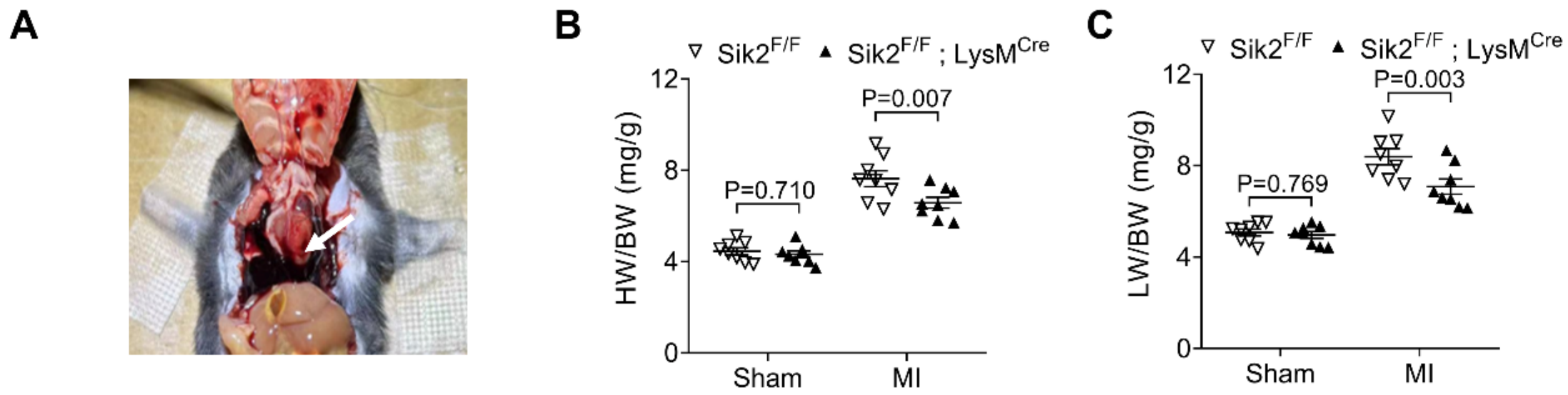

Fig. S5. Absence of macrophage SIK2 improves cardiac function after MI. A, Representative macroscopic view of a hemothorax after cardiac rupture. **B and C,** Statistical data of heart weight/body weight (HW/BW), lung weight/body weight (LW/BW) in Sik2$^{F/F}$ and Sik2 $^{F/F}$; LysM^{Cre} mice at day 14 after MI or sham operation (n=8).

Fig. S6

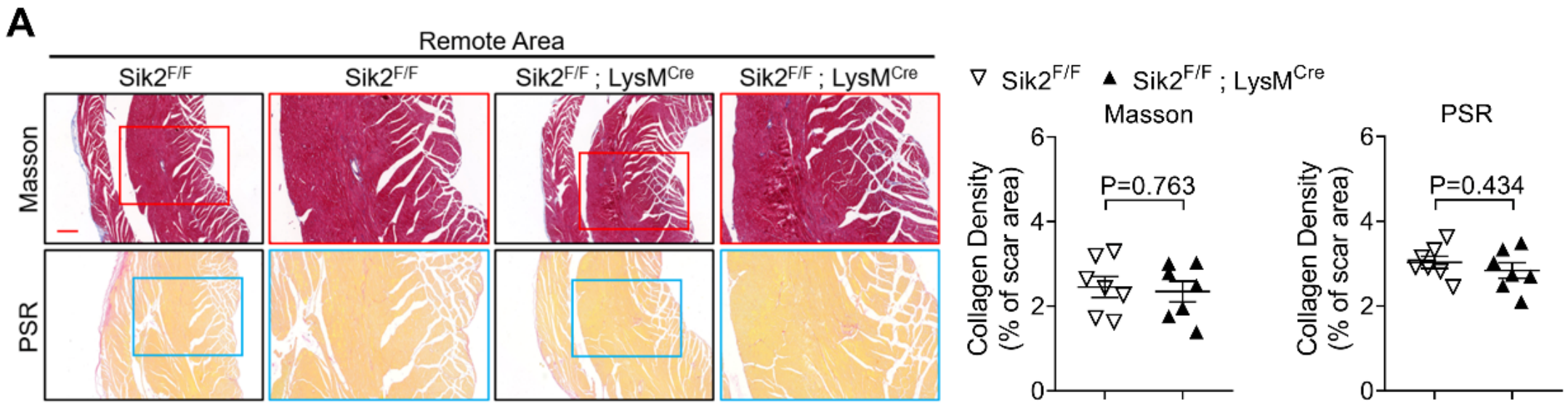

Fig. S6. Comparable collagen content in the non-infarct area. A, Masson's trichrome and Picrosirius red (PSR) staining of heart transverse sections to detect collagen within the remote area of $Sik2^{F/F}$ and $Sik2^{F/F}$; $LysM^{Cre}$ hearts 14 days after MI (scale bar, 100-µm). Collagen density measured as the total collagen area divided by remote area examined (n=7).

Fig. S7

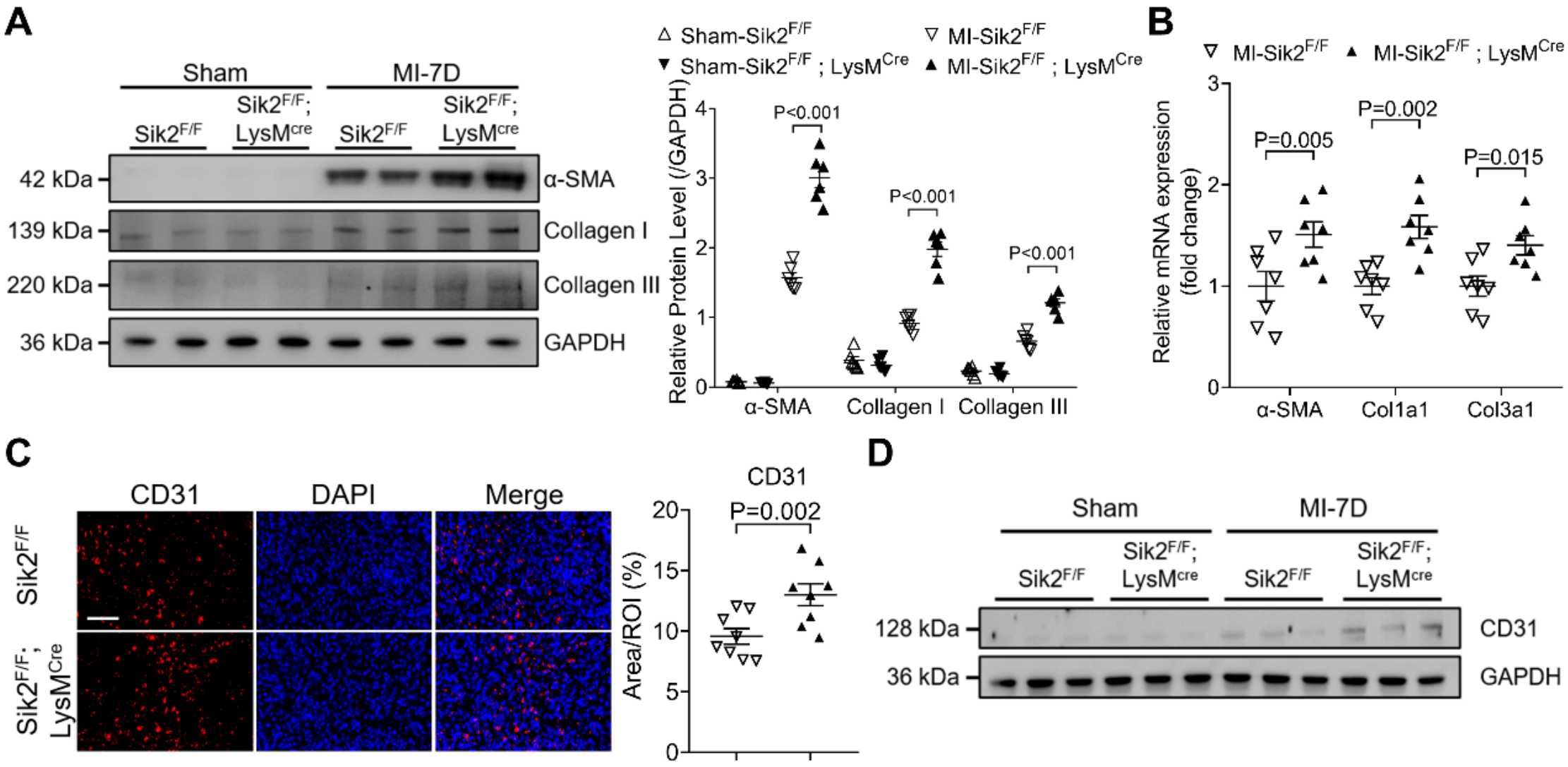

Fig. S7. Ablation of SIK2 in macrophages promotes myocardial repair and angiogenesis after MI. A, α-SMA, Collagen I and Collagen III protein expression and quantification of immunoblotting band intensity at day 7 post-MI or sham surgery in the scar tissue isolated from Sik2$^{F/F}$ and Sik2$^{F/F}$; LysM^{Cre} mice (n=6). **B,** qPCR analysis of α-*SMA, Col1a1,* and *Col3a1* mRNA expression in the scar tissue isolated from Sik2$^{F/F}$ and Sik2$^{F/F}$; LysM^{Cre} mice (n=7). **C,** Immunostaining analyses of CD31 in the heart tissue at day 7 post-MI (n=8). CD31(red), DAPI (blue). Scale bars, 40-μm. ROI indicates region of interest. **D,** Immunoblotting analysis of CD31 expression in the scar tissue isolated from Sik2$^{F/F}$ and Sik2$^{F/F}$; LysM^{Cre} mice at day 7 post-MI or sham surgery.

Fig. S8

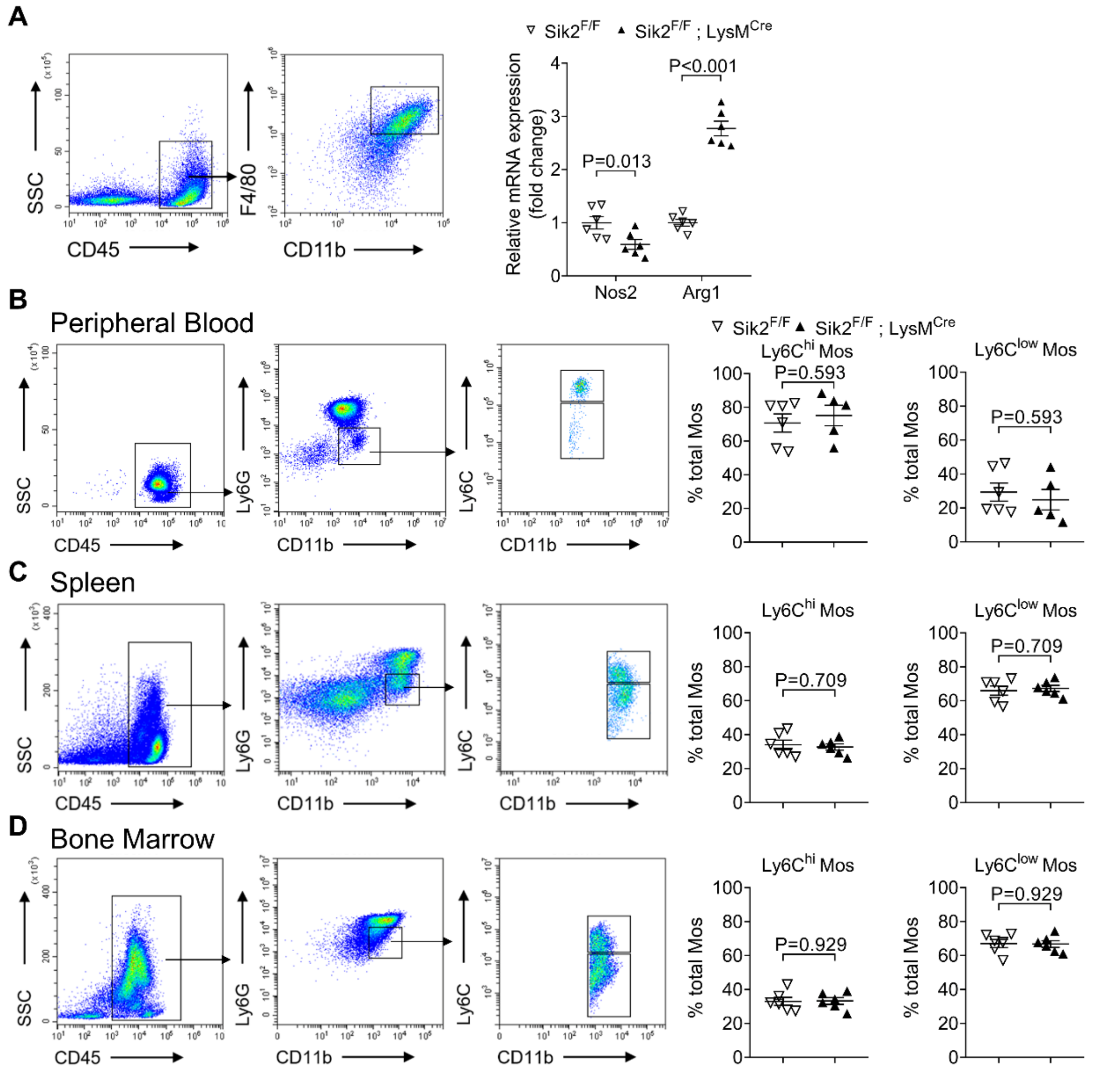

Fig. S8. Immune cells in the infarcted area, peripheral blood, spleen and bone marrow of mice with SIK2 loss-of-function. A, Flow cytometry gating scheme used to identify and characterize cardiac macrophage populations. Quantification of *Nos2,* and *Arg1* mRNA levels by qPCR in cardiac macrophages (n=6). **B,** Gating strategy for flow cytometric analysis of CD45$^+$CD11b$^+$Ly6G$^-$Ly6C^{hi} and CD45$^+$CD11b$^+$Ly6G$^-$Ly6C^{low} monocytes isolated from Sik2$^{F/F}$ and Sik2$^{F/F}$; LysM^{Cre} peripheral blood 5 days post MI (n=6 for Sik2$^{F/F,}$ n=5 for Sik2$^{F/F}$; LysM^{Cre}). **C,** Gating strategy for flow cytometric analysis of CD45$^+$CD11b$^+$Ly6G$^-$Ly6C^{hi} and CD45$^+$CD11b$^+$Ly6G$^-$Ly6C^{low} monocytes isolated from Sik2$^{F/F}$ and Sik2$^{F/F}$; LysM^{Cre} spleen 5 days post MI (n=6). **D,** Gating strategy for flow cytometric analysis of CD45$^+$CD11b$^+$Ly6G$^-$Ly6C^{hi} and

$CD45^+CD11b^+Ly6G^-Ly6C^{low}$ monocytes isolated from $Sik2^{F/F}$ and $Sik2^{F/F}$; $LysM^{Cre}$ bone marrow 5 days post MI (n=6).

Fig. S9

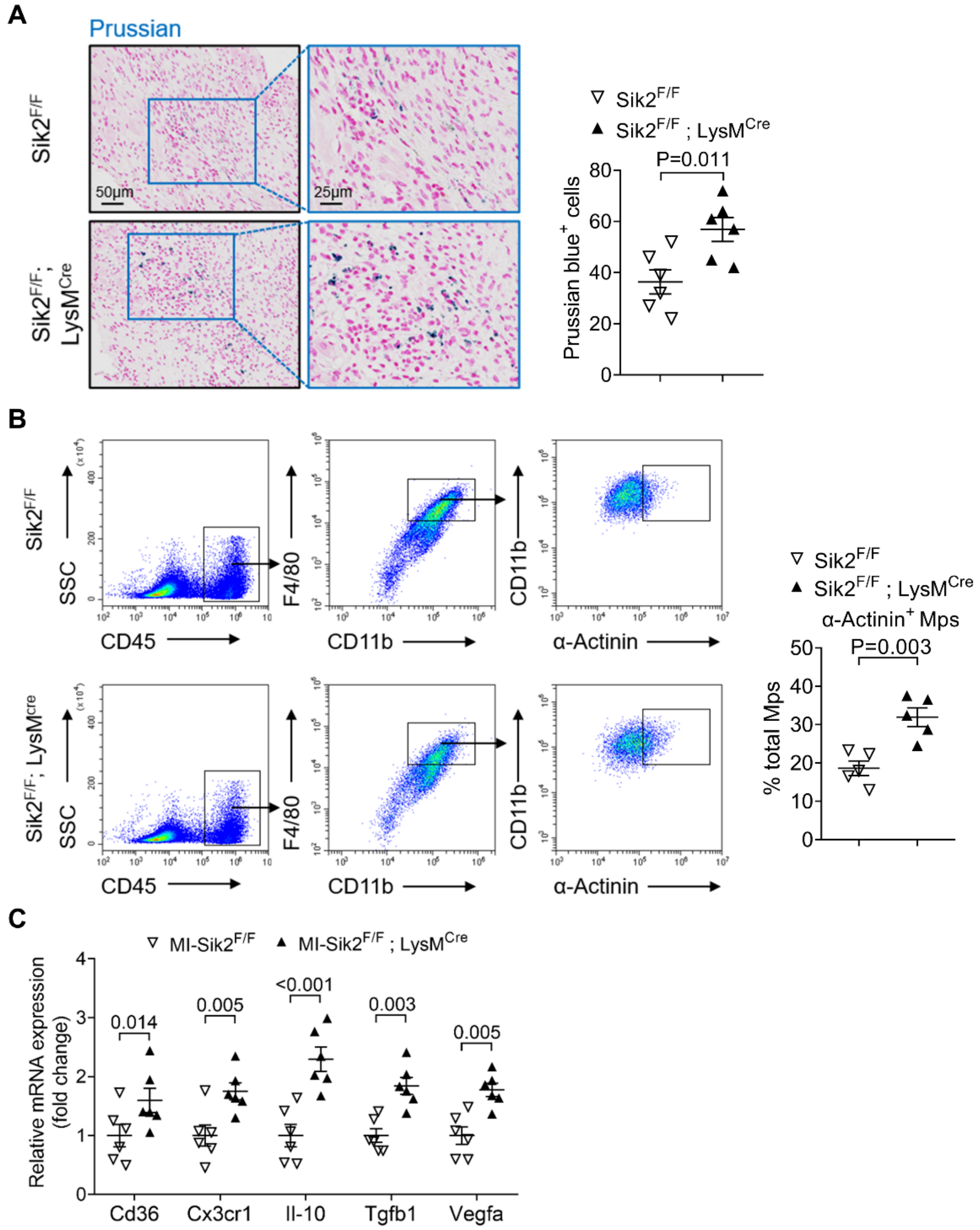

Fig. S9. Ablation of SIK2 in macrophages promotes efferocytosis after MI. A, Representative images and related quantification of Prussian blue staining for the phagocytosis of erythrocyte and cellular debris in macrophages in hearts of $Sik2^{F/F}$ and $Sik2^{F/F}$; $LysM^{Cre}$ mice (n=6). **B,** Gating strategy for flow cytometric analysis of $CD45^{+}CD11b^{+}F4/80^{+}\alpha\text{-Actinin}^{+}$ macrophages isolated from $Sik2^{F/F}$ and $Sik2^{F/F}$; $LysM^{Cre}$ infarcts 3 days post MI (n=5). **C,** mRNA expression levels of major reparative factors in $Sik2^{F/F}$ and $Sik2^{F/F}$; $LysM^{Cre}$ cardiac macrophages at day 5 post-MI (n=6).

Fig. S10

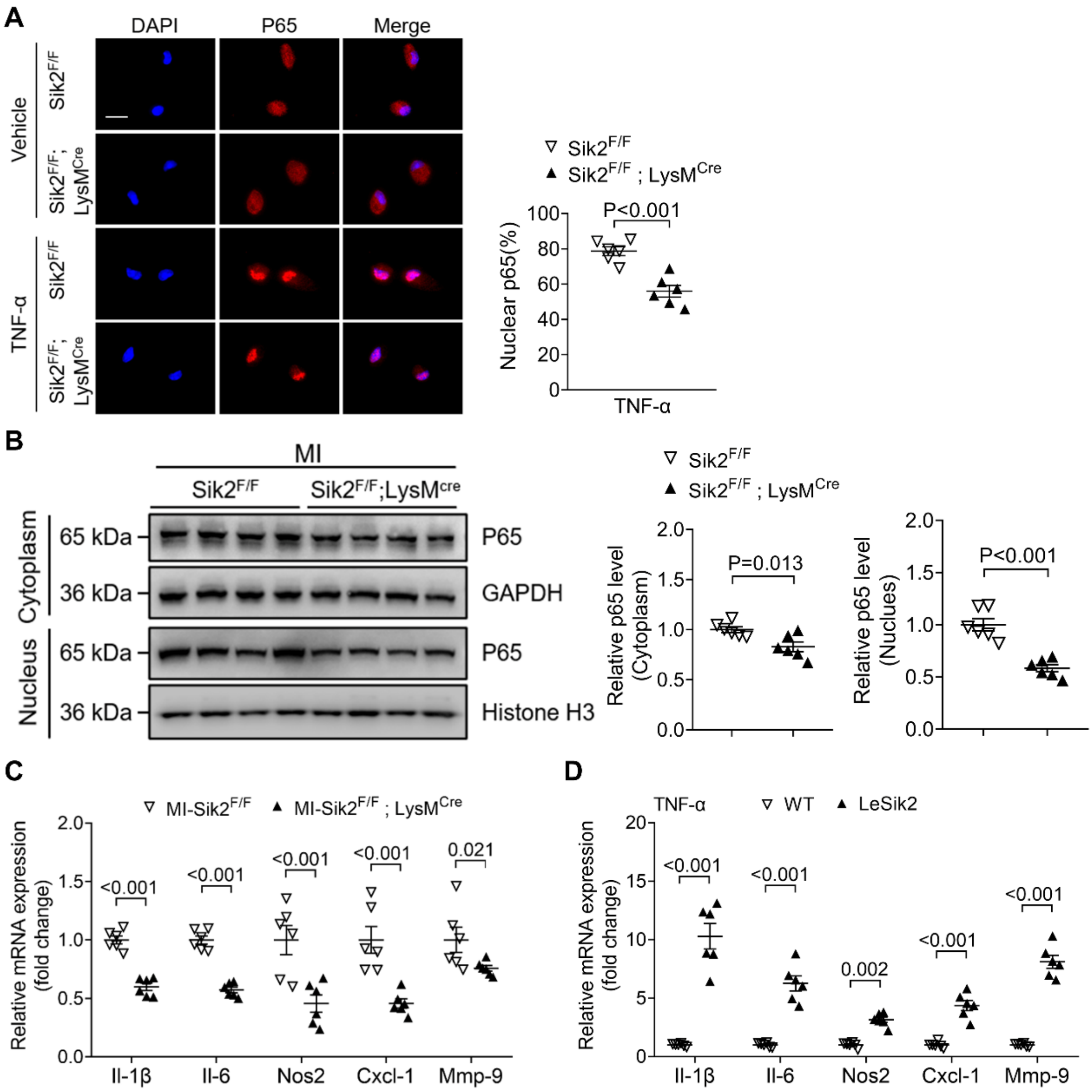

Fig. S10. SIK2 activates nuclear factor kappa B (NF-κB) signaling pathway in macrophages. A, Immunostaining analyses of nuclear P65 in Sik2$^{F/F}$ and Sik2$^{F/F}$; LysM^{Cre} peritoneal macrophages treated with tumor necrosis factor-α (TNF-α; 20 ng/mL) or vehicle (n=6). Scale bar, 20-µm. **B,** Immunoblotting analysis and quantification of P65 in cytoplasm and nucleus of Sik2$^{F/F}$ and Sik2$^{F/F}$; LysM^{Cre} cardiac macrophages at day 5 post-MI (n=6). **C,** qPCR analysis of *Il-1β, Il-6, Nos2, Cxcl-1,* and *Mmp-9* mRNA expression in Sik2$^{F/F}$ and Sik2$^{F/F}$; LysM^{Cre} cardiac macrophages at day 5 post-MI (n=6). **D,** qPCR analysis of *Il-1β, Il-6, Nos2, Cxcl-1,* and *Mmp-9* mRNA expression in WT or lentivirus Sik2 (LeSik2) Raw264.7 cells stimulated with TNF-α (n=6).

Fig. S11

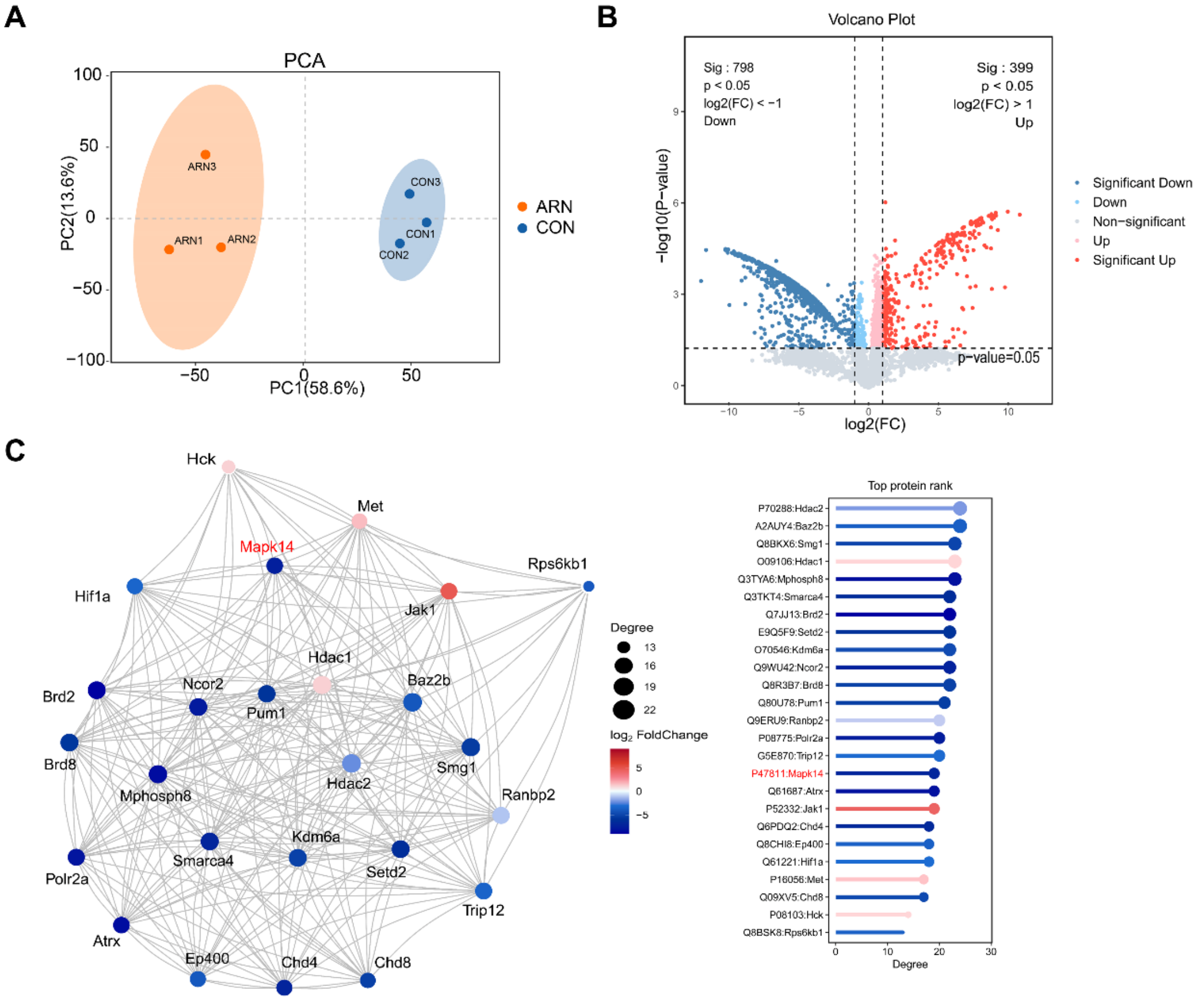

Fig. S11. Phosphorylated proteomics in macrophages. A, Principal component analysis (PCA) of phosphorylated proteomics expression values from peritoneal macrophages treated with ARN3236 (3 µmol) or control (n=3). **B,** Volcano plot displaying the differentially expressed genes (DEGs, fold change ≥2 and P<0.05) in peritoneal macrophages treated with ARN3236 (3 µmol) or control. The horizontal dash line represents a P value of 0.05, and the vertical dash lines correspond to 2-fold upregulation and downregulation. **C,** Protein-protein interaction (PPI) network displaying core differentially expressed phosphorylated protein.

Fig. S12

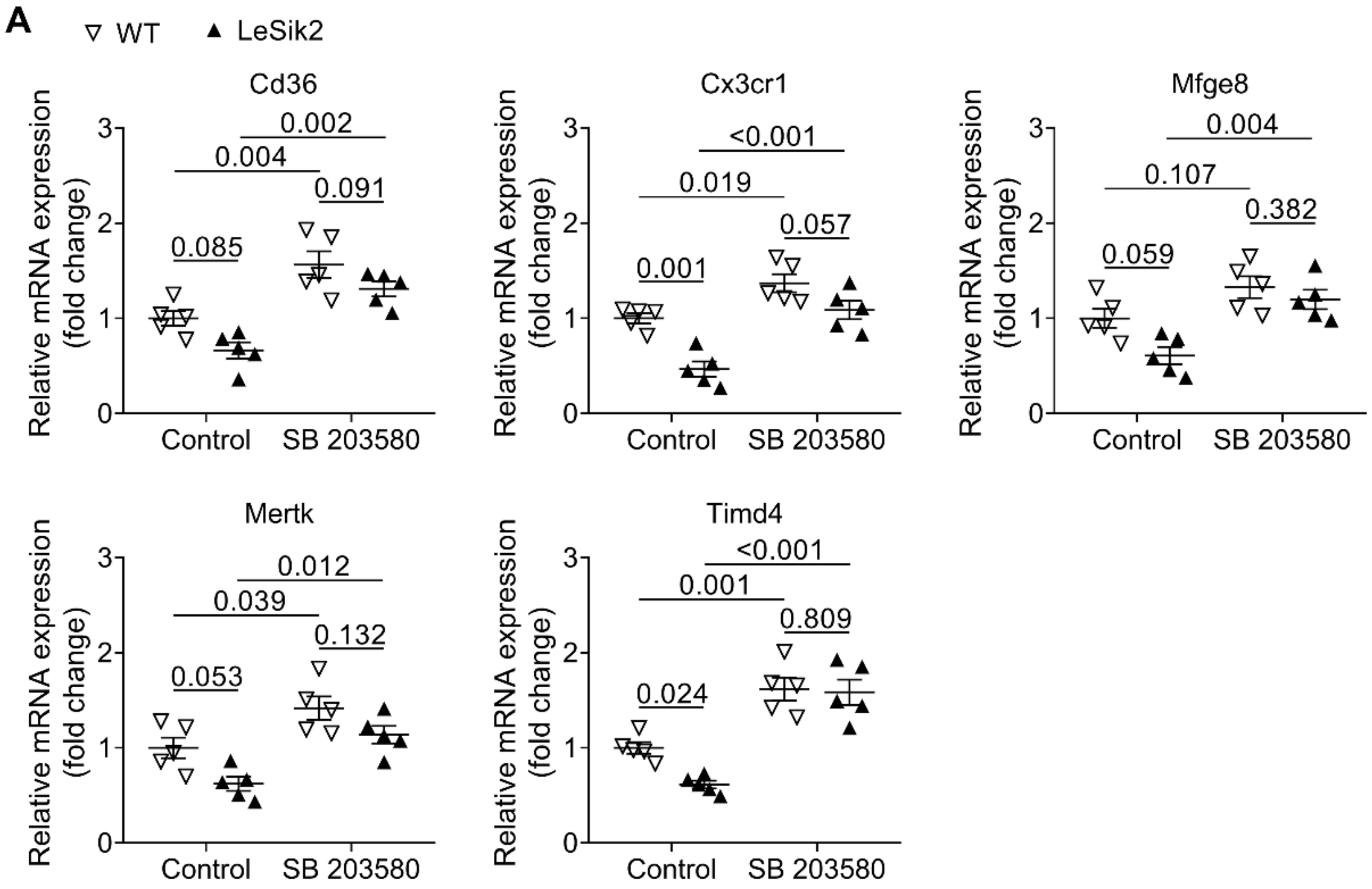

Fig. S12. SIK2-P38 pathway is essential for defective efferocytosis. A, qPCR analysis of *Cd36, Cx3cr1, Mfge8, Mertk,* and *Timd4* mRNA expression in WT or LeSik2 Raw264.7 cells stimulated with or without 10 µmol/L SB 203580 (n=5).

Fig. S13

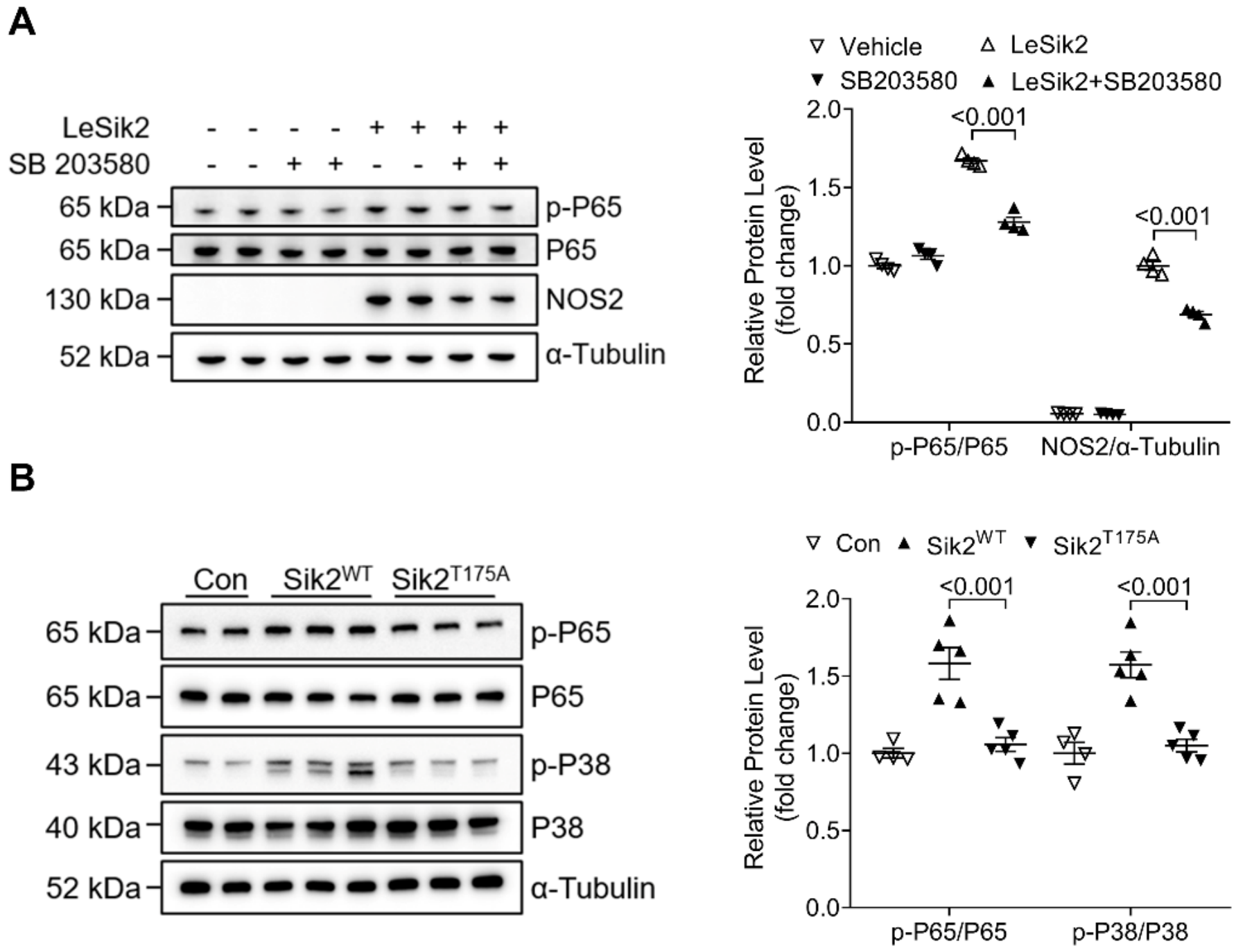

Fig. S13. SIK2 induces P65-NF-κB and P38-MAPK signaling dependent on Thr175 site. A, Immunoblotting analysis and quantification of p-P65/P65, and Nos2 in WT or LeSik2 Raw264.7 cells stimulated with SB 203580 (10μmol) or vehicle (n=4). **B,** Immunoblotting analysis and quantification of p-P65, P65, p-P38, and P38 expression in HEK293T cells transfected with control vector, SIK2^{WT} or SIK2^{T175A} mutant (n=4 for Con; n=5 for SIK2^{WT} and SIK2^{T175A}).

Fig. S14

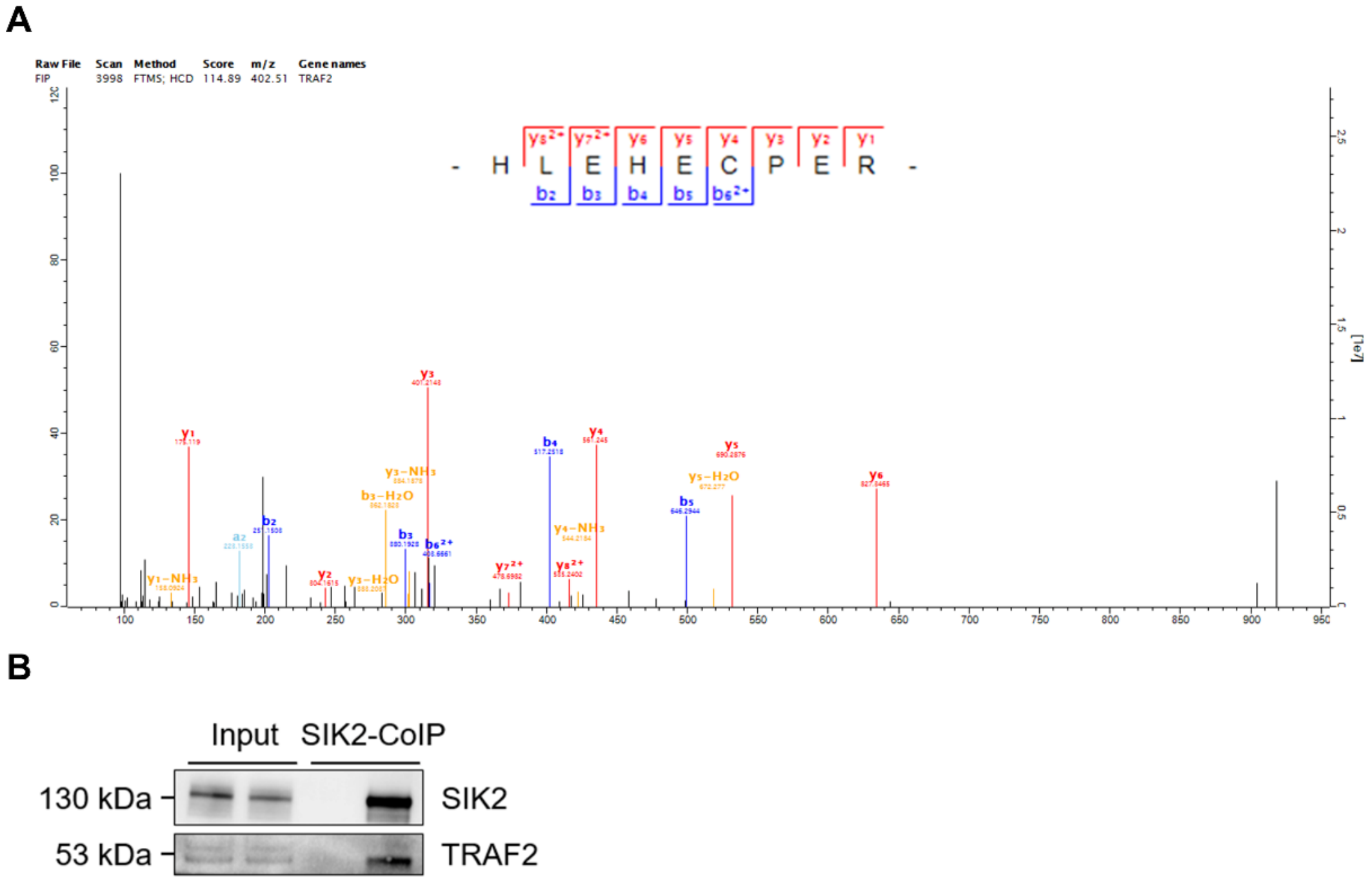

Fig. S14. SIK2 interacts with tumor necrosis factor receptor-associated factor 2 (TRAF2). A, Mass spectrometry analysis of the peptide showing HLEHECPER from TRAF2. **B,** Immunoprecipitation in macrophages using anti-SIK2 antibody, followed by immunoblotting with corresponding antibodies.

Fig. S15

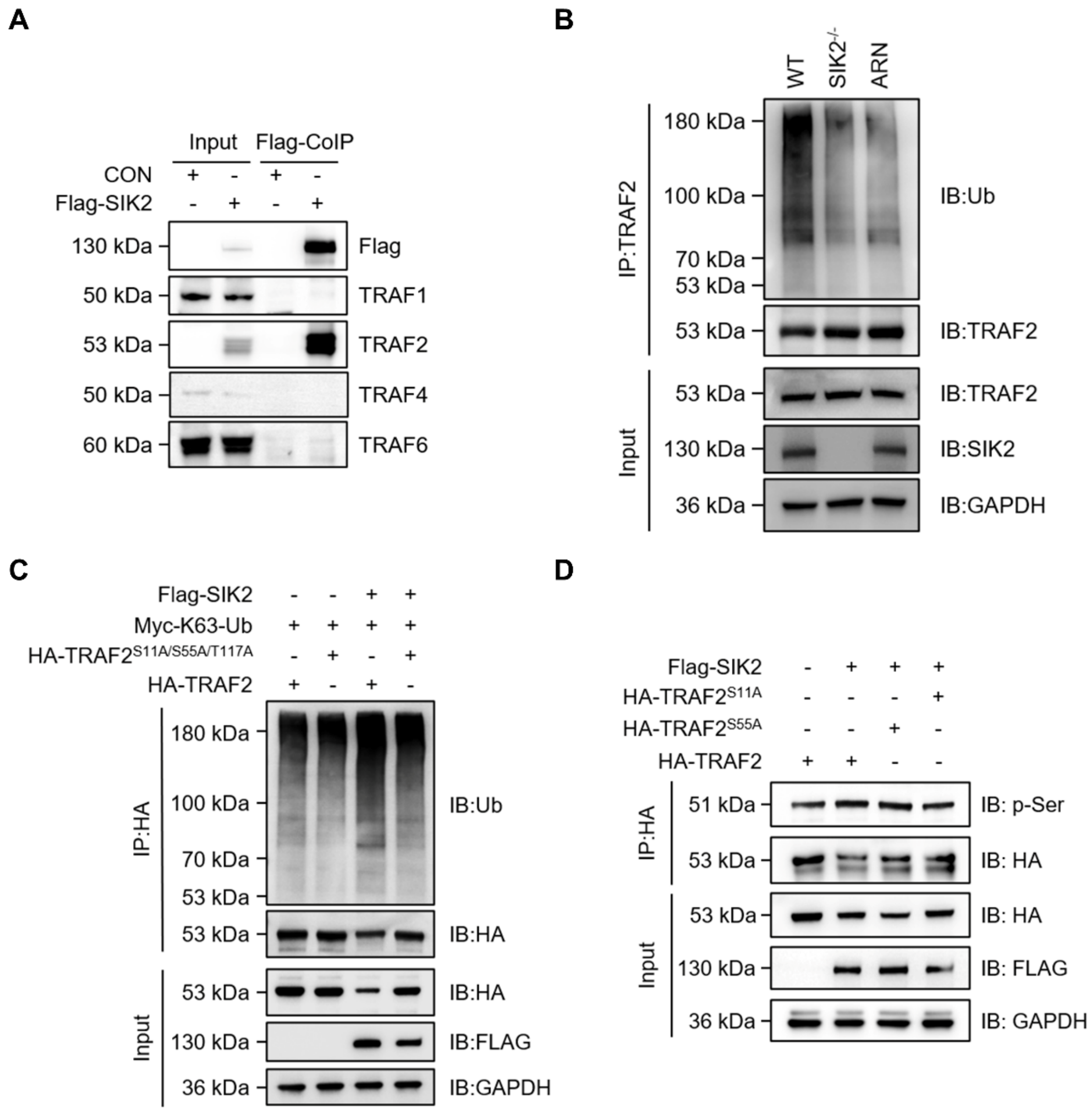

Fig. S15. SIK2 mediates TRAF2 K63-linked ubiquitylation through Ser11 phosphorylation. A, Immunoprecipitation in HEK293T cells transfected with Flag-SIK2 using anti-Flag antibody, followed by immunoblotting with corresponding antibodies. **B,** Immunoprecipitation using an anti-TRAF2 antibody, followed by immunoblotting with corresponding antibodies in cardiac macrophages from both SIK2-cKO and ARN3236-treated mice compared to WT controls. **C,** The Co-immunoprecipitation (Co-IP) analysis of TRAF2 ubiquitination in HEK293T cells co-transfected with Flag-SIK2 and Myc-K63-Ub, along with HA-TRAF2 or HA-TRAF2$^{S11A/S55A/T115A}$ mutant. **D,** The Co-IP analysis of TRAF2 phosphate serine (p-Ser) in HEK293T cells transfected with Flag-SIK2, along with HA-TRAF2, HA-TRAF2

^{S11A} mutant or HA-TRAF2 ^{S55A} mutant.

Fig. S16

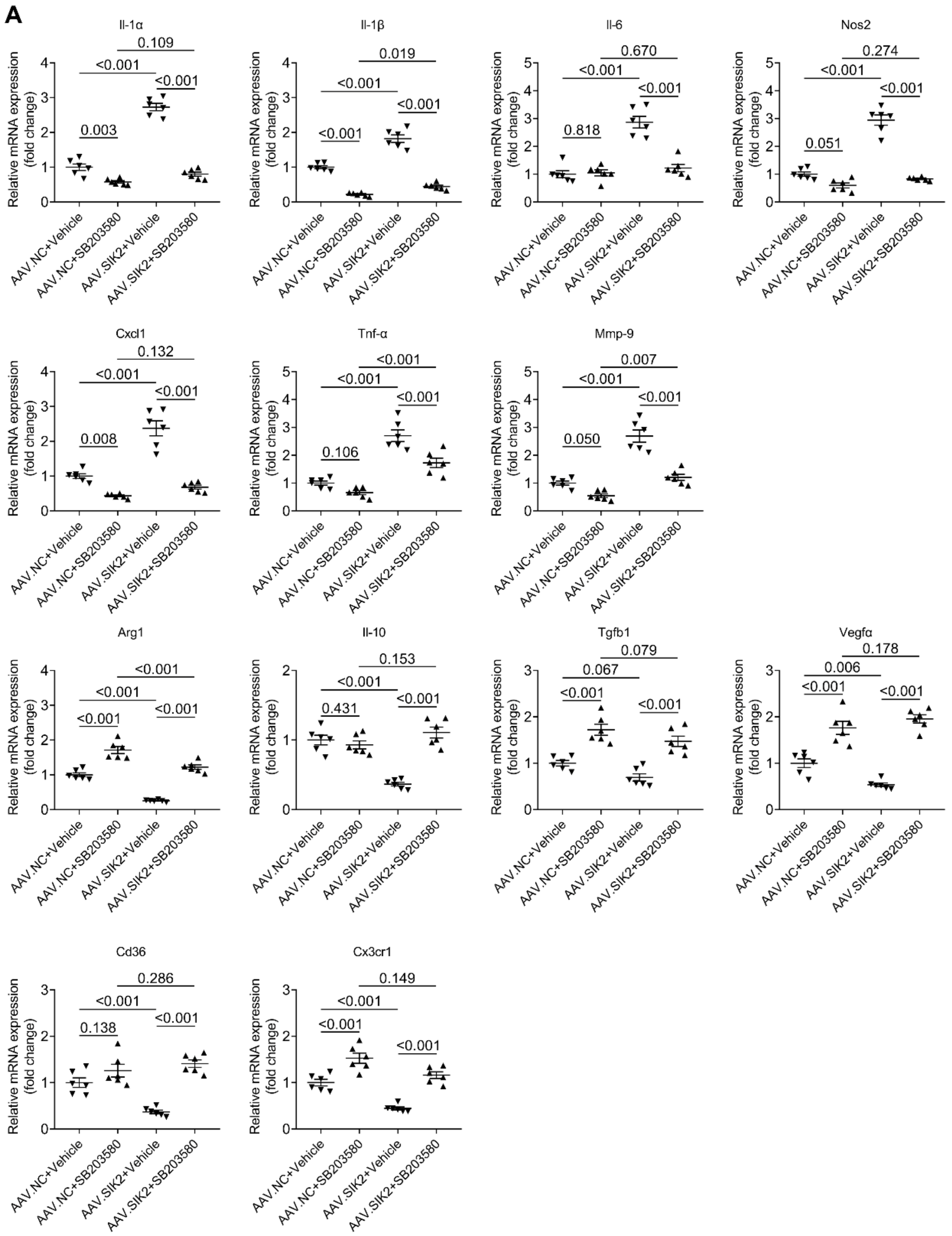

Fig. S16. Inhibition of P38 activation rescues myocardial repair and angiogenesis gene expression induced by SIK2 overexpression in MI. A, qPCR analysis of major proinflammatory and reparative factors in cardiac macrophages from WT mice administrated with AAV.F4/80.SIK2 or AAV.F4/80.NC treated with SB 203580 or vehicle at day 5 post-MI (n=6).

Fig. S17

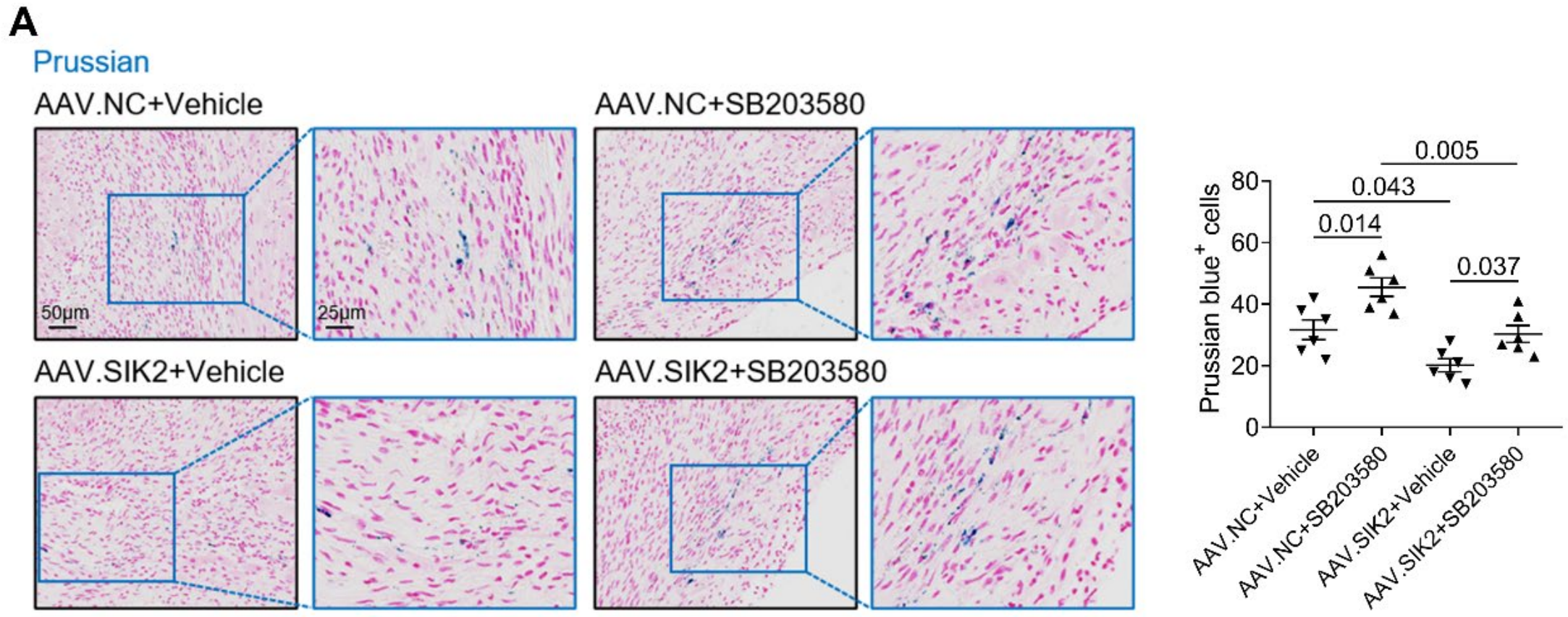

Fig. S17. Inhibition of P38 activation rescues impaired phagocytosis induced by SIK2 overexpression in MI. A, Representative images and related quantification of Prussian blue staining for the phagocytosis of erythrocyte and cellular debris in macrophages in hearts of WT mice administrated with AAV.F4/80.SIK2 or AAV.F4/80.NC treated with SB 203580 or vehicle (n=6).

Fig. S18

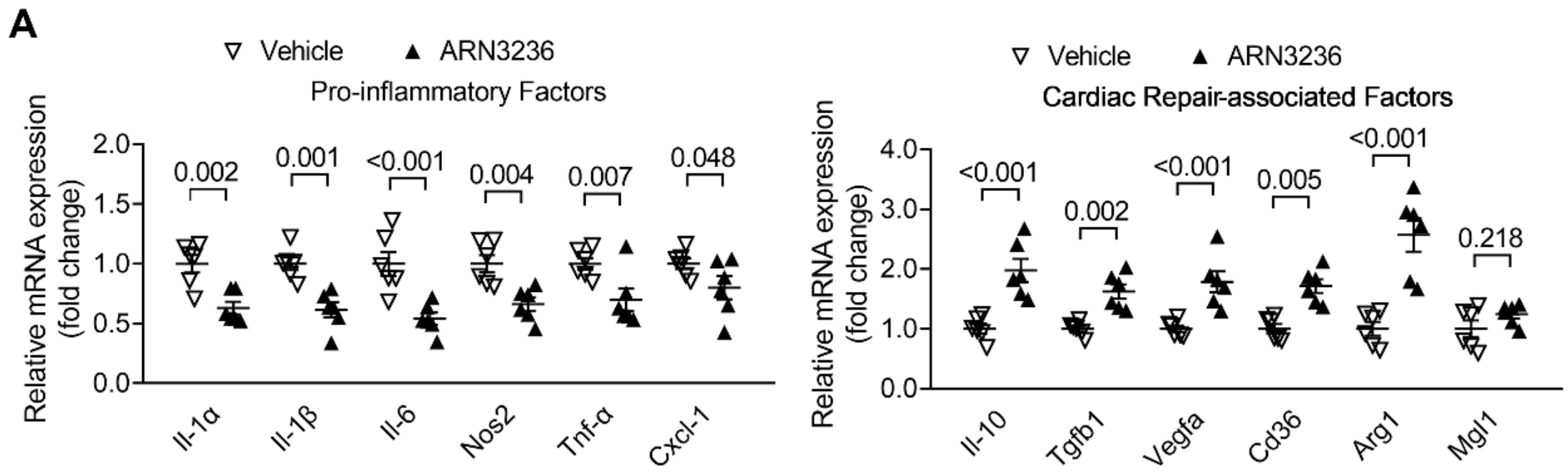

Fig. S18. Pharmacological blockade of SIK2 attenuates tissue repair and cardiac gene expression after MI. A, qPCR analysis of major proinflammatory and reparative factors in infarcts of WT mice administrated with ARN3236 or vehicle at day 5 post-MI (n=6).

Fig. S19

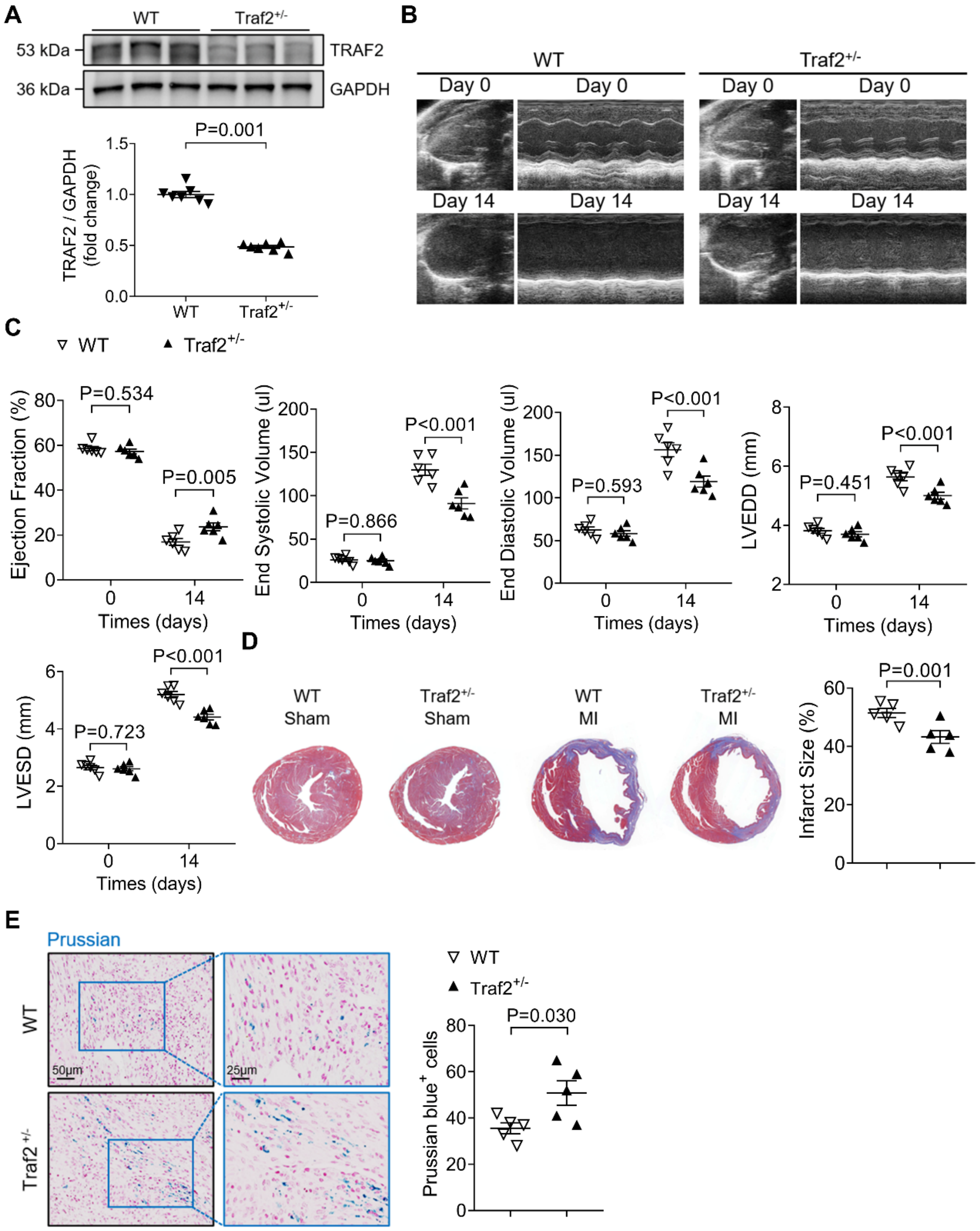

Fig. S19. Partial loss of TRAF2 deficiency improves myocardial function after MI. **A,** Immunoblotting analysis and quantification of TRAF2 in infarcts from WT and Traf2$^{+/-}$ mice (n=7). **B,** Representative parasternal long-axis views and M-mode images are shown. **C,** Summarized data of echocardiographic measurements in WT and Traf2$^{+/-}$ mice (n=6). **D,** Masson's trichrome staining for infarct measurements in designated groups at day 14 after MI (n=5). **E,** Representative images and related quantification of

Prussian blue staining for the phagocytosis of erythrocyte and cellular debris in macrophages in hearts of WT and $Traf2^{+/-}$ mice (n=5).

Fig. S20

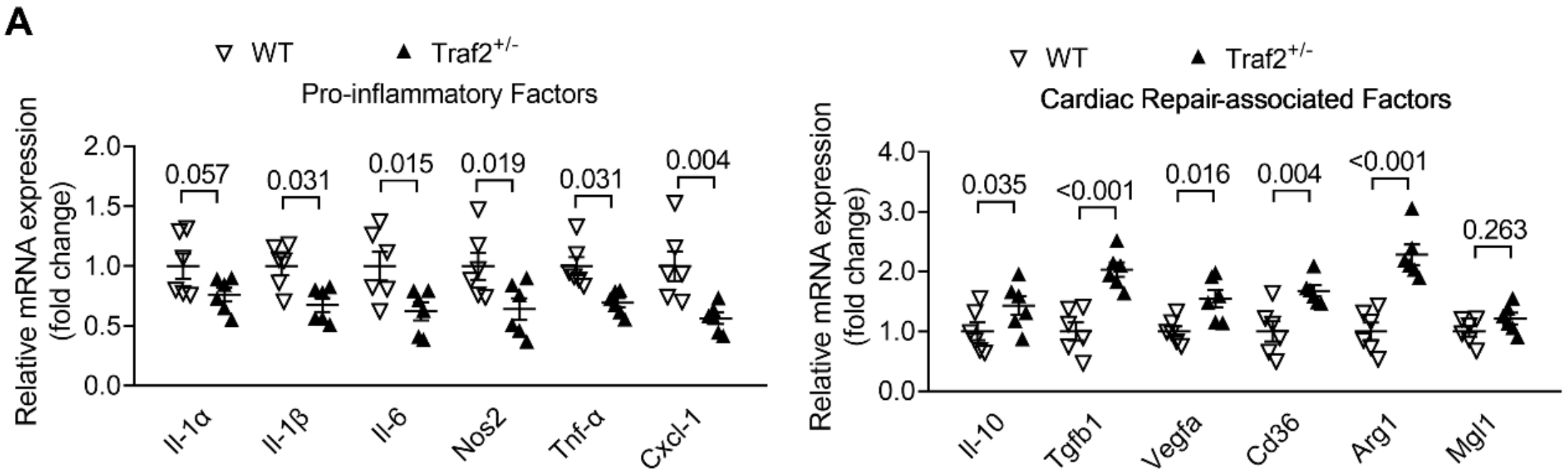

Fig. S20. Partial TRAF2 deficiency improves tissue repair and cardiac function gene expression after MI. A, qPCR analysis of major proinflammatory and reparative factors in infarcts of WT and Traf2$^{+/-}$ mice (n=6).

SUPPLEMENTAL TABLES

Table S1. Characteristics of the participants enrolled in this study.

Characteristics	MI group (n=20)	HC group (n=24)	p-value
Age (yrs)	61.85±1.92	60.75±1.75	0.674
Male gender (n, %)	16, 80.0%	16, 66.7%	0.498
BMI (kg/m^2)	25.11±0.62	24.23±0.61	0.317
Hypertension (n, %)	7, 35.0%	12, 50.0%	0.556
Hyperlipidemia (n, %)	10, 50.0%	9, 37.5%	0.543
Diabetes (n, %)	7, 35.0%	8, 33.3%	0.757
PCI therapy (n, %)	20, 100.0%	0, 0.00%	<0.001

Data are shown as mean ± SEM or frequencies (%). 20 MI patients and 24 healthy participants were recruited to donate their peripheral blood samples to this study. The diagnosis of MI was described in the Methods part. The Shapiro-Wilk test was used to ensure that the data was distributed normally. Comparisons of continuous variables were performed by Student's t-test for normally-distributed variables. The Mann-Whitney test was applied for non-normally distributed data. Comparisons of categorical variables were conducted with Fisher's exact test. Abbreviations: PCI, percutaneous coronary intervention; HC, healthy control; BMI, body mass index.

Table S2. Statistical descriptions of the indicators of coagulation, fibrinolysis, cholesterol, glucose, ions, hepatic and renal function in the enrolled MI patients.

Characteristics	Minimum	25% Percentile	Median	75% Percentile	Maximum	Mean	SD	SEM
D-dimer (mg/L)	0.09	0.22	0.30	0.37	1.09	0.39	0.32	0.07
FBG (mmol/L)	1.92	2.44	2.75	3.19	4.83	2.82	0.62	0.14
TC (mmol/L)	2.55	3.75	4.90	5.77	10.12	4.99	1.64	0.37
LDL (mmol/L)	0.96	2.36	3.28	4.05	6.26	3.21	1.23	0.28
HDL (mmol/L)	0.79	0.97	1.09	1.26	2.22	1.19	0.35	0.08
TG (mmol/L)	0.41	1.06	1.62	3.16	5.19	2.18	1.49	0.33
HbA1c (%)	5.00	5.75	6.20	7.00	10.20	6.56	1.25	0.28
Potassium (mmol/L)	3.16	3.84	3.98	4.10	4.50	3.97	0.32	0.07
Sodium (mmol/L)	134.2	138.2	139.0	140.0	142.0	138.9	1.94	0.43
Chlorine (mmol/L)	100.6	103.5	104.3	105.2	108.3	104.3	1.96	0.44
ALT (U/L)	14.70	23.03	36.90	45.30	66.50	36.92	15.46	3.46
AST (U/L)	27.18	60.71	70.77	76.90	88.53	61.35	67.08	15.7
Total bilirubin (μmol/L)	7.40	10.58	14.20	16.00	21.80	14.40	13.96	3.81
BUN (mmol/L)	2.90	4.36	4.88	6.27	7.98	5.25	1.35	0.30
Cr (μmol/L)	50.50	58.83	68.60	75.43	92.00	68.58	10.24	2.29

Clinical characteristics of 20 MI patients are shown.

Abbreviations: FBG, fasting blood glucose; TC, total cholesterol; LDL, low-density lipoprotein; HDL, high-density lipoprotein; TG, triglyceride; HbA1c, hemoglobin A1c; ALT, alanine aminotransferase; AST, aspartate aminotransferase; BUN, blood urea nitrogen; Cr, creatinine.

Table S3. Biometric and cardiac parameters of $Sik2^{F/F}$; $LysM^{cre}$ and $Sik2^{F/F}$ mice on days 14 post-MI.

Group	$Sik2^{F/F}$ Sham	$Sik2^{F/F}$; $LysM^{cre}$ Sham	$Sik2^{F/F}$ MI	$Sik2^{F/F}$;$LysM^{cre}$ MI
N	8	8	8	8
HW/BW (mg/g)	4.45±0.15	4.32±0.14	7.64±0.35	6.58±0.24$^{**p=0.007}$
LW/BW (mg/g)	5.08±0.14	4.97±0.15	8.39±0.35	7.08±0.33$^{**p=0.003}$
LVEDD (mm)	3.82±0.12	3.90±0.10	5.29±0.11	4.76±0.09$^{**p=0.003}$
LVESD (mm)	2.62±0.07	2.67±0.08	4.85±0.11	4.20±0.10$^{**p<0.001}$
FS (%)	31.50±0.79	30.81±0.76	8.10±0.51	11.53±0.51$^{**p=0.002}$
LVEDV (μL)	63.23±4.42	66.28±4.19	133.80±6.00	106.00±4.54$^{**p=0.001}$
LVESV (μL)	25.03±1.49	26.55±1.76	110.20±5.76	79.70±4.14$^{**p<0.001}$
EF (%)	60.09±1.08	59.92±1.38	17.80±1.06	24.98±1.05$^{**p<0.001}$
HR (beats/min)	477.44±8.03	485.43±4.84	479.18±10.01	484.25±5.89

Data are shown as mean ± SEM. Data were analyzed using two-way ANOVA with Holm-Sidak's multiple comparisons test. **p indicates $Sik2^{F/F}$ MI vs. $Sik2^{F/F}$; $LysM^{cre}$ MI.

N = number, HW /BW = heart weight/body weight; LW/BW = lung weight/body weight; LVEDD = left ventricular end-diastolic diameter; LVESD = left ventricular end-systolic diameter; FS = fractional shortening; LVEDV = left ventricular end-diastolic volume; LVESV = left ventricular end -systolic volume; EF = ejection fraction; HR = heart rate.

Table S4. Transcriptome analysis of mRNA expression alterations of SIK2-binding protein candidates in SIK2-cKO cardiac macrophages.

Gene name	LogFC	P-adjusted
Traf2	-0.2377251	1.5051874E-08
Irak1	-0.2377251	0.0693811
Chuk	-0.1380358	0.0004289

Table S5. Biometric and cardiac parameters of Wild-type (WT) mice administrated with adeno-associated virus (AAV).F4/80.SIK2 or AAV.F4/80.NC treated with SB 203580 or vehicle mice on days 14 post-MI.

Group	AAV.NC Vehicle MI	AAV.NC SB 203580 MI	AAV.SIK2 Vehicle MI	AAV.SIK2 SB 203580 MI
N	6	6	6	6
LVEDD (mm)	5.28±0.07	4.83±0.09	5.62±0.10$^{*p=0.016}$	4.91±0.07$^{\#\#p<0.001}$
LVESD (mm)	4.79±0.07	4.16±0.11	5.30±0.08$^{**p=0.002}$	4.31±0.09$^{\#\#p<0.001}$
FS (%)	9.19±0.63	13.96±0.86	5.60±0.43$^{**p=0.007}$	12.30±0.89$^{\#\#p<0.001}$
LVEDV (μL)	134.17±3.93	109.55±4.83	155.16±6.11$^{*p=0.015}$	113.71±3.58$^{\#\#p<0.001}$
LVESV (μL)	107.26±3.44	77.26±4.85	135.74±4.67$^{**p<0.001}$	83.82±4.03$^{\#\#p<0.001}$
EF (%)	20.04±1.29	29.72±1.67	12.42±0.91$^{**p=0.004}$	26.42±1.75$^{\#\#p<0.001}$
HR (beats/min)	489.86±4.44	477.02±9.39	474.79±8.02	489.00±3.45

Data are shown as mean ± SEM. Data were analyzed using two-way ANOVA with Holm-Sidak's multiple comparisons test. *, **p indicates AAV.NC Vehicle MI vs. AAV.SIK2 Vehicle MI; ##p indicates AAV.SIK2 Vehicle MI vs. AAV.SIK2 SB 203580 MI.

N = number, LVEDD = left ventricular end-diastolic diameter; LVESD = left ventricular end-systolic diameter; FS = fractional shortening; LVEDV = left ventricular end-diastolic volume; LVESV = left ventricular end -systolic volume; EF = ejection fraction; HR = heart rate.

Table S6. Biometric and cardiac parameters of Wild-type (WT) mice administrated with adeno-associated virus (AAV).F4/80.SIK2^{T175A} or AAV.F4/80.NC on days 14 post-MI.

Group	AAV.NC Sham	AAV.SIK2^{T175A} Sham	AAV.NC MI	AAV.SIK2^{T175A} MI
N	6	6	6	6
LVEDD (mm)	3.97±0.07	3.93±0.10	5.09±0.09	5.03±0.08$^{p=0.853}$
LVESD (mm)	2.83±0.05	2.77±0.08	4.64±0.07	4.53±0.09$^{p=0.553}$
FS (%)	28.71±0.30	29.62±0.49	8.90±0.67	10.02±0.76$^{p=0.378}$
LVEDV (μL)	68.91±2.95	67.49±3.91	123.70±4.99	120.25±4.19$^{p=0.803}$
LVESV (μL)	30.43±1.45	28.97±2.15	99.44±3.68	94.17±4.49$^{p=0.445}$
EF (%)	55.87±0.47	57.27±0.80	19.50±1.37	21.81±1.55$^{p=0.301}$
HR (beats/min)	486.11±5.21	479.60±7.96	489.77±6.96	484.66±10.54

Data are shown as mean ± SEM. Data were analyzed using two-way ANOVA with Holm-Sidak's multiple comparisons test. P indicates AAV.NC MI vs. AAV.SIK2^{T175A} MI.

N = number, LVEDD = left ventricular end-diastolic diameter; LVESD = left ventricular end-systolic diameter; FS = fractional shortening; LVEDV = left ventricular end-diastolic volume; LVESV = left ventricular end -systolic volume; EF = ejection fraction; HR = heart rate.

Table S7. Biometric and cardiac parameters of ARN3236 treated or vehicle mice on days 14 post-MI.

Group	Vehicle Sham	ARN3236 Sham	Vehicle MI	ARN3236 MI
N	6	6	6	6
LVEDD (mm)	3.75±0.12	3.62±0.09	5.36±0.10	4.80±0.10$^{**p=0.002}$
LVESD (mm)	2.58±0.10	2.48±0.08	4.87±0.10	4.17±0.09$^{**p<0.001}$
FS (%)	32.32±0.96	31.47±1.04	9.16±0.65	12.98±1.03$^{*p=0.018}$
LVEDV (μL)	60.71±4.42	55.52±3.41	139.37±5.91	107.62±5.55$^{**p<0.001}$
LVESV (μL)	24.41±2.16	22.13±1.70	111.59±5.08	77.67±4.29$^{**p<0.001}$
EF (%)	59.91±1.41	60.21±1.50	19.95±1.34	27.78±2.01$^{**p=0.005}$
HR (beats/min)	499.68±2.89	489.10±9.34	482.04±5.51	476.04±5.53

Data are shown as mean ± SEM. Data were analyzed using two-way ANOVA with Holm-Sidak's multiple comparisons test. *, **p indicates Vehicle MI vs. ARN3236 MI. N = number, LVEDD = left ventricular end-diastolic diameter; LVESD = left ventricular end-systolic diameter; FS = fractional shortening; LVEDV = left ventricular end-diastolic volume; LVESV = left ventricular end -systolic volume; EF = ejection fraction; HR = heart rate.

Table S8. Biometric and cardiac parameters of Traf2$^{+/-}$ and WT mice on days 14 post-MI.

Group	WT<br>Sham	Traf2$^{+/-}$<br>Sham	WT<br>MI	Traf2$^{+/-}$<br>MI
N	6	6	6	6
LVEDD (mm)	3.81±0.08	3.70±0.09	5.64±0.13	5.01±0.12$^{**p<0.001}$
LVESD (mm)	2.66±0.08	2.61±0.08	5.20±0.11	4.41±0.10$^{**p<0.001}$
FS (%)	30.38±0.64	29.48±0.69	7.74±0.72	10.94±0.88$^{*p=0.012}$
LVEDV (μL)	62.71±3.27	58.34±3.30	156.54±8.11	119.05±6.51$^{**p<0.001}$
LVESV (μL)	26.16±1.85	25.02±1.80	129.86±6.47	91.15±6.30$^{**p<0.001}$
EF (%)	58.50±0.99	57.27±1.08	16.92±1.49	23.67±1.78$^{**p=0.005}$
HR (beats/min)	476.99±7.63	482.03±3.45	464.39±9.17	480.50±7.65

Data are shown as mean ± SEM. Data were analyzed using two-way ANOVA with Holm-Sidak's multiple comparisons test. *, **p indicates WT MI vs. Traf2$^{+/-}$ MI.

N = number, LVEDD = left ventricular end-diastolic diameter; LVESD = left ventricular end-systolic diameter; FS = fractional shortening; LVEDV = left ventricular end-diastolic volume; LVESV = left ventricular end -systolic volume; EF = ejection fraction; HR = heart rate.

Table S9. Primers for real-time quantitative PCR

Gene	Species	Sense primer	Anti-sense primer
GAPDH	Human	GGAGCGAGATCCCTCCAAAAT	GGCTGTTGTCATACTTCTCATGG
SIK2	Human	AGACCACCCTCACATAATCAAAC	ATTTTCGCCTGGCTTCAGACT
ARG1	Human	GTGGAAACTTGCATGGACAAC	AATCCTGGCACATCGGGAATC
Gapdh	Mouse	AGGTCGGTGTGAACGGATTTG	TGTAGACCATGTAGTTGAGGTCA
Sik1	Mouse	TCATGTCGGAGTTCAGTGCG	ACCTGCGTTTTGGTGACTCG
Sik2	Mouse	CTGCTGGCAACATGGTGTG	GGGAGAGTTGGTCCATCAAAAG
Sik3	Mouse	GCCATCCACACATCATCAGAC	CCAAGTGGTCAAATATCTCCCC
Acta2	Mouse	TCCTGACGCTGAAGTATCCGATA	GGCCACACGAAGCTCGTTAT
Col1a1	Mouse	GAGTACTGGATCGACCCTAACCA	GACGGCTGAGTAGGGAACACA
Col3a1	Mouse	TCCCCTGGAATCTGTGAATC	TGAGTCGAATTGGGGAGAAT
Il6	Mouse	GCTACCAAACTGGATATAATCAGGA	CCAGGTAGCTATGGTACTCCAGAA
Tnfα	Mouse	GAGTGACAAGCCTGTAGCC	CTCCTGGTATGAGATAGCAAA
Mmp9	Mouse	CGTCGTGATCCCCACTTACT	AACACACAGGGTTTGCCTTC
Il1α	Mouse	TCTGAAGAAGAGACGGCTGA	CTGATCTGGGTTGGATGGTC
Il1β	Mouse	CTTCCCCAGGGCATGTTAAG	ACCCTGAGCGACCTGTCTTG
Cxcl1	Mouse	CTGGGATTCACCTCAAGAACATC	CAGGGTCAAGGCAAGCCTC
Nos2	Mouse	CACCAAGCTGAACTTGAGCG	CGTGGCTTTGGGCTCCTC
Tgfb1	Mouse	TACCATGCCAACTTCTGTCTGGGA	ATGTTGGACAACTGCTCCACCTTG
Vegfa	Mouse	CTGCTGTAACGATGAAGCCCTG	GCTGTAGGAAGCTCATCTCTCC
Il10	Mouse	GCTCTTACTGACTGGCATGAG	CGCAGCTCTAGGAGCATGTG
Arg1	Mouse	CCAGAAGAATGGAAGAGTCAGTGT	GCAGATATGCAGGGAGTCACC
Cd36	Mouse	AGATGACGTGGCAAAGAACAG	CCTTGGCTAGATAACGAACTCTG
Mgl1	Mouse	CAATGTGGTTAGTTGGATCGGC	CCCAGTTCTTAAAGCCTTTCTCA
Cx3cr1	Mouse	GAGTATGACGATTCTGCTGAGG	CAGACCGAACGTGAAGACGAG
Mfge8	Mouse	CAGCCGTCCCCAATACTGC	GGGAGGCTAGGTTGTTGGAA
Mertk	Mouse	CAGGGCCTTTACCAGGGAGA	TGTGTGCTGGATGTGATCTTC

Timd4	Mouse	CCGGTGACTTTGCCTTGTCAT	CTCTGCATTGCACTTGGAATTG
